# Supplementary material for: Transcriptomic metaanalyses of autistic brains reveals shared gene expression and biological pathway abnormalities with cancer
Source: Mol Autism. 2019 Apr 8;10:17. doi: 10.1186/s13229-019-0262-8 (PMC6454734; doi:10.1186/s13229-019-0262-8)
Supplement: Supplementary file 1 — Table S1. Datasets included in our study. Table S2. Age, gender and pmi distributions between cases and control samples at each step of the preprocessing procedure at the asd defferentail gene expression meta-analysis. Table S3. Final samples included in the asd meta-analysis. Table S4. Jointly same direction derregulated genes in asd ans sddcs and jointly oposite direction derregulated genes in asd and oddcs. Table S5. Genes jointly deregulated in asd and in sddcs and oddcs. Figure S1. Comaprison of differential gene expression analysis using limma with rlog transformed data and two state of the art rnaseq differential expression metdos. Figure S2. Patient overlap in the three asd studies. Figure S3. Metaqc and mean intersample correlation results. Figure S4. Partition around medoids (pam) clustering of the differential gene expression profiles of asd and the 22 included cancer types. Figure S5. Top upregulated and downregulated pathways in cancer (halmmarks molecular signature). Figure S6. Top upregulated and downretualted pathways in cancer (c2 canonical pathways molecular signature). Figure S7. Intersection analysis of the two control diseases (ulcerative colitis and malaria). Figure S8. Percentaje of pathways deregulated in the same or opposite directions between asd and cancer (DOCX 3835 kb) [file 13229_2019_262_MOESM1_ESM.docx]

**TABLE OF CONTENTS**

**SUPPLEMENTARY TABLES:**

SUPPLEMENTARY TABLE 1: DATASETS INCLUDED IN OUR STUDY.

SUPPLEMENTARY TABLE 2: AGE, GENDER AND PMI DISTRIBUTIONS BETWEEN CASES AND CONTROL SAMPLES AT EACH STEP OF THE PREPROCESSING PROCEDURE AT THE ASD DEFFERENTAIL GENE EXPRESSION META-ANALYSIS.

SUPPLEMENTARY TABLE 3: FINAL SAMPLES INCLUDED IN THE ASD META-ANALYSIS.

SUPPLEMEMTARY TABLE 4: JOINTLY SAME DIRECTION DERREGULATED GENES IN ASD ANS SDDCs AND JOINTLY OPOSITE DIRECTION DERREGULATED GENES IN ASD AND ODDCs.

SUPPLEMENTARY TABLE 5: GENES JOINTLY DEREGULATED IN ASD AND IN SDDCs AND ODDCs.

**SUPPLEMENTARY FIGURES:**

SUPPLMENTARY FIGURE 1: COMAPRISON OF DIFFERENTIAL GENE EXPRESSION ANALYSIS USING LIMMA WITH RLOG TRANSFORMED DATA AND TWO STATE OF THE ART RNASEQ DIFFERENTIAL EXPRESSION METDOS.

SUPPLEMENTARY FIGURE 2: PATIENT OVERLAP IN THE THREE ASD STUDIES.

SUPPLEMENTARY FIGURE 3: METAQC AND MEAN INTERSAMPLE CORRELATION RESULTS.

SUPPLEMENTARY FIGURE 4: PARTITION AROUND MEDOIDS (PAM) CLUSTERING OF THE DIFFERENTIAL GENE EXPRESSION PROFILES OF ASD AND THE 22 INCLUDED CANCER TYPES.

SUPPLEMENTARY FIGURE 5: TOP UPREGULATED AND DOWNREGULATED PATHWAYS IN CANCER (HALMMARKS MOLECULAR SIGNATURE).

SUPPLEMENTARY FIGURE 6: TOP UPREGULATED AND DOWNRETUALTED PATHWAYS IN CANCER (C2 CANONICAL PATHWAYS MOLECULAR SIGNATURE).

SUPPLEMENTARY FIGURE 7: INTERSECTION ANALYSIS OF THE TWO CONTROL DISEASES (ULCERATIVE COLITIS AND MALARIA)

SUPPLEMENTARY FIGURE 8: PERCENTAJE OF PATHWAYS DEREGULATED IN THE SAME OR OPPOSITE DIRECTIONS BETWEEN ASD AND CANCER

**CODE APPENDIX:**

AI.1) GSE28521 PREPROCESSING

AI.2) GSE28475 PREPROCESSING

AI.3) GUPTA’S PREPROCESSING

AI.4) ASD DIFFERENTAIL GENE EXPRESSION META-NALYSIS

AI.5) FUNCTIONS

**SUPPLEMENTARY TABLES**

**Table S1:**

| Cancer.type | Study | Initial samples | Final after quality control | Cases | | Controls | Number of genes studied | PubMed.ID | Year | Platform |
| --- | --- | --- | --- | --- | --- | --- | --- | --- | --- | --- |
| ALL | GSE13204 | 3248 | 824 | | 750 | 74 | 20503 | 18573112 | 2008 | Affymetrix Human Genome U133 Plus 2.0 Array |
| ALL | GSE26713 | 124 | 124 | | 117 | 7 | 20503 | 21481790 | 2011 | Affymetrix Human Genome U133 Plus 2.0 Array |
| ALL | GSE41621 | 20 | 19 | | 17 | 2 | 21026 | 23762353 | 2013 | Illumina HumanHT-12 V4.0 expression beadchip |
| ALL | GSE46170 | 38 | 38 | | 32 | 6 | 20503 | 24632884 | 2014 | Affymetrix Human Genome U133 Plus 2.0 Array |
| ALL | GSE79533 | 229 | 226 | | 223 | 3 | 20503 | 27634205 | 2017 | Affymetrix Human Genome U133 Plus 2.0 Array |
| AML | GSE13204 | 3248 | 616 | | 542 | 74 | 20536 | 18573112 | 2008 | Affymetrix Human Genome U133 Plus 2.0 Array |
| AML | GSE12662 | 106 | 106 | | 91 | 15 | 20536 | 19451695 | 2009 | Affymetrix Human Genome U133 Plus 2.0 Array |
| AML | GSE30029 | 121 | 121 | | 90 | 31 | 19305 | 21760593 | 2011 | Illumina HumanHT-12 V3.0 expression beadchip |
| AML | GSE34577 | 89 | 89 | | 71 | 18 | 19305 | 22289410 | 2012 | Affymetrix Human Gene 1.0 ST Array |
| AML | GSE48558 | 170 | 39 | | 18 | 21 | 18720 | 23836560 | 2013 | Affymetrix Human Gene 1.0 ST Array |
| AML | GSE63270 | 104 | 104 | | 62 | 42 | 20536 | 26444494 | 2015 | Affymetrix Human Genome U133 Plus 2.0 Array_Mod_1 |
| AML | GSE76340 | 166 | 23 | | 15 | 8 | 19213 | 26964570 | 2016 | Illumina HumanHT-12 V3.0 expression beadchip/Illumina HumanHT-12 V4.0 expression beadchip |
| AML | GSE67936 | 168 | 168 | | 150 | 18 | 21028 | NA | NA | Illumina HumanHT-12 V4.0 expression beadchip_Mod_1 |
| AML | GSE68172 | 77 | 59 | | 54 | 5 | 20536 | NA | NA | Affymetrix Human Genome U133 Plus 2.0 Array |
| ASD | GSE28475 + GSE36192 | 143 + 40 | 65 | | 8 | 57 | 17631 | 22375143 | 2012 | Illumina HumanRef-8 v3.0 expression beadchip |
| ASD | Gupta | 39 | 27 | | 10 | 17 | 15690 | 25494366 | 2014 | Illumina HiSeq |
| ASD | GSE28521 +  GSE36192 | 79 + 40 | 72 | | 16 | 56 | 17631 | 21614001 | 2011 | Illumina HumanRef-8 v3.0 expression beadchip |
| Bladder cancer | GSE7476 | 12 | 12 | | 9 | 3 | 20503 | 19539325 | 2009 | Affymetrix Human Genome U133 Plus 2.0 Array |
| Bladder cancer | GSE13507 | 256 | 84 | | 75 | 9 | 19019 | 20059769 | 2010 | Illumina human-6 v2.0 expression beadchip |
| Bladder cancer | GSE31189 | 92 | 27 | | 16 | 11 | 20503 | 23097579 | 2012 | Affymetrix Human Genome U133 Plus 2.0 Array |
| Bladder cancer | E_MTAB_1940 | 86 | 84 | | 82 | 4 | 20503 | 25456126 | 2014 | Affymetrix Human Genome U133 Plus 2.0 Array |
| Bladder cancer | GSE37815 | 24 | 16 | | 11 | 5 | 19019 | 24072181 | 2014 | Illumina human-6 v2.0 expression beadchip |
| Bladder cancer | GSE38264 | 51 | 38 | | 28 | 10 | 18994 | 25252918 | 2014 | Affymetrix Human Gene 1.0 ST Array |
| Bladder cancer | GSE52519 | 12 | 8 | | 5 | 3 | 19293 | NA | NA | Illumina HumanWG-6 v3.0 expression beadchip |
| Bladder cancer | GSE65635 | 12 | 10 | | 6 | 4 | 19453 | NA | NA | Illumina HumanHT-12 WG-DASL V4.0 R2 expression beadchip |
| Brain cancer | GSE4290 | 180 | 180 | | 153 | 27 | 20536 | 16616334 | 2006 | Affymetrix Human Genome U133 Plus 2.0 Array |
| Brain cancer | GSE9385 | 55 | 55 | | 49 | 6 | 21243 | 17575129 | 2007 | Affymetrix Human Exon 1.0 ST Array |
| Brain cancer | GSE74195 | 51 | 44 | | 39 | 5 | 20536 | 18577562 | 2008 | Affymetrix Human Genome U133 Plus 2.0 Array |
| Brain cancer | GSE68848 | 580 | 482 | | 454 | 28 | 20536 | 19208739 | 2009 | Affymetrix Human Genome U133 Plus 2.0 Array |
| Brain cancer | GSE15824 | 45 | 35 | | 30 | 5 | 20536 | 21406405 | 2011 | Affymetrix Human Genome U133 Plus 2.0 Array |
| Brain cancer | GSE42656 | 73 | 64 | | 53 | 11 | 19305 | 23887970 | 2013 | Illumina HumanHT-12 V3.0 expression beadchip |
| Brain cancer | GSE44971 | 58 | 58 | | 49 | 9 | 20536 | 23660940 | 2013 | Affymetrix Human Genome U133 Plus 2.0 Array |
| Brain cancer | GSE50161 | 130 | 128 | | 115 | 13 | 20536 | 24078694 | 2013 | Affymetrix Human Genome U133 Plus 2.0 Array |
| Breast cancer | GSE7904 | 62 | 47 | | 40 | 7 | 20536 | 16473279 | 2006 | Affymetrix Human Genome U133 Plus 2.0 Array |
| Breast cancer | GSE10780 | 185 | 185 | | 42 | 143 | 20536 | 19266279 | 2010 | Affymetrix Human Genome U133 Plus 2.0 Array |
| Breast cancer | GSE10810 | 58 | 58 | | 31 | 27 | 20536 | 20029976 | 2010 | Affymetrix Human Genome U133 Plus 2.0 Array |
| Breast cancer | GSE31448 | 357 | 356 | | 352 | 4 | 20536 | 22110708 | 2011 | Affymetrix Human Genome U133 Plus 2.0 Array |
| Breast cancer | GSE42568 | 121 | 114 | | 98 | 16 | 20536 | 23740839 | 2013 | Affymetrix Human Genome U133 Plus 2.0 Array |
| Breast cancer | GSE54002 | 433 | 433 | | 417 | 16 | 20536 | 25214461 | 2014 | Affymetrix Human Genome U133 Plus 2.0 Array |
| Breast cancer | GSE65216 | 356 | 347 | | 330 | 17 | 20536 | 25848952 | 2015 | Affymetrix Human Genome U133 Plus 2.0 Array |
| Breast cancer | GSE45827 | 155 | 149 | | 140 | 9 | 20536 | 27006338 | 2016 | Affymetrix Human Genome U133 Plus 2.0 Array |
| Breast cancer | GSE29431 | 66 | 54 | | 44 | 10 | 20536 | NA | NA | Affymetrix Human Genome U133 Plus 2.0 Array |
| Cervical cancer | GSE6791 | 84 | 28 | | 20 | 8 | 20503 | 17510386 | 2007 | Affymetrix Human Genome U133 Plus 2.0 Array |
| Cervical cancer | GSE29570 | 62 | 62 | | 45 | 17 | 18994 | 22357541 | 2012 | Affymetrix Human Gene 1.0 ST Array |
| Cervical cancer | GSE39001 | 79 | 24 | | 19 | 5 | 18994 | 23405241 | 2013 | Affymetrix Human Gene 1.0 ST Array |
| Cervical cancer | GSE52904 | 199 | 72 | | 55 | 17 | 18994 | 24879114 | 2014 | Affymetrix Human Gene 1.0 ST Array |
| Cervical cancer | GSE63514 | 128 | 128 | | 104 | 24 | 20503 | 26056290 | 2015 | Affymetrix Human Genome U133 Plus 2.0 Array |
| Cervical cancer | GSE67522 | 42 | 35 | | 15 | 20 | 19729 | 26152361 | 2015 | Illumina HumanHT-12 V4.0 expression beadchip |
| Cholangiocarcinoma | GSE22633 | 64 | 24 | | 20 | 4 | 19019 | 21333016 | 2011 | Illumina human-6 v2.0 expression beadchip |
| Cholangiocarcinoma | GSE26566 | 169 | 17 | | 11 | 6 | 17290 | 22178589 | 2012 | Illumina humanRef-8 v2.0 expression beadchip |
| Cholangiocarcinoma | GSE31370 | 26 | 7 | | 3 | 4 | 21026 | 22234953 | 2012 | Illumina HumanHT-12 V4.0 expression beadchip |
| Cholangiocarcinoma | GSE32879 | 37 | 23 | | 16 | 7 | 18994 | 22707408 | 2012 | Affymetrix Human Gene 1.0 ST Array |
| Cholangiocarcinoma | GSE32225 | 155 | 88 | | 83 | 5 | 17631 | 23295441 | 2013 | Illumina HumanRef-8 WG-DASL v3.0 |
| Cholangiocarcinoma | GSE45001 | 20 | 11 | | 6 | 5 | 21755 | 23775819 | 2013 | Agilent-028004 SurePrint G3 Human GE 8x60K Microarray |
| Cholangiocarcinoma | GSE57555 | 64 | 10 | | 2 | 8 | 21755 | 26538415 | 2015 | Agilent-039494 SurePrint G3 Human GE v2 8x60K Microarray 039381 |
| CLL | GSE13204 | 3248 | 522 | | 448 | 74 | 20503 | 18573112 | 2008 | Affymetrix Human Genome U133 Plus 2.0 Array |
| CLL | GSE13987 | 24 | 8 | | 4 | 4 | 20536 | 19380787 | 2009 | Affymetrix Human Genome U133 Plus 2.0 Array |
| CLL | GSE26725 | 17 | 17 | | 12 | 5 | 20536 | 21296997 | 2011 | Affymetrix Human Genome U133 Plus 2.0 Array |
| CLL | GSE36907 | 65 | NA | | 9 | 10 | 18720 | 23091163 | 2012 | NA |
| CLL | GSE31048 | 221 | 221 | | 188 | 33 | 20536 | 24778153 | 2014 | Affymetrix Human Genome U133 Plus 2.0 Array |
| CLL | GSE51528 | 229 | 229 | | 217 | 12 | 18720 | 25860243 | 2015 | Affymetrix Human Gene 1.0 ST Array |
| CLL | GSE67640 | 24 | 24 | | 15 | 9 | 21042 | 27081174 | 2016 | Illumina HumanHT-12 V4.0 expression beadchip |
| CML | GSE13204 | 3248 | 150 | | 76 | 74 | 20503 | 18573112 | 2008 | Affymetrix Human Genome U133 Plus 2.0 Array |
| CML | GSE43754 | 20 | 20 | | 10 | 10 | 22870 | 23651669 | 2013 | Affymetrix Human Exon 1.0 ST Array |
| CML | GSE47927 | 67 | 67 | | 52 | 15 | 18994 | 23836560 | 2013 | Affymetrix Human Gene 1.0 ST Array |
| CML | GSE77573 | 79 | 40 | | 36 | 4 | 23516 | NA | NA | Affymetrix Human Transcriptome Array 2.0 |
| Colorectal cancer | GSE41328 | 20 | 20 | | 10 | 10 | 20503 | 17160039 | 2006 | Affymetrix Human Genome U133 Plus 2.0 Array |
| Colorectal cancer | GSE9348 | 82 | 82 | | 70 | 12 | 20503 | 20143136 | 2010 | Affymetrix Human Genome U133 Plus 2.0 Array |
| Colorectal cancer | GSE20916 | 145 | 145 | | 111 | 34 | 20503 | 20957034 | 2010 | Affymetrix Human Genome U133 Plus 2.0 Array |
| Colorectal cancer | GSE33113 | 96 | 96 | | 90 | 6 | 20503 | 22056143 | 2011 | Affymetrix Human Genome U133 Plus 2.0 Array |
| Colorectal cancer | GSE21510 | 148 | 148 | | 123 | 25 | 20503 | 21270110 | 2011 | Affymetrix Human Genome U133 Plus 2.0 Array |
| Colorectal cancer | GSE24550 | 167 | 90 | | 77 | 13 | 22870 | 21619627 | 2011 | Affymetrix Human Exon 1.0 ST Array |
| Colorectal cancer | GSE21815 | 141 | 129 | | 120 | 9 | 19596 | 21862635 | 2011 | Agilent-014850 Whole Human Genome Microarray 4x44K G4112F |
| Colorectal cancer | GSE31279 | 110 | 53 | | 30 | 23 | 17290 | 22210045 | 2012 | Illumina humanRef-8 v2.0 expression beadchip |
| Colorectal cancer | GSE39582 | 585 | 585 | | 566 | 19 | 20503 | 23700391 | 2013 | Affymetrix Human Genome U133 Plus 2.0 Array |
| Colorectal cancer | GSE37182 | 172 | 168 | | 84 | 84 | 19293 | 23308215 | 2013 | Illumina HumanHT-12 V3.0 expression beadchip |
| Colorectal cancer | GSE35834 | 158 | 53 | | 30 | 23 | 22870 | 23987127 | 2013 | Affymetrix Human Exon 1.0 ST Array |
| Colorectal cancer | GSE31905 | 67 | 62 | | 55 | 7 | 19596 | 22012870 | 2013 | Agilent-014850 Whole Human Genome Microarray 4x44K G4112F |
| Colorectal cancer | GSE37364 | 94 | 52 | | 14 | 38 | 20503 | 25405986 | 2014 | Affymetrix Human Genome U133 Plus 2.0 Array |
| Colorectal cancer | GSE44076 | 246 | 246 | | 98 | 148 | 18553 | 24597571 | 2014 | Affymetrix Human Genome U219 Array |
| Colorectal cancer | GSE62932 | 68 | 68 | | 64 | 4 | 20503 | 27176004 | 2016 | Affymetrix Human Genome U133 Plus 2.0 Array |
| Colorectal cancer | GSE68204 | 125 | 76 | | 57 | 19 | 19596 | 27225591 | 2017 | NA |
| Colorectal cancer | GSE74602 | 60 | 60 | | 30 | 30 | 17290 | NA | NA | Illumina humanRef-8 v2.0 expression beadchip |
| DLBCL | GSE12453 | 67 | 36 | | 11 | 25 | 20503 | 18794340 | 2008 | Affymetrix Human Genome U133 Plus 2.0 Array |
| DLBCL | GSE12195 | 136 | 88 | | 73 | 15 | 20503 | 19412164 | 2009 | Affymetrix Human Genome U133 Plus 2.0 Array |
| DLBCL | GSE56315 | 122 | 122 | | 89 | 33 | 20503 | 25800755 | 2015 | Affymetrix Human Genome U133 Plus 2.0 Array |
| Follicular lymphoma | GSE12453 | 67 | 30 | | 5 | 25 | 20536 | 18794340 | 2008 | Affymetrix Human Genome U133 Plus 2.0 Array |
| Follicular lymphoma | GSE12195 | 136 | 53 | | 38 | 15 | 20536 | 19412164 | 2009 | Affymetrix Human Genome U133 Plus 2.0 Array |
| Follicular lymphoma | GSE35331 | 28 | 22 | | 10 | 12 | 20536 | 22289889 | 2012 | Affymetrix Human Genome U133 Plus 2.0 Array |
| Follicular lymphoma | GSE55267 | 69 | 69 | | 63 | 6 | 20536 | 24634383 | 2014 | Affymetrix Human Genome U133 Plus 2.0 Array |
| Follicular lymphoma | GSE61912 | 30 | 20 | | 16 | 4 | 18720 | 25607463 | 2015 | Affymetrix Human Gene 1.0 ST Array |
| Follicular lymphoma | GSE65135 | 28 | 24 | | 14 | 10 | 20536 | 25822800 | 2015 | Affymetrix Human Genome U133 Plus 2.0 Array |
| Follicular lymphoma | GSE48047 | 55 | 26 | | 18 | 8 | 21755 | NA | NA | Agilent-028004 SurePrint G3 Human GE 8x60K Microarray |
| Gastric cancer | GSE13911 | 69 | 69 | | 38 | 31 | 20536 | 19081245 | 2009 | Affymetrix Human Genome U133 Plus 2.0 Array |
| Gastric cancer | GSE27342 | 160 | 146 | | 73 | 73 | 20923 | 20965966 | 2011 | Affymetrix Human Exon 1.0 ST Array |
| Gastric cancer | GSE29998 | 99 | 99 | | 50 | 49 | 19305 | 21781349 | 2011 | Illumina HumanHT-12 V3.0 expression beadchip |
| Gastric cancer | GSE33335 | 50 | 50 | | 25 | 25 | 20923 | 22559327 | 2012 | Affymetrix Human Exon 1.0 ST Array |
| Gastric cancer | GSE19826 | 27 | 27 | | 12 | 15 | 20536 | 21132402 | 2012 | Affymetrix Human Genome U133 Plus 2.0 Array |
| Gastric cancer | GSE63089 | 90 | 89 | | 45 | 44 | 20923 | 25646628 | 2015 | Affymetrix Human Exon 1.0 ST Array |
| Gastric cancer | GSE51575 | 52 | 36 | | 15 | 21 | 19722 | 25254613 | 2015 | Agilent-028004 SurePrint G3 Human GE 8x60K Microarray |
| Gastric cancer | GSE79973 | 20 | 20 | | 10 | 10 | 20536 | 27785057 | 2016 | Affymetrix Human Genome U133 Plus 2.0 Array |
| Gastric cancer | GSE26899 | 108 | 52 | | 41 | 11 | 19305 | NA | NA | Illumina HumanHT-12 V3.0 expression beadchip |
| Gastric cancer | GSE54129 | 132 | 132 | | 111 | 21 | 20536 | NA | NA | Affymetrix Human Genome U133 Plus 2.0 Array |
| Gastric cancer | GSE13195 | 100 | 50 | | 25 | 25 | 20923 | NA | NA | Affymetrix Human Exon 1.0 ST Array |
| Gastric cancer | GSE30727 | 52 | 36 | | 30 | 30 | 20923 | NA | NA | Affymetrix Human Exon 1.0 ST Array |
| Gastric cancer | GSE84787 | 20 | 6 | | 3 | 3 | 21755 | NA | NA | Agilent-039494 SurePrint G3 Human GE v2 8x60K Microarray |
| Head and neck carcinoma | GSE30784 | 229 | 212 | | 167 | 45 | 20536 | 18669583 | 2008 | Affymetrix Human Genome U133 Plus 2.0 Array |
| Head and neck carcinoma | GSE31056 | 96 | 96 | | 23 | 73 | 20536 | 21989116 | 2011 | Affymetrix Human Genome U133 Plus 2.0 Array |
| Head and neck carcinoma | GSE25099 | 79 | 79 | | 57 | 22 | 20923 | 21619627 | 2011 | Affymetrix Human Exon 1.0 ST Array |
| Head and neck carcinoma | GSE34105 | 78 | 57 | | 49 | 8 | 19462 | 22530001 | 2012 | Illumina HumanHT-12 WG-DASL V4.0 R2 expression beadchip |
| Head and neck carcinoma | GSE42743 | 103 | 103 | | 74 | 29 | 20536 | 23319825 | 2013 | Affymetrix Human Genome U133 Plus 2.0 Array |
| Head and neck carcinoma | GSE33205 | 69 | 40 | | 21 | 19 | 20923 | 24351288 | 2014 | Affymetrix Human Exon 1.0 ST Array |
| Head and neck carcinoma | GSE92396 | 22 | 22 | | 12 | 10 | 18720 | 24072181 | 2014 | Affymetrix Human Gene 1.0 ST Array |
| Head and neck carcinoma | GSE75538 | 28 | 10 | | 5 | 5 | 19462 | 27288358 | 2016 | Illumina HumanHT-12 WG-DASL V4.0 R2 expression beadchip |
| Head and neck carcinoma | GSE55550 | 155 | 86 | | 73 | 13 | 21755 | 25899179 | 2016 | Agilent-039494 SurePrint G3 Human GE v2 8x60K Microarray 039381 |
| Head and neck carcinoma | GSE29330 | 18 | 18 | | 13 | 5 | 20536 | NA | NA | Affymetrix Human Genome U133 Plus 2.0 Array |
| Head and neck carcinoma | GSE59102 | 42 | 32 | | 23 | 9 | 19596 | NA | NA | Agilent-014850 Whole Human Genome Microarray 4x44K G4112F |
| Kidney cancer | GSE11151 | 67 | 57 | | 52 | 5 | 20536 | 19445733 | 2009 | Affymetrix Human Genome U133 Plus 2.0 Array |
| Kidney cancer | GSE77199 | 43336 | 6 | | 3 | 3 | 21755 | 19380787 | 2009 | Agilent-028004 SurePrint G3 Human GE 8x60K Microarray |
| Kidney cancer | GSE47032 | 40 | 20 | | 10 | 10 | 22870 | 24194935 | 2013 | Affymetrix Human Exon 1.0 ST Array |
| Kidney cancer | GSE53757 | 144 | 144 | | 72 | 72 | 20536 | 24962026 | 2014 | Affymetrix Human Genome U133 Plus 2.0 Array |
| Kidney cancer | GSE53000 | 62 | 62 | | 56 | 6 | 18720 | 24487277 | 2014 | Affymetrix Human Gene 1.0 ST Array |
| Kidney cancer | GSE66272 | 54 | 54 | | 27 | 27 | 20536 | 26859141 | 2016 | Affymetrix Human Genome U133 Plus 2.0 Array |
| Kidney cancer | GSE68417 | 49 | 49 | | 35 | 14 | 18720 | 26670202 | 2016 | Affymetrix Human Gene 1.0 ST Array |
| Kidney cancer | GSE71963 | 48 | 48 | | 32 | 16 | 19596 | 26790128 | 2016 | Agilent-014850 Whole Human Genome Microarray 4x44K G4112F |
| Kidney cancer | GSE40435 | 202 | 202 | | 101 | 101 | 21029 | NA | NA | Illumina HumanHT-12 V4.0 expression beadchip |
| Kidney cancer | GSE76351 | 24 | 24 | | 12 | 12 | 18720 | NA | NA | Affymetrix Human Gene 1.1 ST Array |
| Liver cancer | GSE6764 | 75 | 75 | | 65 | 10 | 20536 | 17393520 | 2007 | Affymetrix Human Genome U133 Plus 2.0 Array |
| Liver cancer | GSE12941 | 20 | 20 | | 10 | 10 | 20923 | 20388846 | 2010 | Affymetrix Human Exon 1.0 ST Array |
| Liver cancer | GSE76427 | 167 | 101 | | 50 | 51 | 21041 | 21619627 | 2011 | Illumina HumanHT-12 V4.0 expression beadchip |
| Liver cancer | GSE56140 | 69 | 48 | | 14 | 34 | 19299 | 21320499 | 2011 | Illumina HumanHT-12 V3.0 expression beadchip |
| Liver cancer | GSE50579 | 80 | 34 | | 26 | 8 | 21755 | 22689435 | 2012 | Agilent-028004 SurePrint G3 Human GE 8x60K Microarray |
| Liver cancer | GSE32879 | 37 | 37 | | 30 | 7 | 18720 | 22707408 | 2012 | Affymetrix Human Gene 1.0 ST Array |
| Liver cancer | GSE36376 | 433 | 273 | | 80 | 193 | 21042 | 23800896 | 2013 | Illumina HumanHT-12 V4.0 expression beadchip |
| Liver cancer | GSE41804 | 40 | 40 | | 20 | 20 | 20536 | 23426277 | 2013 | Affymetrix Human Genome U133 Plus 2.0 Array |
| Liver cancer | GSE17548 | 37 | 37 | | 17 | 20 | 20536 | 23691139 | 2013 | Affymetrix Human Genome U133 Plus 2.0 Array |
| Liver cancer | GSE57957 | 78 | 67 | | 28 | 39 | 21042 | 25093504 | 2014 | Illumina HumanHT-12 V4.0 expression beadchip |
| Liver cancer | GSE46444 | 136 | 58 | | 37 | 21 | 21042 | 24072181 | 2014 | Illumina HumanHT-12 V4.0 expression beadchip |
| Liver cancer | GSE39791 | 144 | 92 | | 24 | 68 | 21042 | 25536056 | 2014 | Illumina HumanHT-12 V4.0 expression beadchip |
| Liver cancer | GSE55092 | 140 | 44 | | 20 | 24 | 20536 | 25141867 | 2014 | Affymetrix Human Genome U133 Plus 2.0 Array |
| Liver cancer | GSE62232 | 91 | 86 | | 81 | 5 | 20536 | 25822088 | 2015 | Affymetrix Human Genome U133 Plus 2.0 Array |
| Liver cancer | GSE57727 | 62 | 31 | | 28 | 3 | 19460 | 25557953 | 2015 | Illumina HumanHT-12 WG-DASL V4.0 R2 expression beadchip |
| Liver cancer | GSE54236 | 161 | 133 | | 55 | 78 | 19596 | 25666192 | 2016 | Agilent-014850 Whole Human Genome Microarray 4x44K G4112F |
| Liver cancer | GSE64041 | 125 | 125 | | 60 | 65 | 18720 | 27499918 | 2016 | Affymetrix Human Gene 1.0 ST Array |
| Liver cancer | GSE36411 | 84 | 84 | | 42 | 42 | 21042 | NA | NA | Illumina HumanHT-12 V4.0 expression beadchip |
| Liver cancer | GSE51401 | 64 | 64 | | 30 | 34 | 20536 | NA | NA | Affymetrix Human Genome U133 Plus 2.0 Array |
| Lung cancer | GSE19188 | 156 | 156 | | 91 | 65 | 20536 | 20421987 | 2010 | Affymetrix Human Genome U133 Plus 2.0 Array |
| Lung cancer | GSE19804 | 120 | 120 | | 60 | 60 | 20536 | 20802022 | 2010 | Affymetrix Human Genome U133 Plus 2.0 Array |
| Lung cancer | GSE18842 | 91 | 91 | | 46 | 45 | 20536 | 20878980 | 2011 | Affymetrix Human Genome U133 Plus 2.0 Array |
| Lung cancer | GSE31210 | 246 | 246 | | 226 | 20 | 20536 | 22080568 | 2012 | Affymetrix Human Genome U133 Plus 2.0 Array |
| Lung cancer | GSE32863 | 116 | 87 | | 33 | 54 | 19305 | 22613842 | 2012 | Illumina HumanWG-6 v3.0 expression beadchip |
| Lung cancer | GSE40791 | 194 | 194 | | 94 | 100 | 20536 | 23187126 | 2012 | Affymetrix Human Genome U133 Plus 2.0 Array |
| Lung cancer | GSE27262 | 50 | 50 | | 25 | 25 | 20536 | 22726390 | 2012 | Affymetrix Human Genome U133 Plus 2.0 Array |
| Lung cancer | GSE30219 | 307 | 307 | | 293 | 14 | 20536 | 23698379 | 2013 | Affymetrix Human Genome U133 Plus 2.0 Array |
| Lung cancer | GSE32665 | 179 | 170 | | 81 | 89 | 19030 | 23591868 | 2013 | Illumina human-6 v2.0 expression beadchip |
| Lung cancer | GSE43458 | 110 | 110 | | 80 | 30 | 18720 | 23659968 | 2013 | Affymetrix Human Gene 1.0 ST Array |
| Lung cancer | GSE63459 | 65 | 65 | | 33 | 32 | 17637 | 26134223 | 2015 | Illumina HumanRef-8 v3.0 expression beadchip |
| Lung cancer | GSE75037 | 166 | 134 | | 51 | 83 | 19305 | 27354471 | 2016 | Illumina HumanWG-6 v3.0 expression beadchip |
| Lung cancer | GSE31552 | 131 | 102 | | 51 | 51 | 18720 | NA | NA | Affymetrix Human Gene 1.0 ST Array |
| Lung cancer | GSE33532 | 100 | 40 | | 20 | 20 | 20536 | NA | NA | Affymetrix Human Genome U133 Plus 2.0 Array |
| Lung cancer | GSE40275 | 84 | 72 | | 29 | 43 | 20923 | NA | NA | Human Exon 1.0 ST Array |
| Melanoma | GSE7553 | 87 | 19 | | 14 | 5 | 20536 | 18442402 | 2008 | Affymetrix Human Genome U133 Plus 2.0 Array |
| Melanoma | GSE15605 | 74 | 60 | | 44 | 16 | 20536 | 23633021 | 2013 | Affymetrix Human Genome U133 Plus 2.0 Array |
| Melanoma | GSE38312 | 10 | 10 | | 5 | 5 | 20536 | 24011128 | 2014 | Affymetrix Human Genome U133 Plus 2.0 Array |
| Melanoma | GSE57715 | 297 | 297 | | 280 | 17 | 17637 | NA | NA | Illumina HumanRef-8 WG-DASL v3.0 |
| Ovarian cáncer | GSE10971 | 37 | 37 | | 13 | 24 | 20536 | 18593983 | 2008 | Affymetrix Human Genome U133 Plus 2.0 Array |
| Ovarian cáncer | GSE14407 | 24 | 19 | | 7 | 12 | 20536 | 20040092 | 2009 | Affymetrix Human Genome U133 Plus 2.0 Array |
| Ovarian cáncer | GSE18520 | 63 | 63 | | 53 | 10 | 20536 | 19962670 | 2009 | Affymetrix Human Genome U133 Plus 2.0 Array |
| Ovarian cáncer | GSE19352 | 28 | 22 | | 20 | 2 | 20536 | 20179205 | 2010 | Affymetrix Human Genome U133 Plus 2.0 Array |
| Ovarian cáncer | GSE36668 | 12 | 12 | | 8 | 4 | 20536 | 23029477 | 2012 | Affymetrix Human Genome U133 Plus 2.0 Array |
| Ovarian cáncer | GSE40595 | 77 | 75 | | 61 | 14 | 20536 | 23824740 | 2013 | Affymetrix Human Genome U133 Plus 2.0 Array |
| Ovarian cáncer | GSE69428 | 29 | 17 | | 8 | 9 | 20536 | 26415052 | 2016 | Affymetrix Human Genome U133 Plus 2.0 Array |
| Pancreatic cancer | GSE15471 | 78 | 72 | | 36 | 36 | 20503 | 19260470 | 2008 | Affymetrix Human Genome U133 Plus 2.0 Array |
| Pancreatic cancer | GSE19650 | 22 | 19 | | 12 | 7 | 20503 | 20955708 | 2011 | Affymetrix Human Genome U133 Plus 2.0 Array |
| Pancreatic cancer | GSE32676 | 32 | 32 | | 25 | 7 | 20503 | 22261810 | 2012 | Affymetrix Human Genome U133 Plus 2.0 Array |
| Pancreatic cancer | GSE41368 | 12 | 12 | | 6 | 6 | 18994 | 24120476 | 2014 | Affymetrix Human Gene 1.0 ST Array |
| Pancreatic cancer | GSE43795 | 31 | 11 | | 6 | 5 | 21026 | 24072181 | 2014 | Illumina HumanHT-12 V4.0 expression beadchip |
| Pancreatic cancer | GSE55643 | 53 | 21 | | 18 | 3 | 19596 | 25415223 | 2014 | Agilent-014850 Whole Human Genome Microarray 4x44K G4112F |
| Pancreatic cancer | GSE62452 | 130 | 130 | | 69 | 61 | 18994 | 27197190 | 2016 | Affymetrix Human Gene 1.0 ST Array |
| Pancreatic cancer | GSE62165 | 131 | 131 | | 118 | 13 | 18553 | 27520560 | 2016 | Affymetrix Human Genome U219 Array |
| Pancreatic cancer | GSE63111 | 35 | 35 | | 28 | 7 | 22870 | 28592875 | 2017 | Affymetrix Human Exon 1.0 ST Array |
| Pancreatic cancer | GSE22780 | 16 | 14 | | 8 | 6 | 20503 | NA | NA | Affymetrix Human Genome U133 Plus 2.0 Array |
| Prostate cancer | GSE17951 | 154 | 122 | | 109 | 13 | 20536 | 20663908 | 2010 | Affymetrix Human Genome U133 Plus 2.0 Array |
| Prostate cancer | GSE21034 | 370 | 160 | | 131 | 29 | 20923 | 20579941 | 2010 | Affymetrix Human Exon 1.0 ST Array |
| Prostate cancer | GSE29079 | 95 | 95 | | 47 | 48 | 20923 | 22142399 | 2011 | Affymetrix Human Exon 1.0 ST Array |
| Prostate cancer | GSE32571 | 98 | 98 | | 59 | 39 | 19259 | 22945237 | 2013 | Illumina HumanHT-12 V3.0 expression beadchip |
| Prostate cancer | GSE46602 | 50 | 50 | | 36 | 14 | 20536 | 26522007 | 2015 | Affymetrix Human Genome U133 Plus 2.0 Array |
| Prostate cancer | GSE62872 | 424 | 424 | | 264 | 160 | 18720 | 25371445 | 2015 | Affymetrix Human Gene 1.0 ST Array |
| Prostate cancer | GSE70768 | 199 | 199 | | 125 | 74 | 21042 | 26501111 | 2015 | Illumina HumanHT-12 V4.0 expression beadchip |
| Prostate cancer | GSE71016 | 95 | 91 | | 45 | 46 | 21755 | 27578002 | 2016 | Agilent-039494 SurePrint G3 Human GE v2 8x60K Microarray 039381 |
| Prostate cancer | GSE72220 | 147 | 72 | | 25 | 47 | 20923 | 26945428 | 2016 | Affymetrix Human Exon 1.0 ST Array |
| Prostate cancer | GSE7307 | 677 | 23 | | 10 | 13 | 20536 | NA | NA | Affymetrix Human Genome U133 Plus 2.0 Array |
| Prostate cancer | GSE94767 | 241 | 162 | | 129 | 33 | 20923 | NA | NA | Affymetrix Human Exon 1.0 ST Array |
| Thyroid cancer | GSE3467 | 18 | 18 | | 9 | 9 | 20536 | 16365291 | 2005 | Affymetrix Human Genome U133 Plus 2.0 Array |
| Thyroid cancer | GSE53157 | 27 | 27 | | 24 | 3 | 20536 | 19809427 | 2009 | Affymetrix Human Genome U133 Plus 2.0 Array |
| Thyroid cancer | GSE3678 | 14 | 14 | | 7 | 7 | 20536 | 19380787 | 2009 | Affymetrix Human Genome U133 Plus 2.0 Array |
| Thyroid cancer | GSE33630 | 105 | 105 | | 17 | 3 | 20536 | 22266856 | 2012 | Affymetrix Human Genome U133 Plus 2.0 Array |
| Thyroid cancer | GSE60542 | 92 | 78 | | 48 | 30 | 20536 | 25965298 | 2015 | Affymetrix Human Genome U133 Plus 2.0 Array |
| Thyroid cancer | GSE65144 | 25 | 25 | | 12 | 13 | 20536 | 25675381 | 2015 | Affymetrix Human Genome U133 Plus 2.0 Array |
| Thyroid cancer | GSE35570 | 116 | 116 | | 65 | 51 | 20536 | 26810418 | 2016 | Affymetrix Human Genome U133 Plus 2.0 Array |
| Thyroid cancer | GSE29265 | 49 | 49 | | 29 | 20 | 20536 | NA | NA | Affymetrix Human Genome U133 Plus 2.0 Array |
| Ulcerative colitis | GSE75214 | 119 | 118 | | 96 | 22 | 18994 | 28885228 | 2017 | Affymetrix Human Gene 1.0 ST Array |
| Ulcerative colitis | GSE73661 | 79 | 79 | | 67 | 12 | 18994 | 27802155 | 2018 | Affymetrix Human Gene 1.0 ST Array |
| Ulcerative colitis | GSE48634 | 137 | 137 | | 68 | 69 | 21026 | 25171508 | 2014 | Illumina HumanHT-12 V4.0 expression beadchip |
| Ulcerative colitis | GSE16879 | 30 | 30 | | 24 | 6 | 20536 | 19956723 | 2009 | Affymetrix Human Genome U133 Plus 2.0 Array |
| Ulcerative colitis | GSE59071 | 108 | 107 | | 96 | 11 | 18994 | 26313692 | 2015 | Affymetrix Human Gene 1.0 ST Array |
| Ulcerative colitis | GSE97012 | 49 | 47 | | 22 | 25 | 18347 | 28806280 | 2017 | Agilent-028004 SurePrint G3 Human GE 8x60K Microarray |
| Ulcerative colitis | GSE10616 | 21 | 21 | | 10 | 11 | 20503 | 18758464 | 2008 | Affymetrix GeneChip Human Genome U133 Plus 2.0 Array |
| Ulcerative colitis | GSE13367 | 43 | 26 | | 16 | 10 | 20503 | 19834973 | 2010 | Affymetrix Human Genome U133 Plus 2.0 Array |
| Ulcerative colitis | GSE48958 | 21 | 21 | | 13 | 8 | 18994 | 25546151 | 2014 | Affymetrix Human Gene 1.0 ST Array |
| Ulcerative colitis | GSE38713 | 43 | 43 | | 30 | 15 | 20503 | 23135761 | 2013 | Affymetrix Human Genome U133 Plus 2.0 Array |
| Malaria | GSE34404 | 155 | 154 | | 93 | 61 | 21026 | 22949651 | 2012 | Illumina HumanHT-12 V4.0 expression beadchip |
| Malaria | GSE5418 | 70 | 69 | | 47 | 22 | 12436 | 23135761 | 2013 | Affymetrix Human Genome U133 Plus 2.0 Array |
| Malaria | GSE117613 | 46 | 46 | | 34 | 12 | 21026 | 30060095 | 2018 | Illumina HumanHT-12 V4.0 expression beadchip |

**Table S1: Datasets included in each differential gene expression meta-analysis. The initial samples column show the original number of samples included in the dataset. The final after quality control column show the number of samples available after application of inclusion criteria and mean IAC based outlier samples removal.**

**Table S2:**

| Covariate |  | ASD | Control | p-value |
| --- | --- | --- | --- | --- |
| 1. After control samples addition | | | | |
| GSE28521 + GSE36192 | |  |  |  |
| Gender | **Female** | **4 (25%)** | **11 (20%)** | **0.90 (Chi^2)** |
|  | **Male** | **12 (75%)** | **45 (80%)** |  |
| Age | **Mean** | **24.56** | **27.26** | **0.47 (t.test)** |
|  | **sd** | **13.75** | **10.69** |  |
| PMI | **Mean** | **19.87** | **23.47** | **0.28 (t.test)** |
|  | **Sd** | **8.6** | **18.47** |  |
| GSUPTA |  |  |  |  |
| Gender | **Female** | **4 (17%)** | **3 (16%)** | **1 (Chi^2)** |
|  | **Male** | **19 (83%)** | **16 (84%)** |  |
| Age | **Mean** | **22.17** | **14.42** | **0.06 (t.test)** |
|  | **sd** | **18.37** | **6.18** |  |
| PMI | **Mean** | **23** | **15.76** | **0.01 (t.test)** |
|  | **Sd** | **10.84** | **6.61** |  |
| GSE28475 + GSE36192 | |  |  |  |
| Gender | **Female** | **5 (22%)** | **17 (26%)** | **0.91 (Chi^2)** |
|  | **Male** | **18 (78%)** | **49 (74%)** |  |
| Age | **Mean** | **18.47** | **18.5** | **0.99 (t.test)** |
|  | **sd** | **15.68** | **10** |  |
| PMI | **Mean** | **20.21** | **18.78** | **0.58 (t.test)** |
| 1. After redundant samples removal | | | | |
| GSE28521 + GSE36192 | |  |  |  |
| Gender | **Female** | **4 (25%)** | **11 (20%)** | **0.90 (Chi^2)** |
|  | **Male** | **12 (75%)** | **45 (80%)** |  |
| Age | **Mean** | **24.56** | **27.26** | **0.47 (t.test)** |
|  | **sd** | **13.75** | **10.69** |  |
| PMI | **Mean** | **19.87** | **23.43** | **0.28 (t.test)** |
|  | **Sd** | **8.6** | **18.47** |  |
| GUPTA |  |  |  |  |
| Gender | **Female** | **3 (17%)** | **3 (16%)** | **0.79 (Chi^2)** |
|  | **Male** | **7 (83%)** | **14 (84%)** |  |
| Age | **Mean** | **23.2** | **13.8** | **0.23 (t.test)** |
|  | **sd** | **22.9** | **6.21** |  |
| PMI | **Mean** | **24** | **15** | **0.06** |
|  | **Sd** | **13** | **6** |  |
| GSE28475 + GSE36192 | |  |  |  |
| Gender | **Female** | **2 (25%)** | **16 (28%)** | **1 (Chi^2)** |
|  | **Male** | **6 (75%)** | **41 (72%)** |  |
| Age | **Mean** | **13.37** | **18.67** | **0.31 (t.test)** |
|  | **sd** | **13.3** | **9.49** |  |
| PMI | **Mean** | **10.97** | **18.1** | **0.88 (t.test)** |
|  | **Sd** | **10.32** | **11.81** |  |
| 1. Overall data | | | | |
| Gender | **Female** |  |  | **0.85 (Chi^2)** |
|  | **Male** |  |  |  |
| Age | **Mean** |  |  | **0.94 (t test)** |
|  | **sd** |  |  |  |
| PMI | **Mean** |  |  | **0.66 (t test)** |

**Table S2: Distribution of Gender, Age and PMI in cases and control for the 3 ASD datasets after the different pre-processing steps. 1) After control samples addition. 2) After duplicated patient removal. 3) Overall distribution. Significance for differences in gender proportions was assessed by chi squared tests whereas AGE and PMI mean differences between cases and controls were compared using t tests.**

**Table S3:**

| Sample ID | pCh_Age | pCh_Gender | pCh_PMI | pCh_Individual | pCh_Diagnosis | Dataset |
| --- | --- | --- | --- | --- | --- | --- |
| 4936551012_A | 29 | M | 43.25 | AN08166 | Autism | GSE28521/GSE36192 |
| 4936551012_D | 19 | M | 18.58 | AN03217 | Control | GSE28521/GSE36192 |
| 4936551003_A | 39 | M | 13.95 | AN06420 | Autism | GSE28521/GSE36192 |
| 4936551003_D | 56 | M | 24.17 | AN01125 | Control | GSE28521/GSE36192 |
| 4936551011_A | 49 | F | 16.33 | AN17777 | Autism | GSE28521/GSE36192 |
| 4936551011_H | 36 | M | 21.46 | AN10028 | Control | GSE28521/GSE36192 |
| 4936551002_D | 31 | M | 32.92 | AN12137 | Control | GSE28521/GSE36192 |
| 4936551013_A | 51 | M | 22.16 | AN17254 | Autism | GSE28521/GSE36192 |
| 4936551013_D | 22 | M | 21.47 | AN10833 | Control | GSE28521/GSE36192 |
| 4936551013_G | 22 | M | 25 | AN09730 | Autism | GSE28521/GSE36192 |
| 4936551008_A | 5 | M | 25.5 | AN08873 | Autism | GSE28521/GSE36192 |
| 4936551008_H | 21 | M | 29.91 | AN07176 | Control | GSE28521/GSE36192 |
| 4936551010_D | 41 | M | 27.17 | AN01410 | Control | GSE28521/GSE36192 |
| 4936551010_G | 8 | M | 22.16 | AN19511 | Autism | GSE28521/GSE36192 |
| 4936551005_A | 20 | M | 23.66 | AN00764 | Autism | GSE28521/GSE36192 |
| 4936551005_D | 32 | F | 28.92 | AN15566 | Control | GSE28521/GSE36192 |
| 4936551031_A | 16 | M | NA | AN17138 | Autism | GSE28521/GSE36192 |
| 4936551031_D | 51 | M | 4.75 | AN12240 | Control | GSE28521/GSE36192 |
| 4936551007_A | 9 | M | 27 | AN16641 | Autism | GSE28521/GSE36192 |
| 4936551007_D | 24 | M | 21.33 | AN14757 | Control | GSE28521/GSE36192 |
| 4936551006_A | 11 | F | 12.88 | AN16115 | Autism | GSE28521/GSE36192 |
| 4936551006_D | 16 | M | 26.16 | AN17425 | Control | GSE28521/GSE36192 |
| 4936551006_G | 30 | M | 16.06 | AN11989 | Autism | GSE28521/GSE36192 |
| 4936551015_A | 29 | F | 17.83 | AN12457 | Autism | GSE28521/GSE36192 |
| 4936551015_D | 50 | M | 20.4 | AN19442 | Control | GSE28521/GSE36192 |
| 4936551015_G | 27 | M | 8.3 | AN00493 | Autism | GSE28521/GSE36192 |
| 4936551015_H | 44 | M | 23.26 | AN04479 | Control | GSE28521/GSE36192 |
| 4936551004_A | 18 | F | 6.75 | AN01570 | Autism | GSE28521/GSE36192 |
| 4936551004_D | 28 | M | 23.25 | AN19760 | Control | GSE28521/GSE36192 |
| 4936551004_H | 17 | M | 28.92 | AN00544 | Control | GSE28521/GSE36192 |
| 4936551014_A | 30 | M | 20.33 | AN08792 | Autism | GSE28521/GSE36192 |
| 4936551014_D | 44 | M | 24.61 | AN00142 | Control | GSE28521/GSE36192 |
| UMARY-1347-FCTX | 19 | F | 16 | UMARY-1347-FCTX | Control | GSE28521/GSE36192 |
| UMARY-650-FCTX | 25 | F | 16 | UMARY-650-FCTX | Control | GSE28521/GSE36192 |
| UMARY-1185-FCTX | 4 | M | 17 | UMARY-1185-FCTX | Control | GSE28521/GSE36192 |
| 036-08-FCTX | 20 | F | 40 | 036-08-FCTX | Control | GSE28521/GSE36192 |
| 018-09-FCTX | 57 | M | 49 | 018-09-FCTX | Control | GSE28521/GSE36192 |
| UMARY-1792-FCTX | 25 | F | 11 | UMARY-1792-FCTX | Control | GSE28521/GSE36192 |
| UMARY-5087-FCTX | 44 | M | 4 | UMARY-5087-FCTX | Control | GSE28521/GSE36192 |
| 006-09-FCTX | 25 | M | 81 | 006-09-FCTX | Control | GSE28521/GSE36192 |
| UMARY-4540-FCTX | 25 | M | 23 | UMARY-4540-FCTX | Control | GSE28521/GSE36192 |
| 007-09-FCTX | 25 | M | 81 | 007-09-FCTX | Control | GSE28521/GSE36192 |
| UMARY-4729-FCTX | 24 | M | 10 | UMARY-4729-FCTX | Control | GSE28521/GSE36192 |
| UMARY-818-FCTX | 27 | M | 10 | UMARY-818-FCTX | Control | GSE28521/GSE36192 |
| UMARY-1583-FCTX | 26 | M | 18 | UMARY-1583-FCTX | Control | GSE28521/GSE36192 |
| UMARY-4726-FCTX | 28 | M | 6 | UMARY-4726-FCTX | Control | GSE28521/GSE36192 |
| UMARY-1038-FCTX | 24 | F | 7 | UMARY-1038-FCTX | Control | GSE28521/GSE36192 |
| UMARY-1259-FCTX | 24 | M | 8 | UMARY-1259-FCTX | Control | GSE28521/GSE36192 |
| UMARY-1713-FCTX | 23 | M | 8 | UMARY-1713-FCTX | Control | GSE28521/GSE36192 |
| 014-09-FCTX | 26 | M | 44 | 014-09-FCTX | Control | GSE28521/GSE36192 |
| 023-08-FCTX | 24 | F | 47 | 023-08-FCTX | Control | GSE28521/GSE36192 |
| UMARY-1114-FCTX | 31 | M | 15 | UMARY-1114-FCTX | Control | GSE28521/GSE36192 |
| UMARY-5125-FCTX | 24 | F | 9 | UMARY-5125-FCTX | Control | GSE28521/GSE36192 |
| UMARY-1442-FCTX | 22 | M | 7 | UMARY-1442-FCTX | Control | GSE28521/GSE36192 |
| UMARY-1226-FCTX | 23 | M | 21 | UMARY-1226-FCTX | Control | GSE28521/GSE36192 |
| UMARY-4722-FCTX | 14 | M | 16 | UMARY-4722-FCTX | Control | GSE28521/GSE36192 |
| UMARY-5085-FCTX | 38 | M | 26 | UMARY-5085-FCTX | Control | GSE28521/GSE36192 |
| UMARY-1455-FCTX | 25 | F | 7 | UMARY-1455-FCTX | Control | GSE28521/GSE36192 |
| UMARY-1500-FCTX | 6 | M | 18 | UMARY-1500-FCTX | Control | GSE28521/GSE36192 |
| 033-08-FCTX | 22 | M | 95 | 033-08-FCTX | Control | GSE28521/GSE36192 |
| UMARY-1403-FCTX | 21 | M | 19 | UMARY-1403-FCTX | Control | GSE28521/GSE36192 |
| UMARY-933-FCTX | 20 | M | 12 | UMARY-933-FCTX | Control | GSE28521/GSE36192 |
| 020-07-FCTX | 22 | F | 45 | 020-07-FCTX | Control | GSE28521/GSE36192 |
| UMARY-1794-FCTX | 21 | M | 17 | UMARY-1794-FCTX | Control | GSE28521/GSE36192 |
| UMARY-1104-FCTX | 35 | M | 12 | UMARY-1104-FCTX | Control | GSE28521/GSE36192 |
| UMARY-1668-FCTX | 19 | M | 24 | UMARY-1668-FCTX | Control | GSE28521/GSE36192 |
| UMARY-602-FCTX | 27 | M | 15 | UMARY-602-FCTX | Control | GSE28521/GSE36192 |
| UMARY-257-FCTX | 25 | F | 32 | UMARY-257-FCTX | Control | GSE28521/GSE36192 |
| UMARY-4786-FCTX | 22 | M | 11 | UMARY-4786-FCTX | Control | GSE28521/GSE36192 |
| UMARY-1027-FCTX | 22 | M | 9 | UMARY-1027-FCTX | Control | GSE28521/GSE36192 |
| UMARY-4543-FCTX | 28 | M | 13 | UMARY-4543-FCTX | Control | GSE28521/GSE36192 |
| UMARY-5079-FCTX | 33 | M | 16 | UMARY-5079-FCTX | Control | GSE28521/GSE36192 |
| ba10_s14 | 2 | M | 4.00 | AN03345 | Autism | Gupta |
| ba10_s23 | 5 | F | 33.00 | AN13872 | Autism | Gupta |
| ba10_s69 | 18 | F | 8.00 | UMB1571 | Control | Gupta |
| ba10_s71 | 20 | M | 8.00 | UMB1712 | Control | Gupta |
| ba10_s72 | 13 | M | 18.00 | UMB1790 | Control | Gupta |
| ba10_s73 | 16 | M | 16.00 | UMB1796 | Control | Gupta |
| ba10_s74 | 15 | M | 18.00 | UMB1823 | Control | Gupta |
| ba10_s75 | 19 | M | 14.00 | UMB1841 | Control | Gupta |
| ba10_s79 | 16 | F | 20.00 | UMB1944 | Control | Gupta |
| ba10_s85 | 17 | M | 23.00 | UMB4728 | Control | Gupta |
| ba10_s86 | 56 | M | 19.00 | AN01093 | Autism | Gupta |
| ba44_s27 | 49 | F | 21.08 | AN03632 | Autism | Gupta |
| ba44_s28 | 60 | M | 26.50 | AN09714 | Autism | Gupta |
| ba44_s39 | 22 | M | 24.20 | AN14368 | Control | Gupta |
| ba44_s44 | 5 | M | 39.00 | UMB1349 | Autism | Gupta |
| ba44_s45 | 20 | F | 50.00 | UMB1638 | Autism | Gupta |
| ba44_s47 | 8 | M | 16.00 | UMB4721 | Autism | Gupta |
| ba44_s48 | 7 | M | 20.00 | UMB4849 | Autism | Gupta |
| ba44_s50 | 20 | M | 14.00 | UMB4999 | Autism | Gupta |
| ba44_s52 | 4 | M | 15.00 | UMB451 | Control | Gupta |
| ba44_s55 | 4 | M | 17.00 | UMB1185 | Control | Gupta |
| ba44_s56 | 5 | F | 20.00 | UMB1377 | Control | Gupta |
| ba44_s58 | 8 | M | NA | UMB1674 | Control | Gupta |
| ba44_s60 | 4 | M | 17.00 | UMB4670 | Control | Gupta |
| ba44_s63 | 18 | M | 6.00 | UMB1409 | Control | Gupta |
| ba44_s64 | 18 | M | 9.00 | UMB1429 | Control | Gupta |
| ba44_s65 | 17 | M | 4.00 | UMB1465 | Control | Gupta |
| UMARY-455-FCTX | 19 | M | 13 | UMARY-455-FCTX | Control | GSE28475/GSE36192 |
| UMARY-72-FCTX | 18 | F | 29 | UMARY-72-FCTX | Control | GSE28475/GSE36192 |
| UMARY-1611-FCTX | 18 | M | 11 | UMARY-1611-FCTX | Control | GSE28475/GSE36192 |
| UMARY-4590-FCTX | 20 | M | 19 | UMARY-4590-FCTX | Control | GSE28475/GSE36192 |
| UMARY-305-FCTX | 17 | M | 12 | UMARY-305-FCTX | Control | GSE28475/GSE36192 |
| UMARY-433-FCTX | 19 | M | 31 | UMARY-433-FCTX | Control | GSE28475/GSE36192 |
| UMARY-879-FCTX | 21 | M | 13 | UMARY-879-FCTX | Control | GSE28475/GSE36192 |
| UMARY-4782-FCTX | 18 | M | 17 | UMARY-4782-FCTX | Control | GSE28475/GSE36192 |
| MIAMI-3772-FCTX | 48 | M | 22 | MIAMI-3772-FCTX | Control | GSE28475/GSE36192 |
| UMARY-1672-FCTX | 19 | M | 28 | UMARY-1672-FCTX | Control | GSE28475/GSE36192 |
| UMARY-1612-FCTX | 19 | F | 24 | UMARY-1612-FCTX | Control | GSE28475/GSE36192 |
| UMARY-630-FCTX | 19 | M | 25 | UMARY-630-FCTX | Control | GSE28475/GSE36192 |
| UMARY-1841-FCTX | 19 | M | 14 | UMARY-1841-FCTX | Control | GSE28475/GSE36192 |
| UMARY-4669-FCTX | 16 | M | 16 | UMARY-4669-FCTX | Control | GSE28475/GSE36192 |
| 012-09-FCTX | 17 | M | 89 | 012-09-FCTX | Control | GSE28475/GSE36192 |
| UMARY-1076-FCTX | 17 | M | 19 | UMARY-1076-FCTX | Control | GSE28475/GSE36192 |
| UMARY-164-FCTX | 17 | M | 16 | UMARY-164-FCTX | Control | GSE28475/GSE36192 |
| UMARY-1571-FCTX | 18 | F | 8 | UMARY-1571-FCTX | Control | GSE28475/GSE36192 |
| UMARY-26-FCTX | 18 | F | 16 | UMARY-26-FCTX | Control | GSE28475/GSE36192 |
| UMARY-1712-FCTX | 20 | M | 8 | UMARY-1712-FCTX | Control | GSE28475/GSE36192 |
| UMARY-1475-FCTX | 20 | M | 3 | UMARY-1475-FCTX | Control | GSE28475/GSE36192 |
| UMARY-1279-FCTX | 17 | F | 19 | UMARY-1279-FCTX | Control | GSE28475/GSE36192 |
| UMARY-1465-FCTX | 17 | M | 4 | UMARY-1465-FCTX | Control | GSE28475/GSE36192 |
| UMARY-4727-FCTX | 20 | M | 5 | UMARY-4727-FCTX | Control | GSE28475/GSE36192 |
| UMARY-165-FCTX | 15 | M | 20 | UMARY-165-FCTX | Control | GSE28475/GSE36192 |
| UMARY-4724-FCTX | 16 | F | 15 | UMARY-4724-FCTX | Control | GSE28475/GSE36192 |
| UMARY-1790-FCTX | 13 | M | 18 | UMARY-1790-FCTX | Control | GSE28475/GSE36192 |
| UMARY-4591-FCTX | 16 | F | 14 | UMARY-4591-FCTX | Control | GSE28475/GSE36192 |
| UMARY-5077-FCTX | 16 | F | 13 | UMARY-5077-FCTX | Control | GSE28475/GSE36192 |
| UMARY-1607-FCTX | 17 | F | 24 | UMARY-1607-FCTX | Control | GSE28475/GSE36192 |
| UMARY-1862-FCTX | 20 | M | 6 | UMARY-1862-FCTX | Control | GSE28475/GSE36192 |
| UMARY-1078-FCTX | 17 | F | 12 | UMARY-1078-FCTX | Control | GSE28475/GSE36192 |
| UMARY-1105-FCTX | 16 | M | 17 | UMARY-1105-FCTX | Control | GSE28475/GSE36192 |
| UMARY-4916-FCTX | 19 | M | 5 | UMARY-4916-FCTX | Control | GSE28475/GSE36192 |
| UMARY-1013-FCTX | 18 | M | 27 | UMARY-1013-FCTX | Control | GSE28475/GSE36192 |
| UMARY-4848-FCTX | 16 | M | 15 | UMARY-4848-FCTX | Control | GSE28475/GSE36192 |
| UMARY-1845-FCTX | 18 | F | 29 | UMARY-1845-FCTX | Control | GSE28475/GSE36192 |
| UMARY-1037-FCTX | 19 | M | 11 | UMARY-1037-FCTX | Control | GSE28475/GSE36192 |
| UMARY-1429-FCTX | 18 | M | 9 | UMARY-1429-FCTX | Control | GSE28475/GSE36192 |
| UMARY-1322-FCTX | 16 | M | 25 | UMARY-1322-FCTX | Control | GSE28475/GSE36192 |
| GSM703645/GSM703650 | 45 | M | 23 | UMB1445 | Autism | GSE28475/GSE36192 |
| GSM703648 | 10 | M | 24 | UMB1650 | Control | GSE28475/GSE36192 |
| GSM703651/GSM703658 | 27 | M | 10 | UMB818 | Control | GSE28475/GSE36192 |
| GSM703652 | 6 | M | 19 | UMB1500 | Control | GSE28475/GSE36192 |
| GSM703653 | 9 | F | 20 | UMB1407 | Control | GSE28475/GSE36192 |
| GSM703654 | 20 | M | 22 | UMB1649 | Control | GSE28475/GSE36192 |
| GSM703655 | 4 | F | 21 | UMB1499 | Control | GSE28475/GSE36192 |
| GSM703656 | 7 | M | 12 | UMB4898 | Control | GSE28475/GSE36192 |
| GSM703657 | 4 | F | 13 | UMB4671 | Autism | GSE28475/GSE36192 |
| GSM703663 | 15 | F | 5 | UMB4638 | Control | GSE28475/GSE36192 |
| GSM703664 | 25 | F | 26 | BTB3960 | Control | GSE28475/GSE36192 |
| GSM703667/GSM703674 | 56 | M | 22 | AN13295 | Control | GSE28475/GSE36192 |
| GSM703668 | 14 | M | 9 | UMB4899 | Autism | GSE28475/GSE36192 |
| GSM703669 | 5 | M | 42.8 | AN13364 | Autism | GSE28475/GSE36192 |
| GSM703672 | 12 | M | 22 | UMB1714 | Control | GSE28475/GSE36192 |
| GSM703673 | 12 | M | 15 | UMB4787 | Control | GSE28475/GSE36192 |
| GSM703679 | 14 | M | 16 | UMB4722 | Control | GSE28475/GSE36192 |
| GSM703686 | 56 | M | 23 | AN10606 | Control | GSE28475/GSE36192 |
| GSM703687 | 15 | M | 23 | AN04682 | Autism | GSE28475/GSE36192 |
| GSM703692 | 30 | M | 14.83 | AN15622 | Control | GSE28475/GSE36192 |
| GSM703694 | 4 | F | 17 | AN02456 | Control | GSE28475/GSE36192 |
| GSM703699/GSM703700/GSM703703/GSM703704 | 7 | F | 14 | UMB1174 | Autism | GSE28475/GSE36192 |
| GSM703701/GSM703702 | 8 | M | 12 | UMB4231 | Autism | GSE28475/GSE36192 |
| GSM703705/GSM703706 | 16 | M | 22 | AN07591 | Control | GSE28475/GSE36192 |
| GSM703713/GSM703714 | 9 | M | 13 | UMB797 | Autism | GSE28475/GSE36192 |

**Table S3: Sample IDs and Age, Gender and PMI information about the non-redundant set of samples included in the final ASD differential gene expression meta-analysis.**

**Table S4:**

| Genes jointly upregulated in ASD and brain cancer | | | | | | | | | |
| --- | --- | --- | --- | --- | --- | --- | --- | --- | --- |
|  |  |  |  |  |  |  |  |  |  |
| IL18BP | BAZ1A | DPYSL3 | WWTR1 | IRF7 | SLC15A3 | ZC3HAV1 | SLC2A5 | C1orf54 | SERPINH1 |
| KIF20A | SDSL | ANO6 | SZRD1 | ITGA5 | TNFRSF12A | PSMB8 | SLC11A1 | RHBDF2 | SNAP23 |
| NAMPT | C1QTNF1 | EIF4EBP2 | HEYL | RHOB | SCARA3 | ADAMTS9 | PHACTR4 | GSDMD | CCNK |
| TOB1 | MARCH3 | SERPINB1 | CHIC2 | RHOC | MAP3K20 | SELENON | SOX4 | DCAKD | HAP1 |
| EBI3 | CKS2 | ELF1 | GEM | LCP1 | PIM1 | PBXIP1 | SOX9 | SLC8B1 | MPZL1 |
| CDK2 | FAM129A | TXLNA | CYTH4 | LGALS3 | PLAU | PLEKHA4 | SPP1 | COL18A1 | SPOCD1 |
| LHFPL2 | SLC16A10 | EMP1 | SLC39A1 | LPP | PLOD1 | PTPN1 | ZFP36L1 | NPL | NMI |
| MPZL2 | SERPINA3 | EMP3 | GNAI2 | LYN | PLSCR1 | HAMP | STAT3 | TRIM56 | MAPKAPK2 |
| CDKN1A | PLIN2 | ERN1 | CNIH4 | MGP | PLTP | CXCL16 | STC1 | FERMT3 | CD14 |
| SF3B4 | CMKLR1 | EYA2 | SERTAD3 | MGST1 | PODXL | JAM2 | TEAD4 | STK40 | CD163 |
| IFITM3 | CNN3 | F2R | HIST1H1C | MMP9 | RBM47 | RARRES3 | TGIF1 | SRPX | EPSTI1 |
| YAP1 | SLC44A3 | FCER1G | ANXA1 | MOV10 | DDIT4 | RBMS2 | TSPO | TAGLN2 | CHST3 |
| CEBPA | PODN | CHSY1 | HCK | MSN | XAF1 | RNASE2 | TIMP1 | ASCC2 | CD37 |
| CEBPB | COL2A1 | CD93 | HCLS1 | MSX1 | GATAD2A | BCL6 | TIMP3 | ANTXR1 | TP53I3 |
| CIB1 | COL4A1 | MAPRE1 | HDAC1 | MUC1 | FAM46C | RP2 | TLR5 | TUBB6 | CD44 |
| IFI44 | COL4A2 | PALLD | EHD4 | MYC | TOR4A | RPS19 | C1QB | HAVCR2 | PDIA4 |
| IFITM2 | ADM | HEY2 | HMOX1 | MYCL | APPL2 | RXRA | C1QC | SPPL2A | ISG15 |
| RBCK1 | EGFLAM | FPR1 | HSPB1 | MYH9 | HJURP | S100A8 | C5AR1 | PLXDC2 | CD81 |
| TRAF3IP2 | CSRP2 | LY96 | DNAJB1 | NCBP1 | PRCP | S100A9 | UCP2 | AJUBA | STARD8 |
| VAMP5 | DTX3L | RAB38 | IFI16 | NFKB1 | SRGN | S100A10 | UGDH | RHPN2 | MVP |
| MTHFD2 | CTSS | IL17RA | IFI35 | PNP | LYAR | BGN | VEGFA | ITPRIP | IL18BP |
| ALDH1L1 | GLIPR2 | ABL1 | SP110 | DDR2 | RNF114 | SELL | VIM | SCIN | KIF20A |
| HCP5 | CCDC50 | IFI6 | IFRD1 | TNFRSF11B | NXT2 | IFIH1 | PTTG1IP | CAV1 | NAMPT |
| MTMR11 | CYBA | ARHGAP30 | IGFBP5 | OXTR | EIF2AK2 | CSRNP1 | MAPKAPK3 | CASK | TOB1 |
| FERMT2 | RIBC1 | TNFAIP8 | IL4R | SH3GLB1 | B2M | PARP12 | TSEN34 | OASL | EBI3 |
| BTN3A2 | ANKFN1 | BACE2 | CXCL10 | HERC5 | SLC2A4RG | SLA | TCTN1 | VAMP8 | CDK2 |
|  |  |  |  |  |  |  |  |  |  |
| Genes jointly downregulated in ASD and brain cancer | | | | | | | | | |
|  |  |  |  |  |  |  |  |  |  |
| PARP2 | STXBP5 | EFR3A | NSG1 | BEX5 | ANKRD39 | OXR1 | MAGEE1 | TRPC1 | MADD |
| ACTR1B | CRH | FLT3 | INPP5J | IDI1 | PCSK2 | PPP1R7 | PTPN3 | TSSC1 | CADPS |
| FRY | CRHBP | DDHD2 | GLS2 | STAC2 | CEND1 | SLC6A15 | PVALB | TYRP1 | NAPA |
| OPTN | SLC32A1 | MTUS2 | CNTN6 | PLCXD3 | NRN1 | VPS13D | NECTIN1 | VIP | WASF1 |
| RCAN2 | CRYM | RUSC1 | AMPH | HCN1 | PDE1A | DCUN1D2 | RASGRF1 | CACNA1F | RNF8 |
| LANCL1 | FAM81A | SMPX | GLRB | INPP5A | LCMT1 | BBS7 | SARS | CACNB1 | INA |
| NPM2 | ZNF385B | PITPNB | GPI | ARF3 | NCKIPSD | PANK4 | SCN4B | AUNIP | SYNGR3 |
| CAMKK2 | CLVS1 | FLRT3 | ZADH2 | CCDC184 | GSKIP | STRBP | NEUROD6 | NDUFAF5 | KCNAB3 |
| SPINT2 | RHOXF1 | PELI3 | RIMKLA | LRRC75A | RAB6B | ZNF331 | LIN7B | FSD1 | DCLK1 |
| GNB5 | DHCR24 | MTOR | RELL2 | SLC6A17 | GNG13 | PREP | MMS19 | SNRNP25 | B4GALT6 |
| CPLX2 | SCAMP5 | GABRA1 | MAGEH1 | FAM212A | PFKM | SVOP | PJA1 | ZFAND1 | RAB33A |
| MLLT11 | ELAVL4 | TCERG1L | BABAM1 | SERTM1 | PFN2 | OGDHL | CDH22 | PDZD7 | CADPS2 |
| SLC27A2 | LGI3 | GABRG2 | TAGLN3 | PLPP6 | ATP6V1A | PRKCE | WBSCR17 | C10orf88 | CABP1 |
| B4GAT1 | EPB41L1 | GAD2 | DDX25 | MAP3K9 | ATP6V1C1 | DCAF6 | CEP85 | ABTB1 | ENTPD3 |
| MRAP2 | ALAS1 | CNRIP1 | OLA1 | TRIM37 | ATP6V1E1 | BEX1 | REEP1 | USP5 | CARTPT |
| CHRNA4 | ZNF25 | CABYR | FAM216A | NEFH | RBM11 | MAPK9 | SLC6A13 | BHLHB9 | NUP93 |
| MAL2 | CNKSR2 | ANKRD2 | HAGH | ATP2B2 | HEATR5B | MAP2K1 | SLCO1A2 | ITFG1 | GPRASP1 |
| ZBED9 | FKBP3 | LHX6 | HINT1 | ATP2B3 | HMGCLL1 | SLC17A6 | SNCA | TMEM246 | KIAA0513 |
| AP2M1 | WDR47 | SEZ6L2 | PPM1J | OAT | RNF111 | CAMK1G | SST | CUL3 | FIG4 |
| UBR3 | KIFAP3 | NAP1L5 | ICA1 | OPA1 | MFSD6 | LRFN2 | STAT4 | SPRYD3 | IQSEC1 |
| RIPPLY2 | RAB18 | GFRA2 | FAM19A2 | OXCT1 | TTC19 | ARHGAP20 | VAMP1 | UBASH3B | PARP2 |
| COX7B | MPRIP | TRUB2 | KIAA2022 | DYNC1LI1 | RPP25 | MYH7B | TPI1 | SELENOI | ACTR1B |
| HCP5 | CCDC50 | IFI6 | IFRD1 | TNFRSF11B | NXT2 | IFIH1 | PTTG1IP | CAV1 | NAMPT |
| MTMR11 | CYBA | ARHGAP30 | IGFBP5 | OXTR | EIF2AK2 | CSRNP1 | MAPKAPK3 | CASK | TOB1 |
| FERMT2 | RIBC1 | TNFAIP8 | IL4R | SH3GLB1 | B2M | PARP12 | TSEN34 | OASL | EBI3 |
| BTN3A2 | ANKFN1 | BACE2 | CXCL10 | HERC5 | SLC2A4RG | SLA | TCTN1 | VAMP8 | CDK2 |
|  |  |  |  |  |  |  |  |  |  |
| Genes jointly upregulated in ASD and kidney cancer | | | | | | | | | |
|  |  |  |  |  |  |  |  |  |  |
| IL18BP | CMTM7 | ELF1 | HEYL | ING1 | SH3GLB1 | EIF2AK2 | SOX9 | SLC7A5 | MPZL1 |
| KIF20A | C1QTNF1 | TXLNA | CHIC2 | CXCL10 | HERC5 | B2M | STC1 | TRIM56 | NMI |
| NAMPT | MB21D1 | APOBEC3F | GEM | IRF7 | SLC15A3 | PSMB8 | TEAD4 | LZTR1 | CD14 |
| EBI3 | MARCH3 | EMP1 | GJA4 | ITGA5 | TNFRSF12A | PLEKHA4 | TGM2 | HIST1H2AC | CD163 |
| CDK2 | FAM129A | EMP3 | CYTH4 | KIFC1 | PLA1A | PTPN1 | TSPO | SH3BGRL3 | GCNA |
| LHFPL2 | PLIN2 | ERN1 | SLC39A1 | RHOB | ACSL5 | HAMP | TIMP1 | FERMT3 | EPSTI1 |
| MPZL2 | CMKLR1 | F2R | GNAI2 | LASP1 | MAP3K20 | CXCL16 | C1QB | INHBE | CD37 |
| MFSD10 | GIPC3 | FCER1G | SLCO4A1 | LCP1 | PIM1 | RARRES3 | C1QC | TAGLN2 | CD44 |
| CDKN1A | COL4A1 | CHSY1 | CNIH4 | LGALS3 | PLAUR | RBMS2 | TPD52L2 | ASCC2 | PDIA4 |
| IFITM3 | COL4A2 | CD93 | HIST1H1C | LPP | PLOD1 | RNASE2 | C5AR1 | ANTXR1 | ISG15 |
| CEBPA | ADM | HEY2 | ANXA1 | LYN | PLSCR1 | BCL6 | UCP2 | TUBB6 | CD81 |
| CEBPB | EGFLAM | FPR1 | HCK | TM4SF1 | PLTP | RP2 | VEGFA | HAVCR2 | FCHSD2 |
| IFI44 | DTX3L | CARHSP1 | HCLS1 | MDK | DDIT4 | RPS19 | VIM | PLXDC2 | MVP |
| IFITM2 | CTSS | LY96 | EHD4 | MMP9 | PPARG | S100A8 | WAS | HELZ2 | IL18BP |
| RBCK1 | GLIPR2 | PPP1R15A | HMOX1 | MSN | XAF1 | S100A9 | PTTG1IP | ITPRIP | KIF20A |
| TRAF3IP2 | CCDC50 | RAB38 | ICAM2 | MSX1 | TOR4A | S100A10 | C1orf54 | CAV1 | NAMPT |
| VAMP5 | CTSZ | IL17RA | IFI16 | MYC | DUSP23 | BGN | RHBDF2 | CASK | EBI3 |
| MTHFD2 | GPR180 | IFI6 | IFI35 | MYH9 | VPS37C | SELL | KLHL36 | OASL | CDK2 |
| HCP5 | DPYSL3 | ARHGAP30 | SP110 | NFKB1 | HJURP | IFIH1 | GSDMD | PEA15 | LHFPL2 |
| MTMR11 | ANO6 | TNFAIP8 | IFRD1 | NFKB2 | SRGN | PARP12 | ALG9 | SERPINH1 | MPZL2 |
| BTN3A2 | MLKL | WWTR1 | LILRA5 | NOS3 | LYAR | SLA | SLC8B1 | SNAP23 | MFSD10 |
| BAZ1A | SERPINB1 | PITPNC1 | IL4R | NRTN | NXT2 | SLC11A1 | APOL4 | SPHK1 | CDKN1A |
|  |  |  |  |  |  |  |  |  |  |
| Genes jointly downregulated in ASD and kidney cancer | | | | | | | | | |
|  |  |  |  |  |  |  |  |  |  |
| ACOT8 | DNAJC19 | NT5DC1 | NSG1 | INPP5A | OXCT1 | UBL3 | MRPS22 | SNCA | SPRYD3 |
| PARP2 | COX6B1 | OARD1 | INPP5J | ARF3 | ATP5C1 | POLRMT | SDR39U1 | TM7SF2 | MPND |
| RWDD2B | RIPPLY2 | DOLK | GLS2 | IPO5 | COQ6 | HMGCLL1 | NIT2 | TSSC1 | MADD |
| TSFM | COX7B | FKBP3 | AMPH | SLC6A17 | MRPL4 | ASNSD1 | ADCK1 | TYRP1 | CRADD |
| FRY | MUM1L1 | DHX30 | GLRB | SAMD5 | MRPS7 | TTC19 | CAMK1G | UQCRFS1 | NAPA |
| LANCL1 | CRHBP | FLT3 | ZADH2 | SERTM1 | NDUFAF1 | PPL | VPS18 | ZNF165 | PEX11B |
| NPM2 | CRYM | ISCU | MAGEH1 | PLPP6 | DYNC1LI1 | FAM206A | MYH7B | CUEDC2 | BAIAP3 |
| FARS2 | FAM81A | GSPT2 | TAGLN3 | MGST3 | PCSK2 | RMND1 | MAGEE1 | ASPSCR1 | RNF8 |
| GNB5 | RUNDC1 | FLRT3 | DDX25 | MAP3K9 | PDE1A | DET1 | PTPN3 | NDUFAF5 | TMEM169 |
| NOXA1 | C1orf74 | MTOR | GSS | C6orf226 | RTCB | VPS13D | PVALB | CHCHD7 | B4GALT6 |
| USP19 | PDIK1L | C8orf46 | HAGH | NDUFA7 | ATP5F1 | DCUN1D2 | NECTIN1 | LRRC2 | CADPS2 |
| SLC27A2 | ZNF385B | MTO1 | ANXA7 | NDUFA9 | GSKIP | RCBTB1 | RASGRF1 | SNRNP25 | ATG5 |
| B4GAT1 | CYC1 | SUMF2 | PPM1J | NDUFB2 | PDHB | ABCF3 | RPL22 | BEND5 | ENTPD3 |
| KAT7 | DDX1 | ANAPC15 | ICA1 | NDUFB6 | PFKM | STRBP | LIN7B | CCDC51 | RGS6 |
| MRAP2 | DLD | DHRS7B | KIAA2022 | NDUFV1 | PFN2 | ZNF331 | PJA1 | THOC7 | GPRASP1 |
| CHRNA4 | DPYS | ATRNL1 | BEX5 | NEFH | ATP5J | SVOP | WBSCR17 | GRPEL1 | ACOT8 |
| MAL2 | SCAMP5 | DCAF4 | IDH3B | NIT1 | ATP6V1A | OGDHL | MRPS11 | BHLHB9 | PARP2 |
| SLC25A26 | ENDOG | ANKRD2 | IDI1 | ATP2B3 | ATP6V1C1 | PRKCE | MRPS9 | KCNH6 | RWDD2B |
| CLTA | LGI3 | MRPL46 | STAC2 | OAT | ATP6V1E1 | BEX1 | SLC1A1 | SLIRP | TSFM |
| KLHDC9 | EPB41L1 | NAP1L5 | PLCXD3 | OPA1 | ATP5O | PSEN2 | REEP1 | MKKS | FRY |
| COL19A1 | ALAS1 | TRUB2 | ACADSB | ORC4 | RBM11 | PSMB5 | SLC6A13 | POMGNT2 | LANCL1 |
|  |  |  |  |  |  |  |  |  |  |
| Genes jointly upregulated in ASD and thyroid cancer | | | | | | | | | |
|  |  |  |  |  |  |  |  |  |  |
| KIF20A | SLC16A10 | EFNB1 | RAB38 | IL4R | OXTR | ZC3HAV1 | SPP1 | SLC8B1 | PEA15 |
| NAMPT | PLIN2 | ANO6 | ARHGAP30 | CXCL10 | SH3GLB1 | PSMB8 | ZFP36L1 | FOSL1 | SERPINH1 |
| EBI3 | GIPC3 | MLKL | BAMBI | ITGA5 | SLC15A3 | ADAMTS9 | TGM2 | APOL4 | SPHK1 |
| LHFPL2 | CNN3 | SERPINB1 | HEYL | KIFC1 | TNFRSF12A | PLEKHA4 | THBS1 | NPL | SPOCD1 |
| MPZL2 | COL2A1 | TXLNA | GDNF | LASP1 | ACSL5 | CXCL16 | TSPO | SLC7A5 | MAPKAPK2 |
| MFSD10 | COL4A1 | EMP3 | GJA4 | LCP1 | MAP3K20 | RARRES3 | TIMP1 | SH3BGRL3 | CD14 |
| CDKN1A | COL4A2 | AK4 | CYTH4 | LGALS3 | PIK3R2 | RNASE2 | TLR5 | FERMT3 | CD163 |
| YAP1 | ADM | F2R | GLI2 | LYN | PLAU | RP2 | C1QB | INHBE | EPSTI1 |
| RBCK1 | NACC2 | FCER1G | GNAI2 | TM4SF1 | PLAUR | RPS19 | C1QC | TAGLN2 | BAG3 |
| IGF2BP2 | MUC17 | CHSY1 | SFN | MDK | PLOD1 | S100A9 | C5AR1 | TUBB6 | TP53I3 |
| MTHFD2 | ZBTB46 | CD93 | SLCO4A1 | MMP9 | PLTP | S100A10 | UCP2 | HAVCR2 | CD44 |
| HCP5 | DTX3L | ZBTB43 | HCK | MOV10 | DDIT4 | BGN | WAS | PARP10 | ISG15 |
| MTMR11 | CTSS | HEY2 | HCLS1 | MSN | GATAD2A | PARP12 | PTTG1IP | PLXDC2 | FCHSD2 |
| BAZ1A | CYBA | CLCF1 | HMOX1 | MUC1 | TOR4A | SLA | CA12 | HELZ2 | MVP |
| MB21D1 | DPYSL3 | FPR1 | IFI16 | NFKB2 | DUSP23 | SLC2A5 | MAPKAPK3 | SCIN | KIF20A |
| CKS2 | ARID3A | CARHSP1 | IGFBP5 | NOS3 | TMEM51 | SLC11A1 | C1orf54 | OASL | NAMPT |
| FAM129A | HBEGF | LY96 | LILRA5 | PNP | HJURP | SOX4 | RHBDF2 | VAMP8 | EBI3 |
|  |  |  |  |  |  |  |  |  |  |
| Genes jointly downregulated in ASD and thyroid cancer | | | | | | | | | |
|  |  |  |  |  |  |  |  |  |  |
| ACOT8 | FBLN7 | DHX30 | MAGEH1 | PLPP6 | NRN1 | DET1 | LYRM4 | TSSC1 | MADD |
| RWDD2B | UBR3 | MRPS27 | ASTE1 | MGST3 | ATP5F1 | DCUN1D2 | ARHGAP20 | UQCRFS1 | NAPA |
| TSFM | DNAJC19 | DDHD2 | CCDC113 | C6orf226 | RAB6B | RCBTB1 | MYH7B | UROD | PEX11B |
| FRY | COX7B | CSTF2T | DDX25 | NDUFB2 | PFKM | BRF2 | MAGEE1 | VIP | FUBP1 |
| RCAN2 | MUM1L1 | GSPT2 | OLA1 | NDUFB6 | PFN2 | ABCF3 | RPL22 | ZNF18 | EFCAB12 |
| FARS2 | C1orf74 | C8orf46 | FAM216A | NDUFV1 | ATP5J | STRBP | SARS | ZNF225 | SLC25A46 |
| RRAGA | PDIK1L | CCDC96 | ANXA7 | NDUFS4 | PITPNA | ZNF331 | SCN4B | ASPSCR1 | DCLK1 |
| USP19 | ZNF385B | ATXN10 | KIAA2022 | DRG1 | ATP5O | PRKACB | LIN7B | RNF26 | DRC1 |
| B4GAT1 | CYC1 | MTO1 | BEX5 | NIT1 | RBM11 | OGDHL | PJA1 | LRRC2 | CADPS2 |
| KAT7 | DDX1 | ANAPC15 | IDH3B | OAT | PRMT7 | PRKCE | WBSCR17 | SNRNP25 | ATG5 |
| PDCD10 | DLD | DPCD | IDI1 | ORC4 | HMGCLL1 | BEX1 | MRPS11 | BEND5 | CARTPT |
| MRAP2 | DUT | DHRS7B | PLCXD3 | OXCT1 | ASNSD1 | KLHL9 | MRPS9 | CCDC51 | NUP93 |
| EXOC3 | EDN3 | DCAF4 | ACADSB | VILL | RNF111 | SLC25A40 | SLC1A1 | ZFAND1 | GPRASP1 |
| MAL2 | ENDOG | NAP1L5 | INPP5A | SLC35B3 | DCAF16 | MAPK9 | REEP1 | TXNDC15 | SNX17 |
| ZBED9 | LGI3 | GFRA2 | KPNA5 | MRPS18C | TTC19 | PSMB4 | SLC6A13 | NEK11 | IQSEC1 |
| SLC25A26 | ZNF25 | INPP5J | IPO5 | MRPS7 | RPP25 | MRPS22 | SNCA | BHLHB9 | ACOT8 |
| C12orf45 | NT5DC1 | NFU1 | C6orf120 | IFT52 | FAM206A | SDR39U1 | TBCC | ARL6 | RWDD2B |
| KLHDC9 | OARD1 | MMADHC | SAMD5 | NDUFAF1 | PINX1 | SMYD2 | TBCE | TMEM246 | TSFM |
| TMEM18 | ZNF510 | ZADH2 | SERTM1 | RSL24D1 | PIH1D1 | ANKMY2 | TBP | POMGNT2 | FRY |
|  |  |  |  |  |  |  |  |  |  |
| Genes jointly upregulated in ASD and pancreatic cancer | | | | | | | | | |
|  |  |  |  |  |  |  |  |  |  |
| KIF20A | CKS2 | TXLNA | SLCO4A1 | LGALS3 | MAP3K20 | PSMB8 | TGM2 | SH3BGRL3 | CFLAR |
| LHFPL2 | COL4A1 | AK4 | CNIH4 | LPP | PKNOX1 | PBXIP1 | TSPO | FERMT3 | MPZL1 |
| MPZL2 | COL4A2 | EYA2 | SERTAD3 | LYN | PLAU | PTPN1 | TIMP1 | TAGLN2 | SPOCD1 |
| MFSD10 | ADM | F2R | HIST1H1C | TM4SF1 | PLAUR | CXCL16 | C1QB | ASCC2 | NMI |
| MSLN | MUC17 | MAPRE1 | ANXA1 | MDK | PLOD1 | RARRES3 | C1QC | ANTXR1 | MAPKAPK2 |
| SF3B4 | DTX3L | NT5C2 | HDAC1 | MMP9 | PLSCR1 | RBMS2 | TPD52L2 | HAVCR2 | EPSTI1 |
| YAP1 | CTSS | PALLD | ANXA11 | MOV10 | PPARG | RNASE2 | UCP2 | SPPL2A | BAG3 |
| IFI44 | CCDC50 | ZBTB43 | IFI16 | MSN | XAF1 | RP2 | UGDH | PLXDC2 | TP53I3 |
| IFITM2 | CTSZ | RAB38 | IFI35 | MUC1 | GATAD2A | S100A10 | VEGFA | RHPN2 | ISG15 |
| IGF2BP2 | CYBA | IFI6 | SP110 | MYH9 | TOR4A | S100P | PTTG1IP | SCIN | FCHSD2 |
| HCP5 | GPR180 | ARHGAP30 | IGFBP5 | NCBP1 | DUSP23 | BGN | ZFYVE21 | CASK | MVP |
| MTMR11 | ANKFN1 | BACE2 | IL4R | PNP | APPL2 | IFIH1 | C1orf54 | OASL | KIF20A |
| BTN3A2 | DPYSL3 | CHIC2 | CXCL10 | TNFRSF11B | HJURP | PARP12 | APOL4 | VAMP8 | LHFPL2 |
| BAZ1A | HBEGF | GEM | ITGA5 | OXTR | LYAR | SLC11A1 | NPL | PEA15 | MPZL2 |
| CMTM7 | EFNB1 | SLC39A1 | KIFC1 | SLC15A3 | EIF2AK2 | SOX4 | SLC7A5 | SERPINH1 | MFSD10 |
| MB21D1 | MLKL | GLI2 | RHOC | TNFRSF12A | B2M | STAT3 | TRIM56 | SNAP23 | MSLN |
| MARCH3 | ELF1 | SFN | LASP1 | ACSL5 | ZC3HAV1 | TGIF1 | HIST1H2AC | CCNK | SF3B4 |
|  |  |  |  |  |  |  |  |  |  |
| Genes jointly downregulated in ASD and pancreatic cancer | | | | | | | | | |
|  |  |  |  |  |  |  |  |  |  |
| RWDD2B | TMEM18 | SCAMP5 | ATXN10 | OLA1 | RSL24D1 | SLC6A15 | RASGRF1 | FSD1 | BAIAP3 |
| ACTR1B | UBR3 | ELAVL4 | DHRS7B | HAGH | PCSK2 | ZNF331 | SMAP1 | LRRC2 | N4BP2L1 |
| RCAN2 | COL19A1 | SLC10A4 | ATRNL1 | KIAA2022 | CEND1 | SVOP | RPL22 | SNRNP25 | INA |
| NPM2 | RTP1 | SLC29A1 | DCAF4 | BEX5 | CD320 | OGDHL | SARS | BEND5 | KCNAB3 |
| TBL3 | RIPPLY2 | LGI3 | ANKRD2 | PLCXD3 | ANAPC5 | BEX1 | SCN4B | ZFAND1 | CADPS2 |
| FARS2 | STXBP5 | OARD1 | SERGEF | HCN1 | LCMT1 | MAPK9 | CDH22 | TXNDC15 | CABP1 |
| NOXA1 | CRH | DOLK | LHX6 | ACADSB | RAB6B | PSEN2 | WBSCR17 | PDZD7 | ENTPD3 |
| CPLX2 | MUM1L1 | CNKSR2 | SEZ6L2 | IPO5 | GNG13 | SDR39U1 | LMF1 | THOC7 | KIAA0513 |
| USP19 | CRHBP | FLT3 | GFRA2 | SLC6A17 | ATP5J | SLC17A6 | REEP1 | TMEM121 | RWDD2B |
| SLC27A2 | SLC32A1 | MTUS2 | INPP5J | FAM212A | RBM11 | ADCK1 | SLC6A13 | BHLHB9 | ACTR1B |
| B4GAT1 | PDIK1L | C8orf46 | GLS2 | SERTM1 | UBL3 | CAMK1G | SLCO1A2 | KCNH6 | RCAN2 |
| CHRNA4 | ZNF385B | GABRA1 | CNTN6 | PLPP6 | NLE1 | LRFN2 | SST | POMGNT2 | NPM2 |
| SLC22A9 | RHOXF1 | TCERG1L | GLRB | C6orf226 | HMGCLL1 | MYH7B | TM7SF2 | SPRYD3 | TBL3 |
| ZBED9 | DHPS | GABRG2 | GRB10 | DRG1 | TTC19 | MAGEE1 | UQCRFS1 | MPND | FARS2 |
| C12orf45 | DPYS | GAD2 | TAGLN3 | ATP2B2 | PINX1 | PVALB | VIP | CADPS | NOXA1 |
| KLHDC9 | EDN3 | CCDC96 | DDX25 | ATP2B3 | DET1 | NECTIN1 | CACNA1F | CRADD | CPLX2 |
|  |  |  |  |  |  |  |  |  |  |
| Genes jointly upregulated in ASD and downregulated in lung cancer | | | | | | | | | |
|  |  |  |  |  |  |  |  |  |  |
| IL18BP | FAM129A | HBEGF | HEY2 | HBD | MYC | RAVER2 | S100A10 | WAS | PLXDC2 |
| NAMPT | SERPINA3 | EFNB1 | FPR1 | HCK | GADD45B | C4orf19 | SELL | MAPKAPK3 | AJUBA |
| LHFPL2 | PLIN2 | ANO6 | CARHSP1 | HCLS1 | MYH9 | PRCP | CSRNP1 | ZFYVE21 | ITPRIP |
| TRIB1 | CMKLR1 | MLKL | PPP1R15A | EHD4 | NFKB1 | SRGN | SLA | C1orf54 | CAV1 |
| MSLN | GIPC3 | EIF4EBP2 | IL17RA | ANXA11 | NOS3 | LYAR | SLC11A1 | KLHL36 | OASL |
| CDKN1A | HSPB6 | SERPINB1 | ABL1 | HMOX1 | DDR2 | B2M | STAT3 | GSDMD | PEA15 |
| IFITM3 | CNN3 | ELF1 | ARHGAP30 | DNAJB1 | SH3GLB1 | ADAMTS9 | TEAD4 | SLC8B1 | SNAP23 |
| CEBPA | PODN | EMP1 | WWTR1 | ICAM2 | HERC5 | PTPN1 | TGM2 | APOL4 | CFLAR |
| CEBPB | COL4A2 | EMP3 | HEYL | IFRD1 | SLC15A3 | CXCL16 | THBS1 | TRIM56 | SPOCD1 |
| IFITM2 | ADM | ALAS2 | CHIC2 | LILRA5 | PLA1A | JAM2 | TSPO | TKTL1 | CD14 |
| TRAF3IP2 | NACC2 | F2R | GEM | IL4R | SCARA3 | RRAGD | TIMP3 | FERMT3 | CD163 |
| VAMP5 | ZBTB46 | FCER1G | GJA4 | ITGA5 | MAP3K20 | RBMS2 | TLR5 | CRISPLD2 | CHST3 |
| FERMT2 | CSRP2 | CHSY1 | CYTH4 | RHOB | PIM1 | BCL6 | CLDN5 | STK40 | CD37 |
| BTN3A2 | CTSS | CD93 | GNAI2 | LCP1 | PLAUR | RP2 | C1QB | SRPX | CD44 |
| FAM107A | GLIPR2 | PCNX1 | SLCO4A1 | MGP | PODXL | RXRA | C1QC | TAGLN2 | CD81 |
| CMTM7 | CCDC50 | PALLD | SERTAD3 | MSN | PPARG | S100A8 | C5AR1 | TUBB6 | STARD8 |
| C1QTNF1 | RIBC1 | ZBTB43 | ANXA1 | MSX1 | TOR4A | S100A9 | VIM | HAVCR2 | FCHSD2 |
|  |  |  |  |  |  |  |  |  |  |
| Genes jointly downregulated in ASD and upregulated in lung cancer | | | | | | | | | |
|  |  |  |  |  |  |  |  |  |  |
| ACOT8 | MAL2 | SCAMP5 | PRPF19 | TRIM37 | ANAPC5 | ABCF3 | PTPN3 | ZNF195 | SEH1L |
| PARP2 | ZBED9 | ELAVL4 | GLRB | NDUFA7 | LCMT1 | STRBP | RAD1 | CACNA1F | ITCH |
| RWDD2B | AP1S1 | TMEM268 | GPI | NDUFA9 | RTCB | PREP | NECTIN1 | AUNIP | ARMC10 |
| TSFM | C12orf45 | OARD1 | RELL2 | NDUFB2 | GSKIP | SVOP | RABIF | DCTPP1 | PSMG3 |
| TRAP1 | CLTC | FKBP3 | NSMCE2 | NDUFV1 | RAB6B | NHP2 | RAD51C | RNF26 | PDCD2L |
| PSMD14 | PUSL1 | MRPS27 | MRPL22 | DRG1 | MRPS23 | AGK | DHX35 | NDUFAF5 | SELENOI |
| POP7 | KLHDC9 | RUSC1 | GSS | NEFH | PTRH2 | OGDHL | SMAP1 | ZNF576 | THOC5 |
| NPM2 | FBLN7 | SMPX | OLA1 | OAT | PFN2 | SLC25A40 | NEUROD6 | FSD1 | PEX11B |
| CD2BP2 | COL19A1 | MTOR | NXPH1 | ODF2 | PGK1 | MAPK9 | CEP85 | TTC13 | INPP4B |
| TIMM17A | DNAJC19 | TCERG1L | APEX1 | OPA1 | ATP6V1C1 | PSMB4 | FNDC4 | SNRNP25 | FUBP1 |
| TBL3 | RTP1 | GABRG2 | PPM1J | ORC4 | POLR2B | PSMB5 | MRPS11 | SRD5A3 | WASF1 |
| SPINT2 | COX6B1 | HECTD1 | IARS | OXCT1 | NLE1 | MRPS22 | MRPS9 | CCDC51 | INA |
| CGREF1 | SDHAF4 | DPCD | ICA1 | SLC35B3 | PRMT7 | PSMB7 | VPS33A | PDZD7 | SYNGR3 |
| GNB5 | NDUFAF6 | ATRNL1 | ZFP69 | COQ6 | DCAF16 | NIT2 | SST | DSN1 | KCNAB3 |
| NOXA1 | MUM1L1 | DCAF4 | KIAA2022 | GLRX2 | TEX10 | ANKMY2 | TBCC | C10orf88 | DCLK1 |
| CPLX2 | FAM81A | CABYR | STAC2 | MRPL4 | RPP25 | PSMD2 | TBCE | THOC7 | TMEM169 |
| NMU | C1orf74 | FBXO22 | HCN1 | MRPS7 | DUS2 | SLC17A6 | BYSL | GRPEL1 | UBE4A |
| RNPS1 | PDIK1L | SEZ6L2 | ITPA | TFB1M | RMND1 | C12orf4 | TM7SF2 | USP5 | SFXN1 |
| MLLT11 | CYC1 | TRUB2 | CCDC184 | ANKRD39 | PIH1D1 | LYRM4 | TPI1 | BHLHB9 | MED17 |
| SLC27A2 | RHOXF1 | INPP5J | SLC6A17 | CEND1 | OXR1 | ADCK1 | TRAF3 | KCNH6 | ENTPD3 |
| TDRKH | DDX1 | GLS2 | ARL1 | CD320 | SLC6A15 | PSMD10 | TSSC1 | ITFG1 | NUP93 |
| KAT7 | DHX36 | MMADHC | PLPP6 | RSRC1 | BRF2 | CAMK1G | UQCRFS1 | LOC81691 | TOMM70 |
| PDCD10 | DLX1 | AMPH | MAP3K9 | MRPS17 | C5orf22 | LRFN2 | ZNF165 | SLIRP | ACOT8 |
|  |  |  |  |  |  |  |  |  |  |
| Genes jointly upregulated in ASD and downregulated in prostate cancer | | | | | | | | | |
|  |  |  |  |  |  |  |  |  |  |
| LHFPL2 | FERMT2 | ZBTB46 | EMP3 | ANXA11 | NRTN | TMEM51 | SLC2A5 | ZFYVE21 | PEA15 |
| MPZL2 | FAM107A | CSRP2 | EYA2 | HSPB1 | DDR2 | RAVER2 | ZFP36L1 | C1orf54 | SERPINH1 |
| MSLN | CMTM7 | GLIPR2 | PALLD | ICAM2 | SH3GLB1 | C4orf19 | STAT3 | KLHL36 | NAV2 |
| IFITM3 | C1QTNF1 | CYBA | RAB38 | IFI16 | HERC5 | RBM38 | TIMP1 | GSDMD | EPSTI1 |
| YAP1 | PLIN2 | LIX1 | IL17RA | IFI35 | SCARA3 | RNF114 | TIMP3 | COL18A1 | CHST3 |
| CEBPB | GIPC3 | DPYSL3 | WWTR1 | IL4R | MAP3K20 | B2M | TLR5 | CRISPLD2 | BAG3 |
| IFITM2 | HSPB6 | EFNB1 | PITPNC1 | ITGA5 | PLAU | ADAMTS9 | CLDN5 | SRPX | CD44 |
| TRAF3IP2 | PODN | ANO6 | SLC39A1 | RHOB | PLTP | PBXIP1 | TPD52L2 | TAGLN2 | CD81 |
| VAMP5 | COL4A2 | SERPINB1 | GLI2 | RHOC | DDIT4 | JAM2 | UCP2 | ANTXR1 | STARD8 |
| ALDH1L1 | IL17RE | ELF1 | GNAI2 | LGALS3 | PPARG | RBMS2 | VEGFA | TUBB6 | FCHSD2 |
| HCP5 | EGFLAM | APOBEC3F | SFN | LPP | XAF1 | RXRA | VIM | AJUBA | MVP |
| MTMR11 | NACC2 | EMD | ANXA1 | MSN | FAM46C | S100P | CA12 | CAV1 | LHFPL2 |
|  |  |  |  |  |  |  |  |  |  |
| Genes jointly downregulated in ASD and upregulated in prostate cancer | | | | | | | | | |
|  |  |  |  |  |  |  |  |  |  |
| TSFM | C12orf45 | DHPS | SEZ6L2 | SAMD5 | ANAPC5 | PRKACB | SMAP1 | CCDC51 | SELENOI |
| TRAP1 | CLTA | DLX1 | TRUB2 | PLPP6 | GSKIP | AGK | CEP85 | ZFAND1 | INPP4B |
| PSMD14 | CLTC | SCAMP5 | MMADHC | MAP3K9 | RAB6B | OGDHL | MRPS9 | C10orf88 | BAIAP3 |
| POP7 | KLHDC9 | TMEM268 | GPI | TRIM37 | PTRH2 | DCAF6 | SLCO1A2 | THOC7 | CYTH2 |
| TIMM17A | TMEM18 | CNKSR2 | RIMKLA | ODF2 | ATP6V1C1 | SLC25A40 | BYSL | GRPEL1 | TOMM70 |
| MYBBP1A | SDHAF4 | FKBP3 | NSMCE2 | ORC4 | EXOSC9 | MAPK9 | TM7SF2 | INTS14 | TSFM |
| CAMKK2 | NDUFAF6 | ENPP4 | APEX1 | OXCT1 | NLE1 | PSMB5 | TSSC1 | SLIRP | TRAP1 |
| CGREF1 | MUM1L1 | RUSC1 | IARS | COQ6 | HEATR5B | PSMB7 | VCP | SEH1L | PSMD14 |
| SLC27A2 | FAM81A | MTOR | ICA1 | MRPS7 | C5orf22 | SMYD2 | ZNF165 | ARMC10 | POP7 |
| TDRKH | PDIK1L | DPCD | ACADSB | DYNC1LI1 | STRBP | NIT2 | ZNF195 | PSMG3 | TIMM17A |
| MAL2 | ZNF385B | DCAF4 | ITPA | CD320 | PREP | LYRM4 | DCTPP1 | PDCD2L | MYBBP1A |
| ZBED9 | CYC1 | SERGEF | C6orf120 | MRPS17 | PRKAA1 | RABIF | SRD5A3 | MPND | CAMKK2 |

**Table S4: Genes jointly upregulated and downregulated in ASD and SDDCs and genes jointly upregulated in ASD and downregulated in ODDCs and genes jointly downregulated in ASD and upregulated in ODDCs.**

**Table S5:**

**
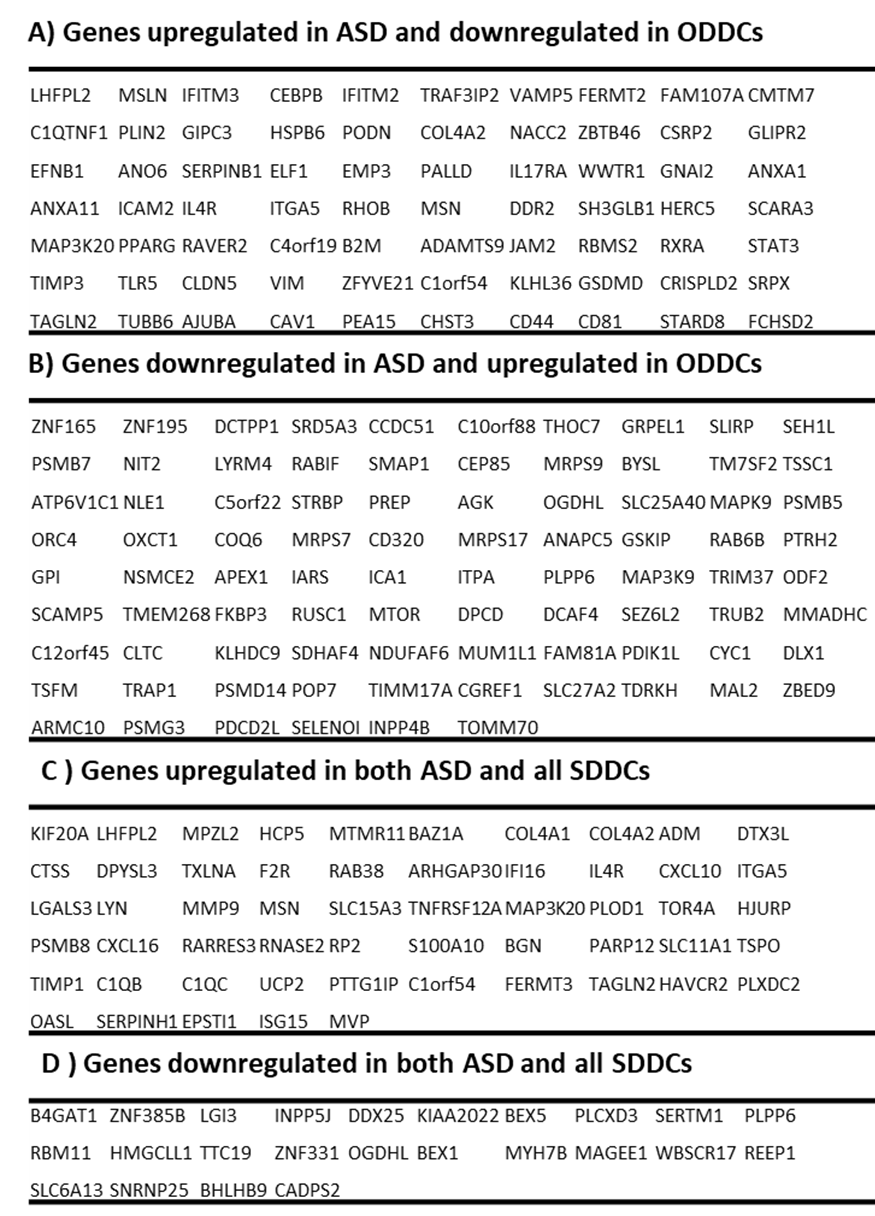
**

**Table S5: Genes commonly deregulated in ASD and in SDDCs and ODDCs, including a) genes upregulated in ASD and downregulated in both ODDCs, b) genes downregulated in ASD and upregulated in both ODDCs, c) genes upregulated in both ASD and all SDDCs, and d) genes downregulated in both ADS and all SDDCs.**

**SUPPLEMENTARY FIGURES**

**Figure S1:**

In order to check validity of RNA-Seq RLOG transformed data inclusion in the Choi meta-analysis we compared differential expression results of the ASD RNA-Seq dataset using two different methods. First we performed differential expression analysis using two state of the art methods. DESeq2 and Limma Voom implemented in the DESeq2 and limma packages respectively. Then we used RLOG transformed data to perform traditional limma based differential expression analysis. Log fold changes derived from differential expression analysis were then compared by correlation. The log fold changes obtained by both methods were highly correlated showing Spearman correlation values of 0.87 and 0.90 respectively. The correlations value of the differential expression results derived from DESeq2 and Limma Voom was 0.78.


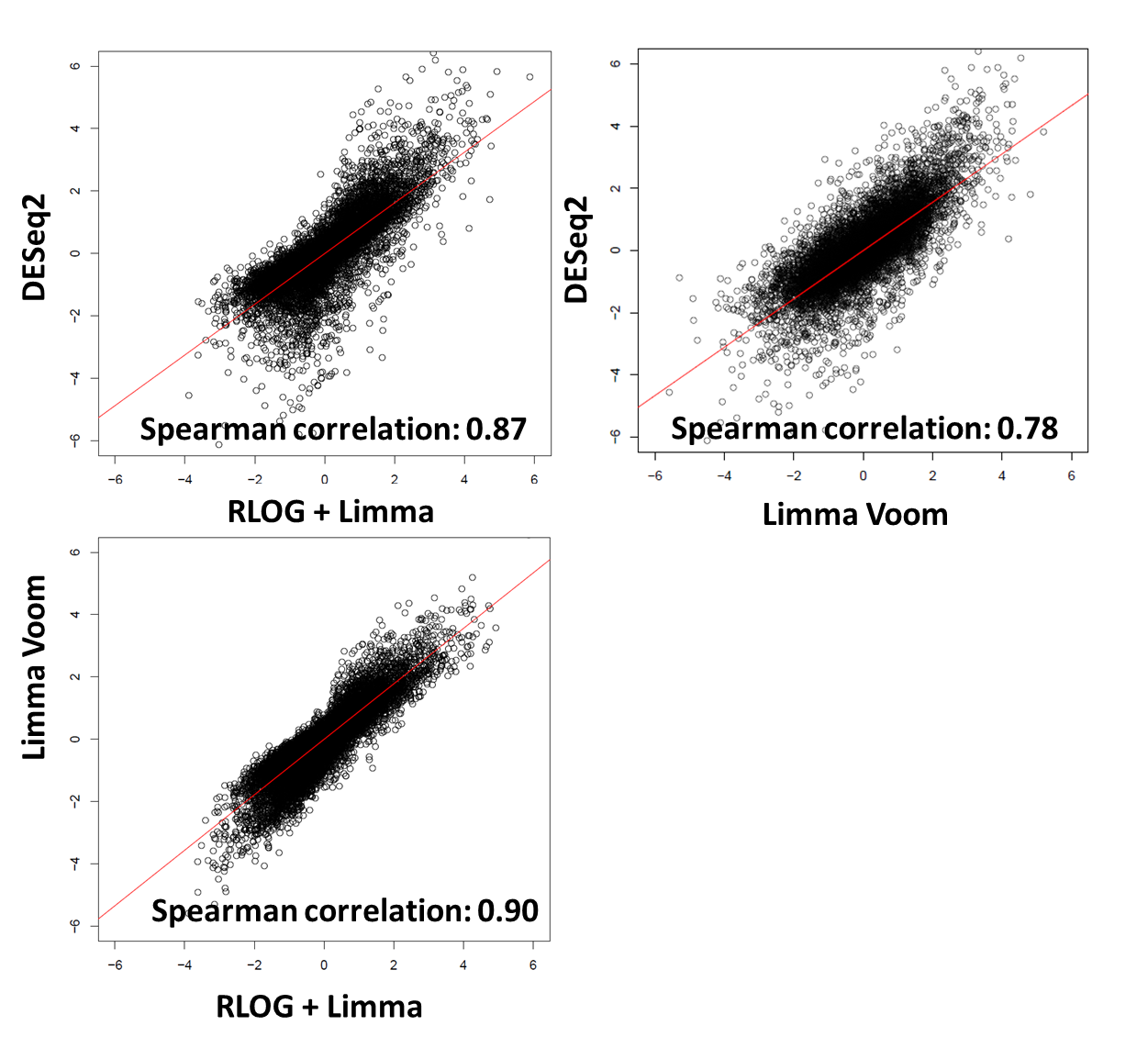

**figure S1: Comparison between RNA-Seq differential expression state of the art methods and Rlog transformation followed by limma differential expression analysis.**

DESeq2 yielded 390 differentially expressed genes under an FDR corrected pvalue of 0.05. No differentially expressed genes were found under the same threshold in the Limma Voom or RLOG+Limma analysis. In summary, RLOG transformatio produces differential expression results that are well correlated to two of the state of the art RNAseq differential expression methdos. Moreover, the yield in terms of differentially expressed genes is more conservative thant the other two methods. We reason that this observations meke fair the inclussion of RNAseq RLOG transformed data in the Choi differential gene expression meta-anaysls.

**Figure S2:**

**ASD SAMPLES**

**CONTROL SAMPLES**

**
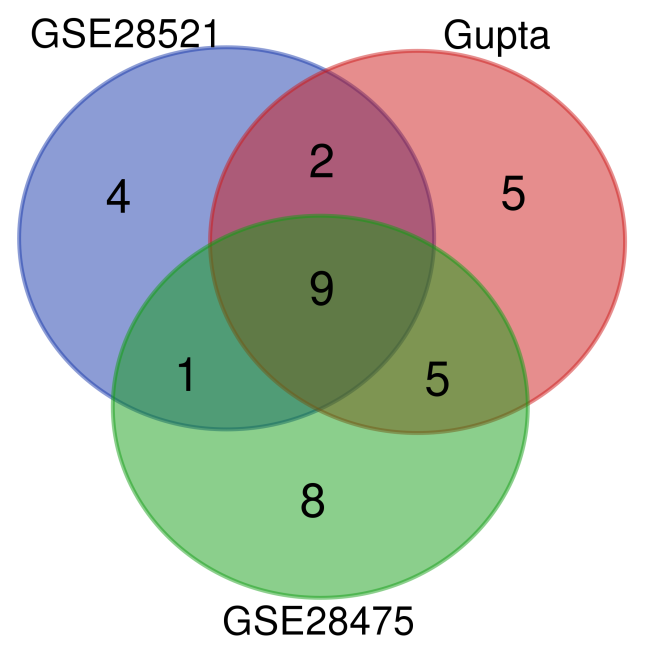
**
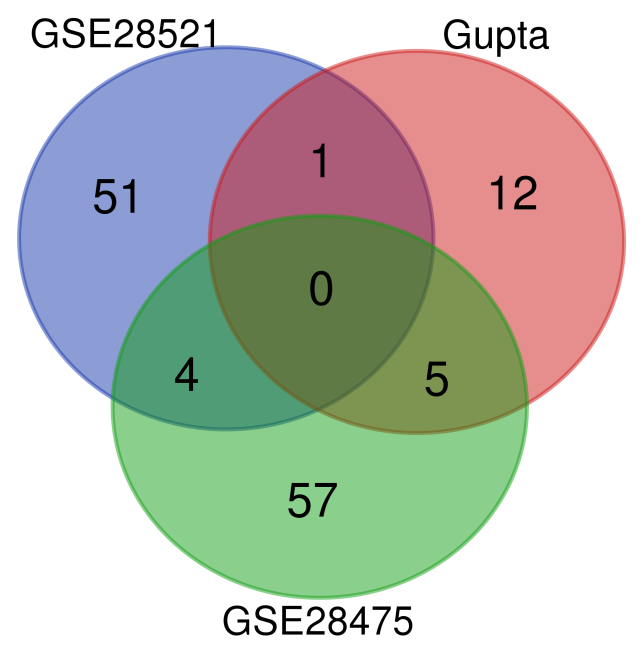


**Figure S2: Venn diagram showing the number of frontal cortex samples derived from the same ASD (A) and control (B) individuals present in the three included studies prior to redundant sample removal.**

**Figure S3:**

**
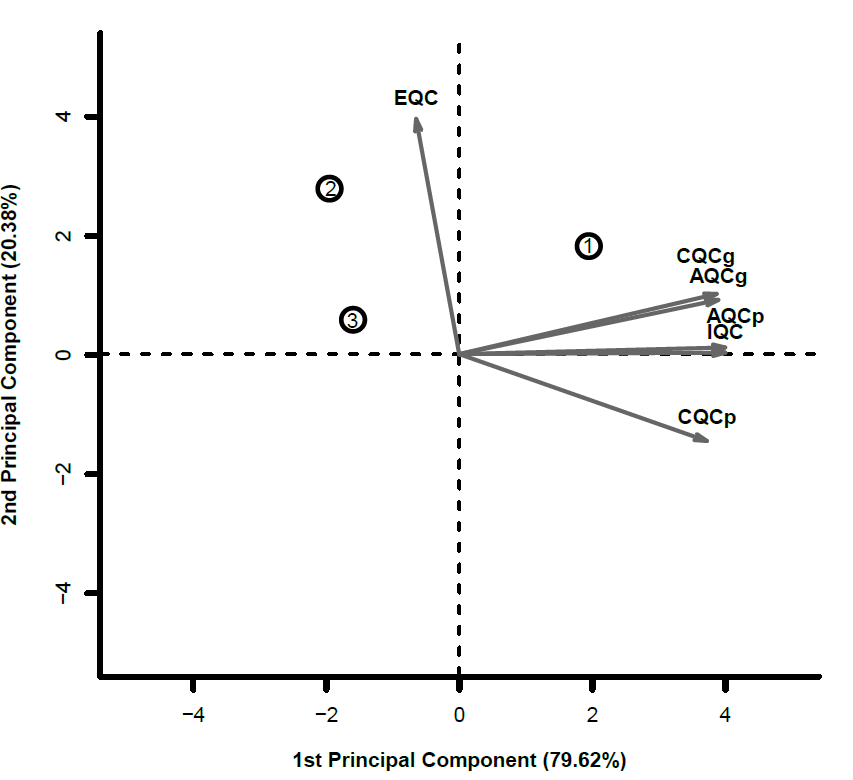
**

**A)**

**B)**

| **Study** | **IQC** | **EQC** | **CQCg** | **CQCp** | **AQCg** | **AQCp** | **SMR** |
| --- | --- | --- | --- | --- | --- | --- | --- |
| **GSE28521** | 20 | 3.8 | 27.42 | 8.5 | 23.9 | 0.67* | 1.17 |
| **Gupta** | 2.67 | 5 | 9.37 | 2.56 | 6.9 | 0.08* | 2.33 |
| **GSE28475** | 4 | 2.91 | 5.12 | 5.14 | 3.55 | 0.11* | 2.5 |

**C)**

| Study | Mean Inter-array correlation |
| --- | --- |
| GSE28521 | 0.96 |
| Gupta | 0.95 |
| GSE28475 | 0.91 |

**figure S3: A) PCA plot showing a 2D projection of the 6 quality scores computed for the 3 ASD datasets by MetaQC. B) Values for the 6 quality scores computed for the 3 ASD datasets. The last column shows the quality summary value (SMR) which was used together with the mean inter-sample correlation to determine the quality order of the studies for duplicated patient removal purposes.**

**Figure S4:**


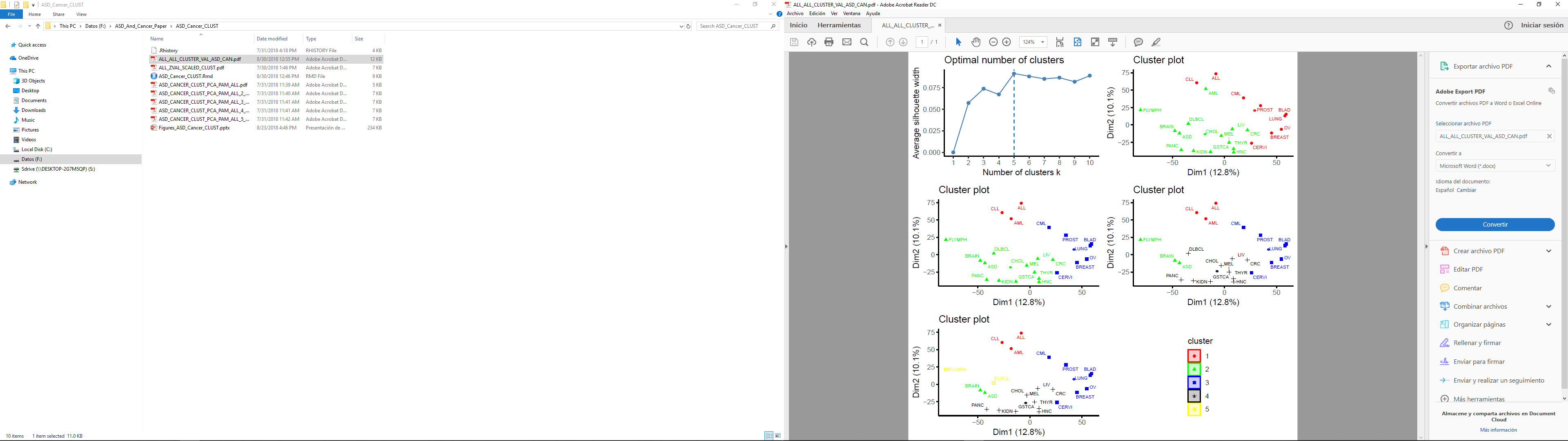


**E)**

**D)**

**C)**

**B)**

**A)**

**Figure S4: Partition Around Medoids (PAM) clustering of the differential gene expression profiles of ASD and the 22 included cancer types. A) Silhouette analysis showing the optimal number of clusters. B) Two partitions. C) Three partitions. D) Four partitions. E) Five partitions.**

**Figure S5:**

**
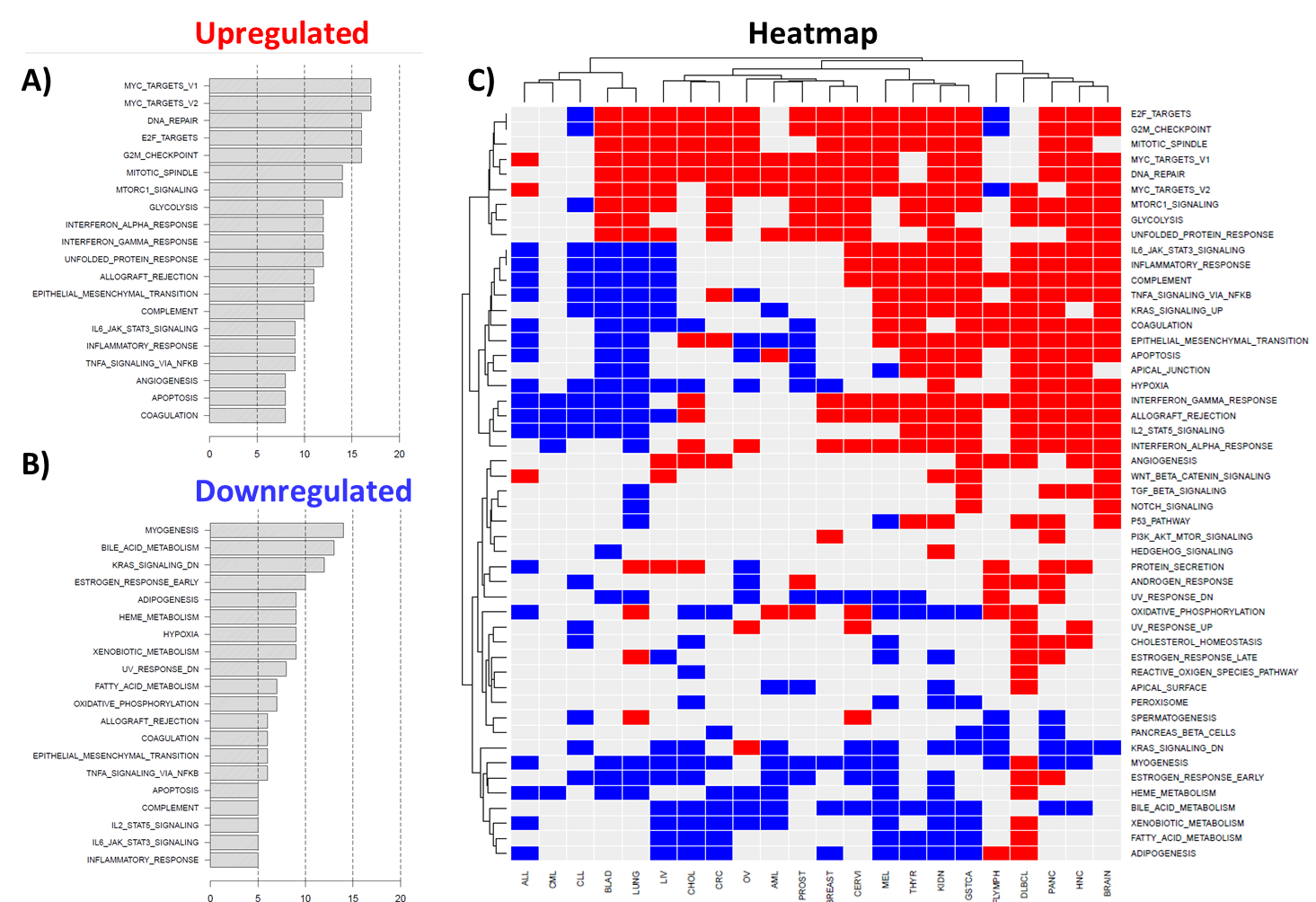
**

**Figure S5: A) Top 20 upregulated Hallmark molecular signatures. Bar lengths represent the number of cancers in which each pathway was found upregulated. B) Top 20 downregulated Hallmark molecular signatures. Bar lengths represent the number of cancers in which each pathway was found downregulated. C) Heatmap showing upregulated (red) and downregulated (blue) Hallmark molecular signatures in the 22 studied cancer types.**

**Figure S6:**

**
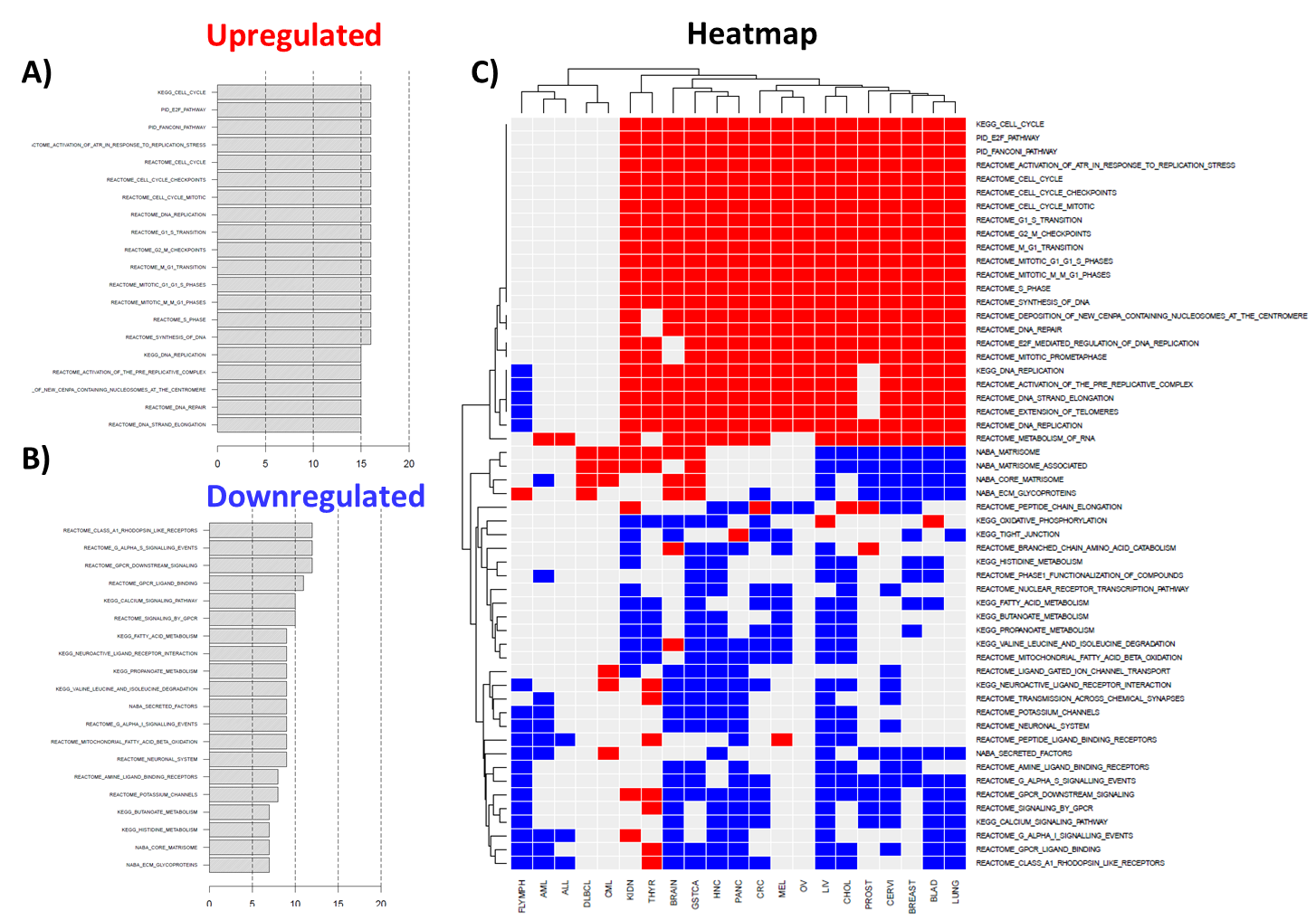
**

**figure S6: A) Top 20 upregulated canonical pathways molecular signatures. Bar lengths represent the number of cancers in which each pathway was found upregulated. B) Top 20 downregulated canonical pathways molecular signatures. Bar lengths represent the number of cancers in which each pathway was found downregulated. C) Heatmap showing upregulated (red) and downregulated (blue) canonical pathways molecular signatures in the 22 studied cancer types.**

**Figure S7:**

**
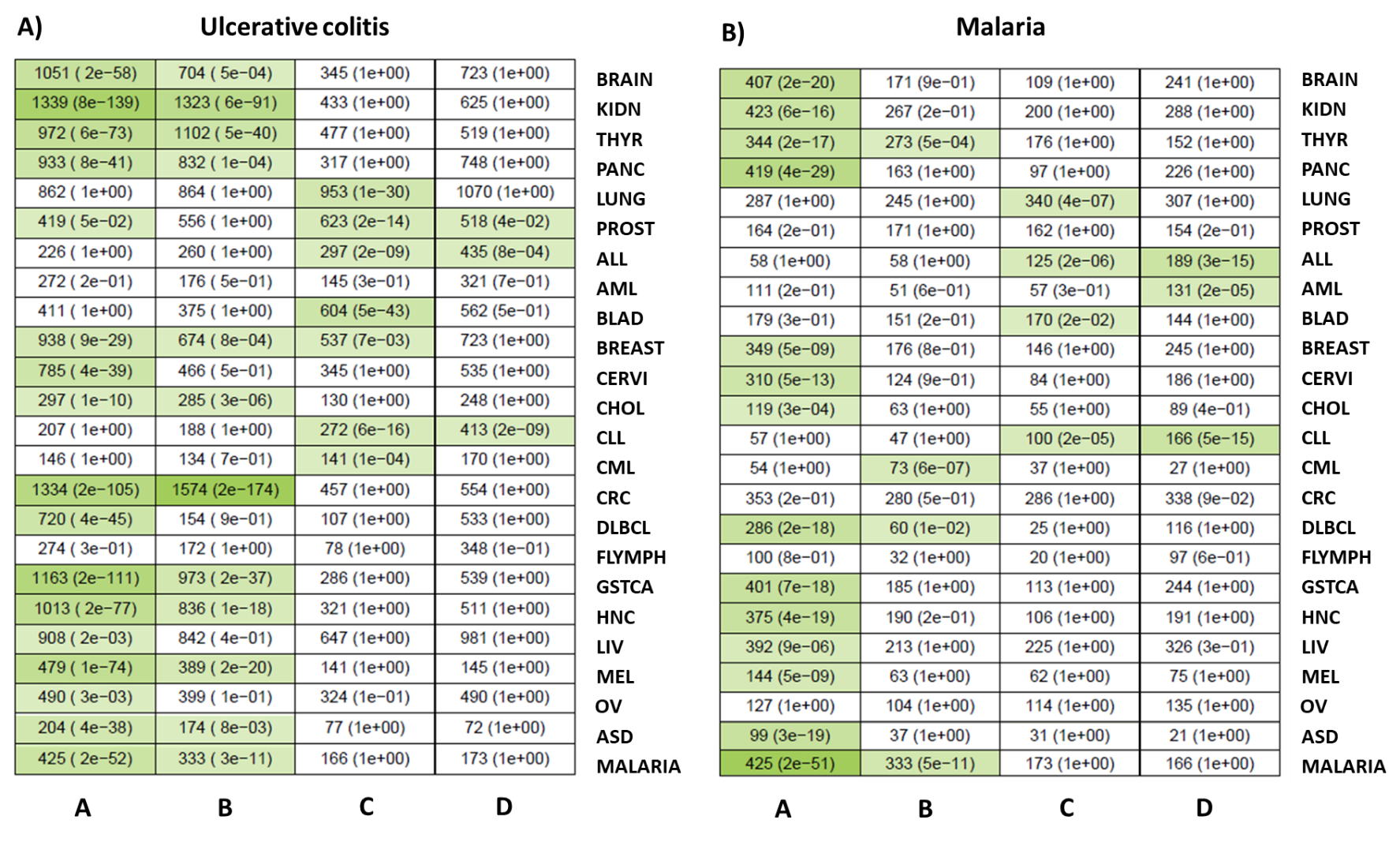
**

**Figure S7: A) Table showing the significance of the intersections of upregulated and downregulated genes between Ulcerative colitis and ASD, malaria, and the 22 cancer types included in our study. B) Table showing the significance of the intersections of upregulated and downregulated genes between malaria and ASD, ulcerative colitis, and the 22 cancer types included in our study. The included cancers were acute lymphoblastic leukemia (ALL), acute myeloid leukemia (AML), bladder cancer (BLAD), brain cancer (BRAIN), breast cancer (BREAST), cervical cancer (CERVI), cholangiocarcinoma (CHOL), chronic lymphocytic leukemia (CLL), chronic myeloid leukemia (CML), colorectal cancer (CRC), diffuse large b cell lymphoma (DLBCL), follicular lymphoma (FLYMPH), gastric cancer (GSTCA), head and neck carcinoma (HNC), kidney cancer (KIDN), liver cancer (LIV), lung cancer (LUNG), melanoma (MEL), ovarian cancer (OV), pancreatic cancer (PANC), prostate cancer (PROST), and thyroid cancer (THYR). Columns A, B, C, and D include the number of genes upregulated in both, downregulated in both, upregulated in ASD and downregulated in cancer, and downregulated in ASD and upregulated in cancer, respectively. Green cell colors indicate significant intersections (FDR corrected p-values from Fisher’s exact test lower than 0.05) with darker green tones indicating lower FDR corrected p-values.**

**Figure S8:**

**
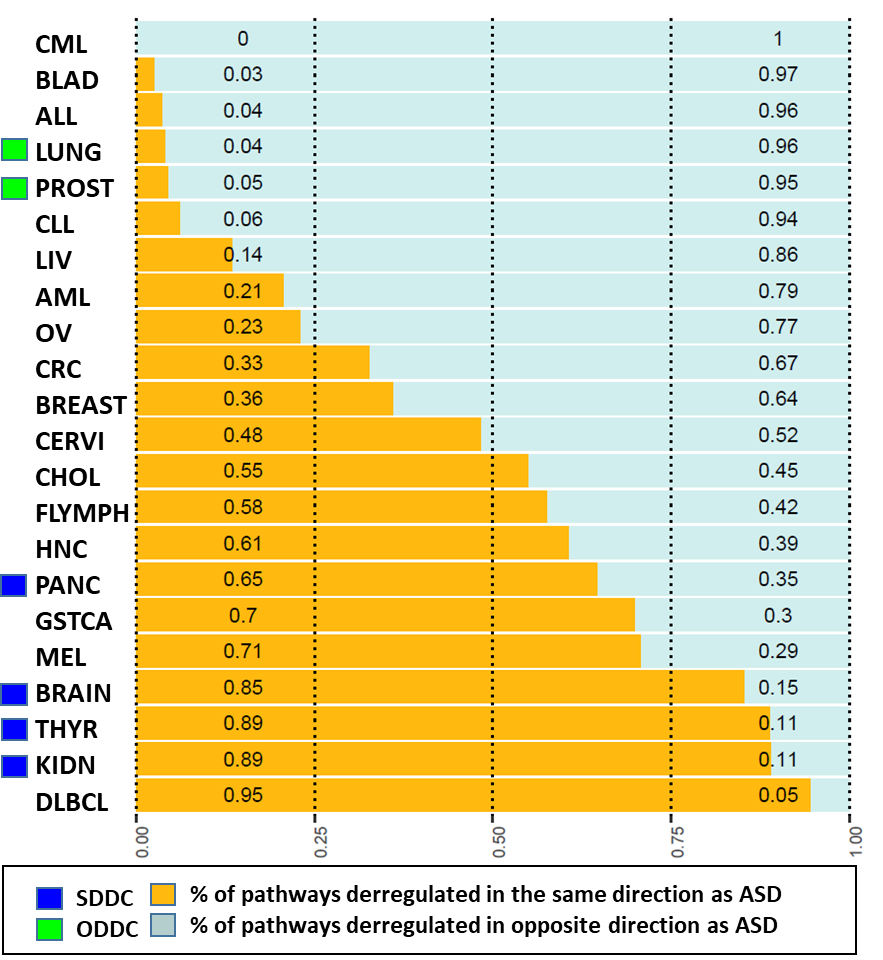
**

**figure S8: Percentage of Reactome, KEGG and Hallmarks canonical pathways deregulated in the same (golden) and opposite (light blue) directions between ASD and cancer. B) KEGG (K), Reactome (R) and Hallmarks (H) pathway deregulation in ASD, SDDCs and ODDCs.**

APENDIX I: CODE

AI.1) GSE28521 preprocessing

AI.2) GSE28475 preprocessing

AI.3) Gupta’s preprocessing

AI.4) ASD differential gene expression meta-analysis

AI.5) Functions

AI 1) GSE28521 preprocessing

Clearing all variables.

rm(list=ls())
gc()

options(stringsAsFactors = F)

SouRcing

source("H:/JProg/R_prog/Basic_Functions.R")
source("H:/JProg/R_prog/Working_With_Arrays.R")
source("H:/JProg/R_prog/Working_With_GEO.R")

Loading libraries.

library("Hmisc")
library("GEOquery")
library("limma")
library("lumi")
library("illuminaHumanv3.db")
library("illuminaHumanv4.db")
library("xtable")
library("cluster")

Setting working directory.

Create_Structure("/Dir/","GSE28521")
setwd("Dir/GSE28521/")
getwd()

Reading phenotype info from the soft file.

#Getting pData from soft.

GSE28521 <- getGEO(filename="./Data/GSE28521_family.soft.gz")
platforms <- unlist(lapply(GPLList(GSE28521),function(x) Meta(x)$geo_accession))
pData_GSE28521 <- get_pData_table_general(GSE28521,platforms[[1]])
pData_GSE28521$pCh_patient <- as.character(unlist(lapply(strsplit(pData_GSE28521$title,"_"),function(x) x[[2]])))
rownames(pData_GSE28521) <- pData_GSE28521$description

#Adding supplementary table info.

pheno_Extra <- read.csv(file = "./PaperNotesTables/ExtraPheno.txt",sep=" ")
pData_GSE28521 <- merge(pData_GSE28521,pheno_Extra,by.x="pCh_patient",by.y = "Case_Id",all.x = TRUE)
colnames(pData_GSE28521)[c(35,36,37,38,39)] <- paste("pCh_",colnames(pData_GSE28521)[c(35,36,37,38,39)],sep="")
colnames(pData_GSE28521)[c(35,36,37,38,39)]
rownames(pData_GSE28521) <- pData_GSE28521$description
pData_GSE28521$pCh_patient <- gsub("._","",gsub("_.{1}$","",pData_GSE28521$title))
pData_GSE28521$patient <- gsub("_.*","",gsub("^.{1}_","",pData_GSE28521$title))
pData_GSE28521 <- pData_GSE28521[,c("pCh_patient","pCh_disease_status","pCh_tissue_.brain_region.","pCh_AGE","pCh_SEX","pCh_PMI","pCh_Tissue_region")]
colnames(pData_GSE28521) <- c("pCh_Patient_ID","pCh_Diagnosis","pCh_Brain_Region","pCh_Age","pCh_Gender","pCh_PMI","pCh_Brain_Region_2")
pData_GSE28521$pCh_Study <- rep("GSE28521",nrow(pData_GSE28521))
pData_GSE28521
pCh_Diagnosis <- pData_GSE28521$pCh_Diagnosis
pCh_Diagnosis[grepl("autism",pCh_Diagnosis)] <- "Autism"
pCh_Diagnosis[grepl("controls",pCh_Diagnosis)] <- "Control"
pData_GSE28521$pCh_Diagnosis <- pCh_Diagnosis
colnames(pData_GSE28521)[7] <- "pCh_Broadman_Area"

Read non normalized expression data file.

eData_GSE28521 <- read.ilmn("./Data/GSE28521_non-normalized_data.txt")
eData_GSE28521 <- eData_GSE28521$E

Creating an expressionSet

intersected <- intersect(colnames(eData_GSE28521),rownames(pData_GSE28521))
eData_GSE28521 <- eData_GSE28521[,intersected]
pData_GSE28521 <- pData_GSE28521[intersected,]
pheno <- as(pData_GSE28521,"AnnotatedDataFrame")
GSE28521_Eset_Raw <- new('ExpressionSet',exprs=eData_GSE28521,phenoData=pheno)

GSE28521_FCTX_Eset_Raw <- GSE28521_Eset_Raw[,pData(GSE28521_Eset_Raw)$pCh_Brain_Region == "Frontal_cortex"]
GSE28521_FCTX_Eset_Raw

pCh_Individual <- pData(GSE28521_FCTX_Eset_Raw)$pCh_Patient_ID
pData(GSE28521_FCTX_Eset_Raw)$pCh_Individual <- pCh_Individual

GSE36192_FCTX_Eset_Raw <- get(load(file = "H:/AutAndCanDef/Data/ASD_2/Auxiliar_Data/GSE36192/Data/GSE36192_FCTX_Eset_Raw_Not_Sampled.Rda"))
intersected <- intersect(rownames(GSE36192_FCTX_Eset_Raw),rownames(GSE28521_FCTX_Eset_Raw))

pheno_Samp <- pData(GSE36192_FCTX_Eset_Raw)

pheno_Samp$pCh_Age <- as.numeric(pheno_Samp$pCh_Age)

list_conv <- list()
list_conv[[2]] <- 24.56
list_conv_sub <- list()
list_conv_sub[["F"]] <- as.numeric(table(pData(GSE28521_FCTX_Eset_Raw)$pCh_Gender[pData(GSE28521_FCTX_Eset_Raw)$pCh_Diagnosis == "Autism"])/sum(table(pData(GSE28521_FCTX_Eset_Raw)$pCh_Gender[pData(GSE28521_FCTX_Eset_Raw)$pCh_Diagnosis == "Autism"])))[1]
list_conv_sub[["M"]] <- as.numeric(table(pData(GSE28521_FCTX_Eset_Raw)$pCh_Gender[pData(GSE28521_FCTX_Eset_Raw)$pCh_Diagnosis == "Autism"])/sum(table(pData(GSE28521_FCTX_Eset_Raw)$pCh_Gender[pData(GSE28521_FCTX_Eset_Raw)$pCh_Diagnosis == "Autism"])))[2]
list_conv[[1]] <- list_conv_sub

out_test <- optimum_n_selector_2(pheno_Samp,cols = c(2,3),list_conv,number_of_rep=200)

vec_vals <- c(mean(pData(GSE28521_FCTX_Eset_Raw)$pCh_Age[pData(GSE28521_FCTX_Eset_Raw)$pCh_Diagnosis == "Autism"]),as.numeric(table(pData(GSE28521_FCTX_Eset_Raw)$pCh_Gender[pData(GSE28521_FCTX_Eset_Raw)$pCh_Diagnosis == "Autism"])/sum(table(pData(GSE28521_FCTX_Eset_Raw)$pCh_Gender[pData(GSE28521_FCTX_Eset_Raw)$pCh_Diagnosis == "Autism"]))))

out_test_filt <- out_test[[1]]

out_test_filt <- out_test_filt[((out_test_filt$vec_mean < list_conv[[2]] + 1) & (out_test_filt$vec_mean > list_conv[[2]] - 1)) & ((out_test_filt[,4] < (list_conv[[1]][[1]] + 0.01)) &(out_test_filt[,4] > (list_conv[[1]][[1]] - 0.01))),]

out_test_filt_40 <- out_test_filt[out_test_filt$vec_n == 40,]

selelected_sample <- sample(rownames(out_test_filt_40),1)

sample_temp_vec <- out_test[[2]][[as.numeric(gsub("SAMPLE_","",selelected_sample))
]]

GSE36192_FCTX_Eset_Raw_Sampled <- GSE36192_FCTX_Eset_Raw[,sample_temp_vec]

GSE28521_FCTX_Eset_Raw <- GSE28521_FCTX_Eset_Raw[intersected,]
GSE36192_FCTX_Eset_Raw_Sampled <- GSE36192_FCTX_Eset_Raw_Sampled[intersected,]

GSE28521_AND_GSE36192_FCTX_Exp <- cbind(exprs(GSE28521_FCTX_Eset_Raw),exprs(GSE36192_FCTX_Eset_Raw_Sampled))

GSE28521_AND_GSE36192_FCTX_Pheno <- rbind.fill(pData(GSE28521_FCTX_Eset_Raw),pData(GSE36192_FCTX_Eset_Raw_Sampled))

rownames(GSE28521_AND_GSE36192_FCTX_Pheno) <- c(rownames(pData(GSE28521_FCTX_Eset_Raw)),rownames(pData(GSE36192_FCTX_Eset_Raw_Sampled)))

GSE28521_AND_GSE36192_FCTX_Pheno$pCh_Patient_ID[33:length(GSE28521_AND_GSE36192_FCTX_Pheno$pCh_Patient_ID)] <- GSE28521_AND_GSE36192_FCTX_Pheno$pCh_Individual[33:length(GSE28521_AND_GSE36192_FCTX_Pheno$pCh_Individual)]

pheno <- as(GSE28521_AND_GSE36192_FCTX_Pheno,"AnnotatedDataFrame")
GSE28521_AND_GSE36192_FCTX_Eset_Raw <- new('ExpressionSet',exprs=GSE28521_AND_GSE36192_FCTX_Exp,phenoData=pheno)
pCh_Diagnosis <- pData(GSE28521_AND_GSE36192_FCTX_Eset_Raw)$pCh_Diagnosis

pCh_Diagnosis[is.na(pCh_Diagnosis)] <- "Control"
pData(GSE28521_AND_GSE36192_FCTX_Eset_Raw)$pCh_Diagnosis <- pCh_Diagnosis
pData(GSE28521_AND_GSE36192_FCTX_Eset_Raw)$pCh_Diagnosis
pData(GSE28521_AND_GSE36192_FCTX_Eset_Raw)$pCh_Individual

Generating quality plots before preprocessing

pdf(file = "./Results/density_FCTX_Pre_Norm.pdf")
lumi::density(GSE28521_AND_GSE36192_FCTX_Eset_Raw,legend = FALSE)
dev.off()
pdf(file = "./Results/box_FCTX_Pre_Norm.pdf")
lumi::boxplot(GSE28521_AND_GSE36192_FCTX_Eset_Raw)
dev.off()
pn <- pData_to_pData_n(pData(GSE28521_AND_GSE36192_FCTX_Eset_Raw))


pdf(file = "./Results/PCA_FCTX_Pre_Norm_Status.pdf")
plotPCA(GSE28521_AND_GSE36192_FCTX_Eset_Raw,groups = pn$pCh_Diagnosis,groupnames = unique(pData(GSE28521_AND_GSE36192_FCTX_Eset_Raw)$pCh_Diagnosis),legend = T)
dev.off()

group_batch <- pData(GSE28521_AND_GSE36192_FCTX_Eset_Raw)$pCh_Study
group_batch
group_batch_num <- as.numeric(factor(group_batch))

pdf(file = "./Results/PCA_FCTX_Pre_Norm_Status_Batch.pdf")
affycoretools::plotPCA(GSE28521_AND_GSE36192_FCTX_Eset_Raw,groups =group_batch_num,groupnames = levels(factor(unique(group_batch))),legend = T)
dev.off()

IAC=cor(exprs(GSE28521_AND_GSE36192_FCTX_Eset_Raw),use="p")
pdf(file = "./Results/IAC_Hist_FCTX_Pre_Norm.pdf")
hist(IAC,sub=paste("Mean=",format(mean(IAC[upper.tri(IAC)]),digits=3)),breaks=100)
dev.off()
cluster1=hclust(as.dist(1-IAC),method="average")
pdf(file = "./Results/IAC_Clust_FCTX_Pre_Norm.pdf")
plot(cluster1,cex=0.7,labels=dimnames(GSE28521_AND_GSE36192_FCTX_Eset_Raw)[[2]])
dev.off()
meanIAC=apply(IAC,2,mean)
sdCorr=sd(meanIAC)
numbersd=(meanIAC-mean(meanIAC))/sdCorr
pdf(file = "./Results/IAC_Dev_FCTX_From_Mean_IAC_Pre_Norm.pdf")
plot(numbersd)
abline(h=-2)
dev.off()

Outlier sample removal

group <- paste(pData(GSE28521_AND_GSE36192_FCTX_Eset_Raw)$pCh_Diagnosis,pData(GSE28521_AND_GSE36192_FCTX_Eset_Raw)$pCh_Study)
GSE28521_AND_GSE36192_FCTX_Eset_Raw <- SR_IAC_Eset(GSE28521_AND_GSE36192_FCTX_Eset_Raw,group,0.9)

GSE28521_AND_GSE36192_FCTX_Eset_Raw
GSE28521_AND_GSE36192_FCTX_Eset_Raw_bgc <- backgroundCorrect(exprs(GSE28521_AND_GSE36192_FCTX_Eset_Raw), method="normexp",normexp.method = "rma",offset = 50)

GSE28521_AND_GSE36192_FCTX_Eset_Raw_bgc_norm <- normalizeBetweenArrays(GSE28521_AND_GSE36192_FCTX_Eset_Raw_bgc, method="quantile")

GSE28521_AND_GSE36192_FCTX_Eset_Raw_bgc_norm_log2 <- log2(GSE28521_AND_GSE36192_FCTX_Eset_Raw_bgc_norm)

GSE28521_AND_GSE36192_FCTX_Eset_Proc <- GSE28521_AND_GSE36192_FCTX_Eset_Raw
exprs(GSE28521_AND_GSE36192_FCTX_Eset_Proc) <- GSE28521_AND_GSE36192_FCTX_Eset_Raw_bgc_norm_log2

Generating quality plots after preprocessing

pdf(file = "./Results/density_FCTX_Post_Norm.pdf")
lumi::density(GSE28521_AND_GSE36192_FCTX_Eset_Proc,legend = FALSE)
dev.off()

pdf(file = "./Results/box_FCTX_Post_Norm.pdf")
lumi::boxplot(GSE28521_AND_GSE36192_FCTX_Eset_Proc)
dev.off()
pn <- pData_to_pData_n(pData(GSE28521_AND_GSE36192_FCTX_Eset_Proc)
 )
pdf(file = "./Results/PCA_FCTX_Post_Norm_Status.pdf")
plotPCA(GSE28521_AND_GSE36192_FCTX_Eset_Proc,groups = pn$pCh_Diagnosis,groupnames = unique(pData(GSE28521_AND_GSE36192_FCTX_Eset_Proc)$pCh_Diagnosis),legend = T)
dev.off()
group_batch <- pData(GSE28521_AND_GSE36192_FCTX_Eset_Proc)$pCh_Study
group_batch_num <- as.numeric(factor(group_batch))

pdf(file = "./Results/PCA_FCTX_Post_Norm_Status_Batch.pdf")
affycoretools::plotPCA(GSE28521_AND_GSE36192_FCTX_Eset_Proc,groups =group_batch_num,groupnames = levels(factor(unique(group_batch))),legend = T)
dev.off()

IAC=cor(exprs(GSE28521_AND_GSE36192_FCTX_Eset_Proc),use="p")
pdf(file = "./Results/IAC_Hist_FCTX_Post_Norm.pdf")
hist(IAC,sub=paste("Mean=",format(mean(IAC[upper.tri(IAC)]),digits=3)),breaks=100)
dev.off()

cluster1=hclust(as.dist(1-IAC),method="average")
pdf(file = "./Results/IAC_Clust_FCTX_Post_Norm.pdf")
plot(cluster1,cex=0.7,labels=dimnames(GSE28521_AND_GSE36192_FCTX_Eset_Proc)[[2]])
dev.off()

meanIAC=apply(IAC,2,mean)
sdCorr=sd(meanIAC)
numbersd=(meanIAC-mean(meanIAC))/sdCorr
pdf(file = "./Results/IAC_Dev_FCTX_From_Mean_IAC_Post_Norm.pdf")
plot(numbersd)
abline(h=-2)
dev.off()

Batch effect correction

library(sva)
batch = pData(GSE28521_AND_GSE36192_FCTX_Eset_Proc)$pCh_Study
modcombat = model.matrix(~as.factor(pCh_Diagnosis), data=pData(GSE28521_AND_GSE36192_FCTX_Eset_Proc))
combat_edata = ComBat(dat=exprs(GSE28521_AND_GSE36192_FCTX_Eset_Proc), batch=batch, mod=modcombat, par.prior=TRUE, prior.plots=FALSE)
help(ComBat)
exprs(GSE28521_AND_GSE36192_FCTX_Eset_Proc) <- combat_edata
pData(GSE28521_AND_GSE36192_FCTX_Eset_Proc)

Saving data

save(file = "./Data/GSE28521_AND_GSE36192_FCTX_Eset_Proc.Rda",GSE28521_AND_GSE36192_FCTX_Eset_Proc)
GSE28521_AND_GSE36192_FCTX_Eset_Proc <- get(load(file="./Data/GSE28521_AND_GSE36192_FCTX_Eset_Proc.Rda"))

Generating quality plots after batch effect removal

pdf(file = "./Results/density_FCTX_Post_Batch_Norm.pdf")
lumi::density(GSE28521_AND_GSE36192_FCTX_Eset_Proc,legend = FALSE)
dev.off()
pdf(file = "./Results/box_FCTX_Post_Batch_Norm.pdf")
lumi::boxplot(GSE28521_AND_GSE36192_FCTX_Eset_Proc)
dev.off()
pn <- pData_to_pData_n(pData(GSE28521_AND_GSE36192_FCTX_Eset_Proc))

pdf(file = "./Results/PCA_FCTX_Post_Batch_Norm_Status.pdf")
plotPCA(GSE28521_AND_GSE36192_FCTX_Eset_Proc,groups = pn$pCh_Diagnosis,groupnames = unique(pData(GSE28521_AND_GSE36192_FCTX_Eset_Proc)$pCh_Diagnosis),legend = T)
dev.off()

group_batch <- pData(GSE28521_AND_GSE36192_FCTX_Eset_Proc)$pCh_Study
group_batch
group_batch_num <- as.numeric(factor(group_batch))
pdf(file = "./Results/PCA_FCTX_Post_Batch_Norm_Status_Batch.pdf")
affycoretools::plotPCA(GSE28521_AND_GSE36192_FCTX_Eset_Proc,groups =group_batch_num,groupnames = levels(factor(unique(group_batch))),legend = T)
dev.off()

IAC=cor(exprs(GSE28521_AND_GSE36192_FCTX_Eset_Proc),use="p")
pdf(file = "./Results/IAC_Hist_FCTX_Post_Batch_Norm.pdf")
hist(IAC,sub=paste("Mean=",format(mean(IAC[upper.tri(IAC)]),digits=3)),breaks=100)
dev.off()

cluster1=hclust(as.dist(1-IAC),method="average")
pdf(file = "./Results/IAC_Clust_FCTX_Post_Batch_Norm.pdf")
plot(cluster1,cex=0.7,labels=dimnames(GSE28521_AND_GSE36192_FCTX_Eset_Proc)[[2]])
dev.off()

meanIAC=apply(IAC,2,mean)
sdCorr=sd(meanIAC)
numbersd=(meanIAC-mean(meanIAC))/sdCorr
pdf(file = "./Results/IAC_Dev_FCTX_From_Mean_IAC_Post_Batch_Norm.pdf")
plot(numbersd)
abline(h=-2)
dev.off()

Preparing for meta-analysis

x <- illuminaHumanv3ENTREZID
#x <- illuminaHumanWGDASLv3ENTREZID
GSE28521_AND_GSE36192_FCTX_coll_MaxMean <- collapse_rows(x,GSE28521_AND_GSE36192_FCTX_Eset_Proc,Method = "MaxMean")
dim(GSE28521_AND_GSE36192_FCTX_coll_MaxMean)

GSE28521_AND_GSE36192_FCTX_coll_Average <- collapse_rows(x,GSE28521_AND_GSE36192_FCTX_Eset_Proc,Method = "Average")

GSE28521_AND_GSE36192_FCTX_coll_MaxRowVar <- collapse_rows(x,GSE28521_AND_GSE36192_FCTX_Eset_Proc,"maxRowVariance")

factor <- pData(GSE28521_AND_GSE36192_FCTX_Eset_Proc)$pCh_Diagnosis
factor[factor == "Autism"] <- 1
factor[factor == "Control"] <- 0
table(factor)

GSE28521_AND_GSE36192_FCTX_MM <- list(GSE28521_AND_GSE36192_FCTX_coll_MaxMean,factor)
GSE28521_AND_GSE36192_FCTX_AV <- list(GSE28521_AND_GSE36192_FCTX_coll_Average,factor)
GSE28521_AND_GSE36192_FCTX_MRV <- list(GSE28521_AND_GSE36192_FCTX_coll_MaxRowVar,factor)

save(file="./Data/GSE28521_AND_GSE36192_FCTX_MM.Rda",GSE28521_AND_GSE36192_FCTX_MM)
save(file="./Data/GSE28521_AND_GSE36192_FCTX_AV.Rda",GSE28521_AND_GSE36192_FCTX_AV)
save(file="./Data/GSE28521_AND_GSE36192_FCTX_MRV.Rda",GSE28521_AND_GSE36192_FCTX_MRV)

AI.2) GSE28475 preprocessing

Clearing all variables.

rm(list=ls())

Setting options.

options(stringsAsFactors = FALSE)

SouRcing

source("H:/JProg/R_prog/Basic_Functions.R")
source("H:/JProg/R_prog/Working_With_Arrays.R")
source("H:/JProg/R_prog/Working_With_GEO.R")

Loading libraries.

library("Hmisc")
library("GEOquery")
library("limma")
library("lumi")
library("xtable")
library("cluster")
library("sva")
library("GEOquery")

Setting working directory.

Create_Structure("/Dir/","GSE28475")
setwd("Dir/GSE28475/")
getwd()

Getting phenoData from soft file.

GSE28475 <- getGEO(filename="./Data/GSE28475_family.soft.gz")
platforms <- unlist(lapply(GPLList(GSE28475),function(x) Meta(x)$geo_accession))
pData_GSE28475 <- get_pData_table_general(GSE28475,platforms[[1]])
pData_GSE28475$pCh_sample_name <- gsub(" ","_",unlist(lapply(strsplit(pData_GSE28475$title,"_"),function(x) x[[length(x)]])))
rownames(pData_GSE28475) <- pData_GSE28475$geo_accession

Adding supplementary table information.

table_1 <- read.csv("H:/AutAndCanDef/Data/ASD_2/GSE28475/PaperNotesTables/Table1.csv",sep="\t")
table_2 <- read.csv("H:/AutAndCanDef/Data/ASD_2/GSE28475/PaperNotesTables/Table2.csv",sep="\t")
table_2$Sample_ID_number <- as.character(unlist(lapply(strsplit(table_2$Sample_ID,"[ABC]"),function(x) x[[1]])))
table_2$Sample_ID <- gsub(" ","_",table_2$Sample_ID)
table_2$Sample_ID_letter <- gsub("_rep","",unlist(lapply(strsplit(table_2$Sample_ID,"[[:digit:]]"),function(x) x[length(x)])))
Sample_ID_rep <- gsub("_","",gsub("[ABC_]","",unlist(lapply(strsplit(table_2$Sample_ID,"_"),function(x) x[length(x)]))))
Sample_ID_rep[!grepl("rep",Sample_ID_rep)] <- NA
table_2$Sample_ID_rep <- Sample_ID_rep
merged_tables <- merge(table_1,table_2,by.x = "Sample.ID",by.y="Sample_ID_number",all = T)
colnames(merged_tables) <- c("pCh_Sample.ID","pCh_Case.ID","pCh_Diagnosis","pCh_Age","pCh_Gender","pCh_COD","pCh_PMI","pCh_RIN_1","pCh_RIN_2","pCh_RIN_3","pCh_Sample_And_Rep","pCh_Sentrix_ID","pCh_Sentrix_position","pCh_Batch","pCh_Sample_ID_letter","pCh_Sample_ID_rep")
merged_tables$pCh_RIN_1 <- as.character(as.numeric(merged_tables$pCh_RIN_1))
merged_tables$pCh_RIN_2 <- as.character(as.numeric(merged_tables$pCh_RIN_2))
merged_tables$pCh_RIN_3 <- as.character(as.numeric(merged_tables$pCh_RIN_3))
merged_tables
merged_tables$pCh_Brain_Region <- rep("FCTX",nrow(merged_tables))
head(merged_tables)
merged_tables$pCh_Broadman_Area <- rep("BA9/46",nrow(merged_tables))
View(merged_tables)

Loading table to convert IDs

conversion <- read.csv(file = "H:/AutAndCanDef/Data/ASD_2/GSE28475/PaperNotesTables/Conversion.csv",sep="\t")
conversion$Old.Brain.Bank.ID <- gsub("-","",conversion$Old.Brain.Bank.ID)
conversion$Sample.Code
new_IDs <- c()
for(i in 1:nrow(merged_tables)){
 print(i)
 if(merged_tables[i,"pCh_Case.ID"] %in% conversion$Old.Brain.Bank.ID){
 new_ID <- conversion[conversion$Old.Brain.Bank.ID == merged_tables[i,"pCh_Case.ID"],"Sample.Code"]
 new_IDs <- c(new_IDs,new_ID)
 }
 else{
 new_IDs <- c(new_IDs,merged_tables[i,"pCh_Case.ID"])
 }
}

merged_tables$new_IDs <- new_IDs
merged_tables$new_IDs

conversion_2 <- read.csv(file = "H:/AutAndCanDef/Data/ASD_2/GSE28475/PaperNotesTables/Conversion2.csv",sep=",")
Alternate <- c()
for(i in 1:nrow(conversion_2)){
 if(conversion_2[i,2] == ""){
 Alternate <- c(Alternate,conversion_2[i,1])
 }else{
 Alternate <- c(Alternate,conversion_2[i,2])
 }
}
conversion_2$Alternate.ID <- Alternate

new_IDs_2 <- c()
for(i in 1:nrow(merged_tables)){
 print(i)
 if(merged_tables$new_IDs[i] %in% conversion_2[,"Case.ID"]){
 new_IDs_2 <- c(new_IDs_2,conversion_2[conversion_2$Case.ID == merged_tables$new_IDs[i],"Alternate.ID"])
 }else{
 new_IDs_2 <- c(new_IDs_2,merged_tables$new_IDs[i])
 }
}

merged_tables$new_IDs_2 <- new_IDs_2

pData_GSE28475 <- merge(pData_GSE28475,merged_tables,by.x="pCh_sample_name",by.y="pCh_Sample_And_Rep",all = T)
rownames(pData_GSE28475) <- pData_GSE28475$geo_accession
new_IDs_2 <- pData_GSE28475$new_IDs_2
selected_temp <- pData_GSE28475$new_IDs_2 == "B6736"
selected_temp[is.na(selected_temp)] <- FALSE
new_IDs_2[selected_temp] <- "AN02456"
selected_temp <- pData_GSE28475$new_IDs_2 == "B6401"
selected_temp[is.na(selected_temp)] <- FALSE
new_IDs_2[selected_temp] <- "AN06420"
pData_GSE28475$new_IDs_2 <- new_IDs_2
pData_GSE28475_filt <- pData_GSE28475[,c("pCh_sample_name","pCh_Broadman_Area","pCh_Brain_Region","new_IDs","new_IDs_2","pCh_Batch","pCh_Age","pCh_PMI","pCh_Gender","pCh_COD","pCh_Diagnosis","pCh_tissue_preservation")]
pData_GSE28475_filt$pCh_Study <- rep("GSE28475",nrow(pData_GSE28475_filt))
colnames(pData_GSE28475_filt)[1] <- "pCh_Sample_Name"
colnames(pData_GSE28475_filt)[5] <- "pCh_Individual"

Getting expression data

eData_GSE28475 <- get_eData(GSE28475,platforms[1])

Creating ExpressionSet

pData_GSE28475_filt <- pData_GSE28475_filt[colnames(eData_GSE28475),]
pheno <- as(pData_GSE28475_filt,"AnnotatedDataFrame")
intersect(rownames(pheno),colnames(eData_GSE28475))
GSE28475_Eset_Raw <- new('ExpressionSet',exprs=as.matrix(eData_GSE28475),phenoData=pheno)

Saving the raw data.

save(file = "./Data/GSE28475_Eset_Raw.Rda",GSE28475_Eset_Raw)
GSE28475_Eset_Raw <- get(load(file = "./Data/GSE28475_Eset_Raw.Rda"))

Generating quality plots before preprocessing.

pdf(file = "./Results/density_Pre_Norm.pdf")
lumi::density(GSE28475_Eset_Raw,legend = FALSE)
dev.off()
pdf(file = "./Results/box_Pre_Norm.pdf")
lumi::boxplot(GSE28475_Eset_Raw)
dev.off()

pn <- pData_to_pData_n(pData(GSE28475_Eset_Raw))
pdf(file = "./Results/PCA_Pre_Norm_Status.pdf")
plotPCA(GSE28475_Eset_Raw,groups = pn$pCh_Diagnosis,groupnames = unique(pData(GSE28475_Eset_Raw)$pCh_Diagnosis),legend = T)
dev.off()

group_batch <- paste(pData(GSE28475_Eset_Raw)$pCh_Diagnosis,"_Batch_",pData(GSE28475_Eset_Raw)$pCh_Batch,sep="")
group_batch
group_batch_num <- as.numeric(factor(group_batch))
group_batch_num
pdf(file = "./Results/PCA_Pre_Norm_Status_Batch.pdf")
affycoretools::plotPCA(GSE28475_Eset_Raw,groups =group_batch_num,groupnames = levels(factor(unique(group_batch))),legend = T)
dev.off()

IAC=cor(exprs(GSE28475_Eset_Raw),use="p")
pdf(file = "./Results/IAC_Hist_Pre_Norm.pdf")
hist(IAC,sub=paste("Mean=",format(mean(IAC[upper.tri(IAC)]),digits=3)),breaks=100)
dev.off()

cluster1=hclust(as.dist(1-IAC),method="average")
pdf(file = "./Results/IAC_Clust_Pre_Norm.pdf")
plot(cluster1,cex=0.7,labels=dimnames(GSE28475_Eset_Raw)[[2]])
dev.off()

meanIAC=apply(IAC,2,mean)
sdCorr=sd(meanIAC)
numbersd=(meanIAC-mean(meanIAC))/sdCorr
pdf(file = "./Results/IAC_Dev_From_Mean_IAC_Pre_Norm.pdf")
plot(numbersd)
abline(h=-2)
dev.off()

Filtering data. First, we only kept those samples that were preserved frozen. Then we performed mean IAC based outlier removal.

bool_filt <- grepl("Autism",pData(GSE28475_Eset_Raw)$pCh_Diagnosis) | grepl("Control",pData(GSE28475_Eset_Raw)$pCh_Diagnosis) & grepl("Frozen",pData(GSE28475_Eset_Raw)$pCh_tissue_preservation)
GSE28475_Eset_Raw <- GSE28475_Eset_Raw[,bool_filt]

group <- paste(pData(GSE28475_Eset_Raw)$pCh_Diagnosis,pData(GSE28475_Eset_Raw)$pCh_Batch)
GSE28475_Eset_Raw_test <- SR_IAC_Eset(GSE28475_Eset_Raw,group,0.9)
GSE28475_Eset_Raw <- SR_IAC_Eset(GSE28475_Eset_Raw,group,0.9)

Generating quality plots after mean IAC based outlier removal.

pdf(file = "./Results/density_Pre_2_Norm.pdf")
lumi::density(GSE28475_Eset_Raw,legend = FALSE)
dev.off()

pdf(file = "./Results/box_Pre_2_Norm.pdf")
lumi::boxplot(GSE28475_Eset_Raw)
dev.off()

pdf(file = "./Results/PCA_Pre_2_Norm_Status.pdf")
plotPCA(GSE28475_Eset_Raw,groups = pn$pCh_Diagnosis,groupnames = unique(pData(GSE28475_Eset_Raw)$pCh_Diagnosis),legend = T)
dev.off()

group_batch <- paste(pData(GSE28475_Eset_Raw)$pCh_Diagnosis,"_Batch_",pData(GSE28475_Eset_Raw)$pCh_Batch,sep="")
group_batch
group_batch_num <- as.numeric(factor(group_batch))
pdf(file = "./Results/PCA_Pre_2_Norm_Status_Batch.pdf")
affycoretools::plotPCA(GSE28475_Eset_Raw,groups =group_batch_num,groupnames = levels(factor(unique(group_batch))),legend = T)
dev.off()

IAC=cor(exprs(GSE28475_Eset_Raw),use="p")
pdf(file = "./Results/IAC_Hist_Pre_2_Norm.pdf")
hist(IAC,sub=paste("Mean=",format(mean(IAC[upper.tri(IAC)]),digits=3)),breaks=100)
dev.off()

cluster1=hclust(as.dist(1-IAC),method="average")
pdf(file = "./Results/IAC_Clust_Pre_2_Norm.pdf")
plot(cluster1,cex=0.7,labels=dimnames(GSE28475_Eset_Raw)[[2]])
dev.off()

meanIAC=apply(IAC,2,mean)
sdCorr=sd(meanIAC)
numbersd=(meanIAC-mean(meanIAC))/sdCorr
pdf(file = "./Results/IAC_Dev_From_Mean_IAC_Pre_2_Norm.pdf")
plot(numbersd)
abline(h=-2)
dev.off()

Saving dat.

save(file = "./Data/GSE28475_Eset_Raw_menIAC_Not_Froz.Rda",GSE28475_Eset_Raw)
GSE28475_Eset_Raw <- get(load(file = "./Data/GSE28475_Eset_Raw_menIAC_Not_Froz.Rda"))
GSE28475_FCTX_Eset_Raw <- GSE28475_Eset_Raw

Adding extra control samples

GSE36192_FCTX_Eset_Raw <- get(load(file = "/Dir/GSE36192/Data/GSE36192_FCTX_Eset_Raw.Rda"))

pheno_Samp <- pData(GSE36192_FCTX_Eset_Raw)
pheno_Samp$pCh_Age <- as.numeric(pheno_Samp$pCh_Age)
list_conv <- list()
list_conv[[2]] <- 18
list_conv_sub <- list()
list_conv_sub[["F"]] <- as.numeric(table(pData(GSE28475_FCTX_Eset_Raw)$pCh_Gender[pData(GSE28475_FCTX_Eset_Raw)$pCh_Diagnosis == "Autism"])/sum(table(pData(GSE28475_FCTX_Eset_Raw)$pCh_Gender[pData(GSE28475_FCTX_Eset_Raw)$pCh_Diagnosis == "Autism"])))[1]
list_conv_sub[["M"]] <- as.numeric(table(pData(GSE28475_FCTX_Eset_Raw)$pCh_Gender[pData(GSE28475_FCTX_Eset_Raw)$pCh_Diagnosis == "Autism"])/sum(table(pData(GSE28475_FCTX_Eset_Raw)$pCh_Gender[pData(GSE28475_FCTX_Eset_Raw)$pCh_Diagnosis == "Autism"])))[2]
list_conv[[1]] <- list_conv_sub

source("H:/JProg/R_prog/Basic_Functions.R")
source("H:/JProg/R_prog/Working_With_Arrays.R")
source("H:/JProg/R_prog/Working_With_GEO.R")

out_test <- optimum_n_selector_2(pheno_Samp,cols = c(2,3),list_conv,number_of_rep=200)

vec_vals <- c(mean(pData(GSE28475_Eset_Raw)$pCh_Age[pData(GSE28475_FCTX_Eset_Raw)$pCh_Diagnosis == "Autism"]),as.numeric(table(pData(GSE28475_Eset_Raw)$pCh_Gender[pData(GSE28475_FCTX_Eset_Raw)$pCh_Diagnosis == "Autism"])/sum(table(pData(GSE28475_Eset_Raw)$pCh_Gender[pData(GSE28475_FCTX_Eset_Raw)$pCh_Diagnosis == "Autism"]))))

out_test_filt <- out_test[[1]]
out_test_filt <- out_test_filt[(out_test_filt$vec_mean < list_conv[[2]] + 1) & ((out_test_filt[,4] < (list_conv[[1]][[1]] + 0.01)) &(out_test_filt[,4] > (list_conv[[1]][[1]] - 0.01))),]
out_test_filt_40 <- out_test_filt[out_test_filt$vec_n == 40,]
selelected_sample <- sample(rownames(out_test_filt_40),1)
sample_temp_vec <- out_test[[2]][[7947]]
GSE36192_FCTX_Eset_Raw_Sampled <- GSE36192_FCTX_Eset_Raw[,sample_temp_vec]

pData(GSE36192_FCTX_Eset_Raw_Sampled)$pCh_Diagnosis <- rep("Control",nrow(pData(GSE36192_FCTX_Eset_Raw_Sampled)))
common_rows <- intersect(rownames(GSE36192_FCTX_Eset_Raw_Sampled),rownames(GSE28475_FCTX_Eset_Raw))
GSE36192_FCTX_Eset_Raw_Sampled_Common_Rows <- GSE36192_FCTX_Eset_Raw_Sampled[common_rows,]
GSE28475_FCTX_Eset_Raw_Common_Rows <- GSE28475_FCTX_Eset_Raw[common_rows,]
GSE28475_Plus_GSE36192_FCTX_Eset_Raw <- cbind(exprs(GSE36192_FCTX_Eset_Raw_Sampled_Common_Rows),exprs(GSE28475_FCTX_Eset_Raw_Common_Rows))
GSE28475_Plus_GSE36192_FCTX_Eset_Raw_pheno <-
rbind.fill(pData(GSE36192_FCTX_Eset_Raw_Sampled_Common_Rows),pData(GSE28475_FCTX_Eset_Raw_Common_Rows))
rownames(GSE28475_Plus_GSE36192_FCTX_Eset_Raw_pheno) <- c(rownames(pData(GSE36192_FCTX_Eset_Raw_Sampled_Common_Rows)),rownames(pData(GSE28475_FCTX_Eset_Raw_Common_Rows)))
pheno <- as(GSE28475_Plus_GSE36192_FCTX_Eset_Raw_pheno,"AnnotatedDataFrame")
GSE28475_Plus_GSE36192_FCTX_Eset_Raw <- new('ExpressionSet',exprs=as.matrix(GSE28475_Plus_GSE36192_FCTX_Eset_Raw),phenoData=pheno)
dim(GSE28475_Plus_GSE36192_FCTX_Eset_Raw)
pCh_Individual <- pData(GSE28475_Plus_GSE36192_FCTX_Eset_Raw)$pCh_Individual
pCh_new_IDs_2 <- pData(GSE28475_Plus_GSE36192_FCTX_Eset_Raw)$new_IDs_2
pData(GSE28475_Plus_GSE36192_FCTX_Eset_Raw)$pCh_Individual <- pCh_Individual
head(pData(GSE28475_Plus_GSE36192_FCTX_Eset_Raw))
pData(GSE28475_Plus_GSE36192_FCTX_Eset_Raw)$pCh_Batch
pData(GSE28475_Plus_GSE36192_FCTX_Eset_Raw)$pCh_Batch[1:40] <- "3"
pData(GSE28475_Plus_GSE36192_FCTX_Eset_Raw)$pCh_Batch <- paste("Batch_",pData(GSE28475_Plus_GSE36192_FCTX_Eset_Raw)$pCh_Batch,sep="")
View(pData(GSE28475_Plus_GSE36192_FCTX_Eset_Raw))

Saving samples not sampled from GSE36192

GSE36192_FCTX_Eset_Raw_Not_Sampled <- GSE36192_FCTX_Eset_Raw[,!colnames(GSE36192_FCTX_Eset_Raw) %in% colnames(GSE28475_Plus_GSE36192_FCTX_Eset_Raw)]
GSE36192_FCTX_Eset_Raw_Not_Sampled
save(file = "H:/AutAndCanDef/Data/ASD_2/Auxiliar_Data/GSE36192/Data/GSE36192_FCTX_Eset_Raw_Not_Sampled.Rda",GSE36192_FCTX_Eset_Raw_Not_Sampled)

Saving data.

GSE28475_Plus_GSE36192_FCTX_Eset_Raw
setwd("H:/AutAndCanDef/Data/ASD_2/GSE28475/")
save(file = "./Data/GSE28475_Plus_GSE36192_FCTX_Eset_Raw.Rda",GSE28475_Plus_GSE36192_FCTX_Eset_Raw)
t.test(as.numeric(pData(GSE28475_Plus_GSE36192_FCTX_Eset_Raw)$pCh_Age) ~ pData(GSE28475_Plus_GSE36192_FCTX_Eset_Raw)$pCh_Diagnosis)
tbl <- table(pData(GSE28475_Plus_GSE36192_FCTX_Eset_Raw)$pCh_Gender,pData(GSE28475_Plus_GSE36192_FCTX_Eset_Raw)$pCh_Diagnosis)
GSE28475_Plus_GSE36192_FCTX_Eset_Raw <- get(load(file = "./Data/GSE28475_Plus_GSE36192_FCTX_Eset_Raw.Rda"))

GSE28475_Plus_GSE36192_FCTX_Eset_Raw_bgc <- backgroundCorrect(exprs(GSE28475_Plus_GSE36192_FCTX_Eset_Raw), method="normexp",normexp.method = "rma",offset = 50)

GSE28475_Plus_GSE36192_FCTX_Eset_Raw_bgc_norm <- normalizeBetweenArrays(GSE28475_Plus_GSE36192_FCTX_Eset_Raw_bgc, method="quantile")

GSE28475_Plus_GSE36192_FCTX_Eset_Raw_bgc_norm_log2 <- log2(GSE28475_Plus_GSE36192_FCTX_Eset_Raw_bgc_norm)

GSE28475_Plus_GSE36192_FCTX_Eset_Proc <- GSE28475_Plus_GSE36192_FCTX_Eset_Raw
exprs(GSE28475_Plus_GSE36192_FCTX_Eset_Proc) <- GSE28475_Plus_GSE36192_FCTX_Eset_Raw_bgc_norm_log2

Generating quality plots after normalization.

pdf(file = "./Results/density_FCTX_Post_Norm.pdf")
lumi::density(GSE28475_Plus_GSE36192_FCTX_Eset_Proc,legend = FALSE,logMode=TRUE)
dev.off()

pdf(file = "./Results/box_FCTX_Post_Norm.pdf")
lumi::boxplot(GSE28475_Plus_GSE36192_FCTX_Eset_Proc)
dev.off()
pn <- pData_to_pData_n(pData(GSE28475_Plus_GSE36192_FCTX_Eset_Proc))
head(pn)

pdf(file = "./Results/PCA_FCTX_Post_Norm_Status.pdf")
groups = pn$pCh_Diagnosis
groups <- pData(GSE28475_Plus_GSE36192_FCTX_Eset_Proc)$pCh_Diagnosis
plotPCA(GSE28475_Plus_GSE36192_FCTX_Eset_Proc,groups = pn$pCh_Diagnosis,groupnames = levels(factor(groups)),legend = T)
dev.off()

pdf(file = "./Results/PCA_FCTX_Post_Norm_Batch.pdf")
#groups = paste(pn$pCh_Batch,pn$pCh_Diagnosis)
groups = as.numeric(as.factor(groups))
groups[is.na(groups)] <- 2
group_names <- paste(pData(GSE28475_Plus_GSE36192_FCTX_Eset_Proc)$pCh_Batch,pData(GSE28475_Plus_GSE36192_FCTX_Eset_Proc)$pCh_Diagnosis)
plotPCA(GSE28475_Plus_GSE36192_FCTX_Eset_Proc,groups = groups,groupnames = levels(factor(group_names)),legend = T)
dev.off()

IAC=cor(exprs(GSE28475_Plus_GSE36192_FCTX_Eset_Proc),use="p")
pdf(file = "./Results/IAC_FCTX_Hist_Post_Norm.pdf")
hist(IAC,sub=paste("Mean=",format(mean(IAC[upper.tri(IAC)]),digits=3)),breaks=100)
dev.off()

cluster1=hclust(as.dist(1-IAC),method="average")
pdf(file = "./Results/IAC_FCTX_Clust_Post_Norm.pdf")
plot(cluster1,cex=0.7,labels=dimnames(GSE28475_Plus_GSE36192_FCTX_Eset_Proc)[[2]])
dev.off()

meanIAC=apply(IAC,2,mean)
sdCorr=sd(meanIAC)
numbersd=(meanIAC-mean(meanIAC))/sdCorr
pdf(file = "./Results/IAC_FCTX_Dev_From_Mean_IAC_Post_Norm.pdf")
plot(numbersd)
abline(h=-2)
dev.off()

Batch effect correction

library(sva)
batch = pData(GSE28475_Plus_GSE36192_FCTX_Eset_Proc)$pCh_Batch
pData(GSE28475_Plus_GSE36192_FCTX_Eset_Proc)$pCh_Diagnosis
modcombat = model.matrix(~as.factor(pCh_Diagnosis), data=pData(GSE28475_Plus_GSE36192_FCTX_Eset_Proc))
modcombat
combat_edata = ComBat(dat=exprs(GSE28475_Plus_GSE36192_FCTX_Eset_Proc), batch=batch, mod=modcombat, par.prior=TRUE, prior.plots=FALSE)
exprs(GSE28475_Plus_GSE36192_FCTX_Eset_Proc) <- combat_edata

Collapse the technical replicates.

samp_ID_char <- paste(pData(GSE28475_Plus_GSE36192_FCTX_Eset_Proc)$pCh_Individual,pData(GSE28475_Plus_GSE36192_FCTX_Eset_Proc)$pCh_Diagnosis,sep="_")
samp_ID_char[duplicated(samp_ID_char)]
GSE28475_Plus_GSE36192_FCTX_Eset_Proc <- Combine_Replicates(GSE28475_Plus_GSE36192_FCTX_Eset_Proc,samp_ID_char)
GSE28475_Plus_GSE36192_FCTX_Eset_Proc <- recode_pData_After_Merging(GSE28475_Plus_GSE36192_FCTX_Eset_Proc)

Generating quality plots after batch effect correction and averaging technical replicates.

pdf(file = "./Results/density_FCTX_Post_Batch_Mean_Norm.pdf")
lumi::density(GSE28475_Plus_GSE36192_FCTX_Eset_Proc,legend = FALSE,logMode=TRUE)
dev.off()

pdf(file = "./Results/box_FCTX_Post_Batch_Mean_Norm.pdf")
lumi::boxplot(GSE28475_Plus_GSE36192_FCTX_Eset_Proc)
dev.off()
pn <- pData_to_pData_n(pData(GSE28475_Plus_GSE36192_FCTX_Eset_Proc))

pdf(file = "./Results/PCA_FCTX_Post_Batch_Mean_Norm_Status.pdf")
plotPCA(GSE28475_Plus_GSE36192_FCTX_Eset_Proc,groups = pn$pCh_Diagnosis,legend = FALSE)
dev.off()

IAC=cor(exprs(GSE28475_Plus_GSE36192_FCTX_Eset_Proc),use="p")
pdf(file = "./Results/IAC_FCTX_Hist_Post_Batch_Mean_Norm.pdf")
hist(IAC,sub=paste("Mean=",format(mean(IAC[upper.tri(IAC)]),digits=3)),breaks=100)
dev.off()

cluster1=hclust(as.dist(1-IAC),method="average")
pdf(file = "./Results/IAC_FCTX_Clust_Post_Batch_Mean_Norm.pdf")
plot(cluster1,cex=0.7,labels=dimnames(GSE28475_Plus_GSE36192_FCTX_Eset_Proc)[[2]])
dev.off()

meanIAC=apply(IAC,2,mean)
sdCorr=sd(meanIAC)
numbersd=(meanIAC-mean(meanIAC))/sdCorr

pdf(file = "./Results/IAC_FCTX_Dev_From_Mean_IAC_Post_Batch_Mean_Norm.pdf")
plot(numbersd)
abline(h=-2)
dev.off()

Saving the preprocessed data

save(file = "./Data/GSE28475_Plus_GSE36192_FCTX_Eset_Proc.Rda",GSE28475_Plus_GSE36192_FCTX_Eset_Proc)
GSE28475_Plus_GSE36192_FCTX_Eset_Proc <- get(load(file = "./Data/GSE28475_Plus_GSE36192_FCTX_Eset_Proc.Rda"))

Preparing for meta-analysis

library("illuminaHumanv3.db")

x <- illuminaHumanv3ENTREZID

GSE28475_FCTX_coll_MaxMean <- collapse_rows(x,GSE28475_Plus_GSE36192_FCTX_Eset_Proc,Method = "MaxMean")

GSE28475_FCTX_coll_Average <- collapse_rows(x,GSE28475_Plus_GSE36192_FCTX_Eset_Proc,Method = "Average")

GSE28475_FCTX_coll_MaxRowVar <- collapse_rows(x,GSE28475_Plus_GSE36192_FCTX_Eset_Proc,"maxRowVariance")

factor <- pData(GSE28475_Plus_GSE36192_FCTX_Eset_Proc)$pCh_Diagnosis
factor[factor == "Autism"] <- 1
factor[factor == "Control"] <- 0

GSE28475_FCTX_MM <- list(GSE28475_FCTX_coll_MaxMean,factor)
GSE28475_FCTX_AV <- list(GSE28475_FCTX_coll_Average,factor)
GSE28475_FCTX_MRV <- list(GSE28475_FCTX_coll_MaxRowVar,factor)

save(file="./Data/GSE28475_FCTX_MM.Rda",GSE28475_FCTX_MM)
save(file="./Data/GSE28475_FCTX_AV.Rda",GSE28475_FCTX_AV)
save(file="./Data/GSE28475_FCTX_MRV.Rda",GSE28475_FCTX_MRV)

AI.3) Gupta’s RNAseq dataset preprocessing

Clearing all variables and collecting garbage

rm(list=ls())
gc()

options(stringsAsFactors = F)

SouRcing

source("H:/JProg/R_prog/Basic_Functions.R")
source("H:/JProg/R_prog/Working_With_Arrays.R")
source("H:/JProg/R_prog/Working_With_GEO.R")

library("EDASeq")
library("DESeq2")
install.packages("lazyeval")

Create_Structure("/Dir/","ASD_RNAseq_A")
setwd("Dir/ASD_RNAseq_A/")
getwd()

Recoding phenotipic data and reading RNAseq expression files

ASD <- read.csv2(file = "Dir/Data/genetable.txt",sep="\t",row.names = 1,header = T)

for(i in 1:ncol(ASD)){
 ASD[,i] <- as.numeric(as.character(ASD[,i]))
}

filter <- apply(ASD,1,function(x) mean(x)>5)

ASD_data <- ASD[filter,]

ASD_pheno <- read.csv2(file = "H:/AutAndCanDef/Data/ASD_2/ASD_RNAseq_A/Data/Samples104.EDASeqFullBrainFeatures",sep="\t",row.names = 1,header = T)

ASD_pheno <- ASD_pheno[,!m_grepl(c("PC","ISV"),colnames(ASD_pheno))]

intersected <- intersect(colnames(ASD_data), rownames(ASD_pheno))

ASD_data <-ASD_data[,intersected]
ASD_pheno <- ASD_pheno[intersected,]

pCh_Broadman_Area <- ASD_pheno$ID
pCh_Broadman_Area[grepl("ba19",pCh_Broadman_Area)] <- "BA19"
pCh_Broadman_Area[grepl("ba10",pCh_Broadman_Area)] <- "BA10"
pCh_Broadman_Area[grepl("ba44",pCh_Broadman_Area)] <- "BA44"

pCh_Brain_Region <- pCh_Broadman_Area
pCh_Brain_Region[grepl("BA19",pCh_Brain_Region)] <- "OCTX"
pCh_Brain_Region[grepl("BA10",pCh_Brain_Region)] <- "FCTX"
pCh_Brain_Region[grepl("BA44",pCh_Brain_Region)] <- "FCTX"

pCh_RIN <- ASD_pheno$RIN

pCh_PMI <- trimws(ASD_pheno$PMI)

pCh_Gender <- ASD_pheno$Gender

pCh_Age <- ASD_pheno$Age

pCh_Individual <- ASD_pheno$case

pCh_Diagnosis <- ASD_pheno$Dx

pCh_Rownames <- rownames(ASD_pheno)

names_for_rows <- rownames(ASD_pheno)

ASD_pheno <- data.frame(pCh_Rownames,pCh_Individual,pCh_Diagnosis,pCh_Gender,pCh_Age,pCh_PMI,pCh_RIN,pCh_Broadman_Area,pCh_Brain_Region_Description,pCh_Brain_Region)
rownames(ASD_pheno) <- names_for_rows


ASD_pheno_2 <- read.csv2(file = "/Dir/Data/Pheno_Suppl.csv",sep="\t",row.names = 1,header = T)

ASD_pheno_merged <- merge(ASD_pheno,ASD_pheno_2,by.x = "pCh_Individual",by.y="Old.Brain.Bank.ID",all.x = TRUE)


pCh_Brain_Bank <- ASD_pheno_merged$Site

pCh_Individual_2 <- ASD_pheno_merged$Sample.Code

pCh_COD <- ASD_pheno_merged$Cause.of.Death..primary.

pCh_Ethnicity <- ASD_pheno_merged$Ethinicity

ASD_pheno_merged$pCh_Brain_Bank <- pCh_Brain_Bank

ASD_pheno_merged$pCh_Individual_2 <- pCh_Individual_2

ASD_pheno_merged$pCh_COD <- pCh_COD

ASD_pheno_merged$pCh_Ethnicity <- pCh_Ethnicity

ASD_pheno_merged <- ASD_pheno_merged[,grepl("pCh",colnames(ASD_pheno_merged))]

rownames(ASD_pheno_merged) <- ASD_pheno_merged$pCh_Rownames

intersected <- intersect(colnames(ASD_data), rownames(ASD_pheno_merged))

ASD_data <- ASD_data[,intersected]

ASD_pheno_merged <- ASD_pheno_merged[intersected,]


ASD_GC_GL <- read.csv2(file = "/Dir/Data/P10.70.19.GC.GeneLength",sep="\t",row.names = 1,header = T)
head(ASD_GC_GL)

ASD_GC_GL[,1] <- as.numeric(ASD_GC_GL[,1])
ASD_GC_GL[,2] <- as.numeric(ASD_GC_GL[,2])

intersected <- intersect(rownames(ASD_GC_GL),rownames(ASD_data))

ASD_data <- ASD_data[intersected,]

ASD_GC_GL <- ASD_GC_GL[intersected,]

colnames(ASD_data) <- gsub("\\.","_",colnames(ASD_data))
rownames(ASD_data) <- gsub("\\..*","",rownames(ASD_data))
rownames(ASD_pheno_merged) <- gsub("\\.","_",rownames(ASD_pheno_merged))
colnames(ASD_GC_GL) <- gsub("\\.","_",colnames(ASD_GC_GL))
rownames(ASD_GC_GL) <- gsub("\\..*","",rownames(ASD_GC_GL))
ASD_data <- newSeqExpressionSet(counts=as.matrix(ASD_data),phenoData=ASD_pheno_merged,featureData=ASD_GC_GL)

Rlog normalizing function

rlog_norm <- function(data){
 pheno <- pData(data)
 pFac_Diagnosis <- factor(pheno$pCh_Diagnosis)
 pFac_Diagnosis <- relevel(pFac_Diagnosis, ref = "Control")
 pheno$pFac_Diagnosis <- pFac_Diagnosis
 dds <- DESeqDataSetFromMatrix(countData = counts(data)+1,colData = pheno,design = ~ pFac_Diagnosis)
 rld <- rlog(dds, blind=FALSE)
 phenoData <- new("AnnotatedDataFrame",data=pData(data))
 eset <- ExpressionSet(assayData=assay(rld),phenoData=phenoData)
 exprs(eset)[exprs(eset) < 0] <- 0.1
 return(eset)
}

Selecting frontal cortex data and performing rlog transformation.

library("WGCNA")
library("org.Hs.eg.db")

ASD_data_FCTX <- ASD_data[,pData(ASD_data)$pCh_Brain_Region == "FCTX"]

ASD_data_FCTX_Norm <- rlog_norm(ASD_data_FCTX)

Generating quality plots

pdf(file = "H:/AutAndCanDef/Data/ASD_2/ASD_RNAseq_A/Results_3/Densy_Box_ASD_data_FCTX_Norm.pdf")
par(mfrow=c(1,2))
boxplot(ASD_data_FCTX_Norm,log =F)
density(ASD_data_FCTX_Norm,log = F)
dev.off()

Saving data

save(file="Dir/Data/ASD_data_FCTX_Norm_rlog.Rda",ASD_data_FCTX_Norm)

ASD_data_FCTX_Norm <- get(load(file="Dir/Data/ASD_data_FCTX_Norm_rlog.Rda"))

Preparing for meta-analysis

xx <- as.list(org.Hs.egENSEMBL2EG)
out_list <- xx[rownames(ASD_data_FCTX_Norm)]
df_entrez <- data.frame(names(unlist(out_list)),unlist(out_list))

ASD_data_FCTX_Norm <- ASD_data_FCTX_Norm[rownames(ASD_data_FCTX_Norm) %in% rownames(df_entrez),]

RNAseq_FCTX_coll_MaxMean <- collapse_rows_no_anot(df_entrez,ASD_data_FCTX_Norm,Method = "MaxMean")
RNAseq_FCTX_coll_Average <- collapse_rows_no_anot(df_entrez,ASD_data_FCTX_Norm,Method = "Average")
RNAseq_FCTX_coll_MaxRowVar <- collapse_rows_no_anot(df_entrez,ASD_data_FCTX_Norm,Method = "maxRowVariance")

factor <- pData(ASD_data_FCTX_Norm)$pCh_Diagnosis
factor[grepl("Autism",factor)] <- 1
factor[grepl("Control",factor)] <- 0

RNAseq_FCTX_MM <- list(RNAseq_FCTX_coll_MaxMean,factor)
RNAseq_FCTX_AV <- list(RNAseq_FCTX_coll_Average,factor)
RNAseq_FCTX_MRV <- list(RNAseq_FCTX_coll_MaxRowVar,factor)

save(file="./Data/RNAseq_FCTX_rlog_New_MM.Rda",RNAseq_FCTX_MM)
save(file="./Data/RNAseq_FCTX_rlog_New_AV.Rda",RNAseq_FCTX_AV)
save(file="./Data/RNAseq_FCTX_rlog_New_MRV.Rda",RNAseq_FCTX_MRV)
save(file = "Dir/Data/RNAseq_FCTX_rlog_New_Eset_Proc.Rda",ASD_data_FCTX_Norm)

AI.4) ASD differential gene expression meta-analysis

rm(list=ls())
options(stringsAsFactors = FALSE)

library("imputeTS")
library("MetaDE")

GSE28521 <- get(load(file = "/Dir/Data/GSE28521_AND_GSE36192_MRV.Rda"))
IAC_GSE28521 = cor(GSE28521[[1]],use="p")
mean_IAC_GSE28521 <- mean(IAC_GSE28521)

ASD_RNAseq_FCTX_A <- get(load(file = "Dir/Data/RNAseq_FCTX_rlog_New_MRV.Rda"))
IAC_ASD_RNAseq_A = cor(ASD_RNAseq_FCTX_A[[1]],use="p")
mean_IAC_ASD_RNAseq_A <- mean(IAC_ASD_RNAseq_A)

GSE28475 <- get(load(file = "Dir/Data/GSE28475_AND_GSE36192_MRV.Rda"))
IAC_GSE28475 = cor(GSE28475[[1]],use="p")
mean_IAC_GSE28475 <- mean(IAC_GSE28475)

Removing redundant samples given MetaQC and meanIAC quality ordering.

GSE28521_Eset_Proc <- get(load(file = "/Dir/Data/GSE28521_AND_GSE36192_FCTX_Eset_Proc.Rda"))

RNAseq_A_FCTX_New_Eset_Proc <- get(load(file = "/Dir/Data/RNAseq_FCTX_rlog_New_Eset_Proc.Rda"))

GSE28475_Eset_Proc <- get(load(file = "/Dir/Data/GSE28475_AND_GSE36192_FCTX_Eset_Proc.Rda"))


p_GSE28521 <- pData(GSE28521_Eset_Proc)
df_p_GSE28521 <- data.frame(p_GSE28521$pCh_Patient_ID,p_GSE28521$pCh_Diagnosis,rep("GSE28521",nrow(p_GSE28521)))
colnames(df_p_GSE28521) <- c("ID","STATUS","STUDY")
p_RNAseq <- pData(RNAseq_A_FCTX_New_Eset_Proc)
df_p_RNAseq <- data.frame(p_RNAseq$pCh_Individual_2,p_RNAseq$pCh_Diagnosis,rep("RNAseq",nrow(p_RNAseq)))
colnames(df_p_RNAseq) <- c("ID","STATUS","STUDY")
p_GSE28475 <- pData(GSE28475_Eset_Proc)
df_p_GSE28475 <- data.frame(p_GSE28475$pCh_Individual,p_GSE28475$pCh_Diagnosis,rep("RNAseq",nrow(p_GSE28475)))
colnames(df_p_GSE28475) <- c("ID","STATUS","STUDY")
df_all <- rbind(df_p_GSE28521,df_p_RNAseq,df_p_GSE28475)


to_select_in_B <- setdiff(RNAseq_A_FCTX_New_Eset_Proc$pCh_Individual_2,GSE28521_Eset_Proc$pCh_Patient_ID)
RNAseq_A_FCTX_New_Eset_Proc <- RNAseq_A_FCTX_New_Eset_Proc[,pData(RNAseq_A_FCTX_New_Eset_Proc)$pCh_Individual_2 %in% to_select_in_B]
RNAseq_A_FCTX_New_Eset_Proc <- RNAseq_A_FCTX_New_Eset_Proc[,!duplicated(RNAseq_A_FCTX_New_Eset_Proc$pCh_Individual_2)]

In_A_And_B <- c(GSE28521_Eset_Proc$pCh_Individual,RNAseq_A_FCTX_New_Eset_Proc$pCh_Individual_2)
In_C_Not_In_A_And_B <- setdiff(GSE28475_Eset_Proc$pCh_Individual,In_A_And_B)
GSE28475_Eset_Proc <- GSE28475_Eset_Proc[,GSE28475_Eset_Proc$pCh_Individual %in% In_C_Not_In_A_And_B]

Computing differences in covariate distributions between cases and controls.

GSE28521_Included_Pheno <- pData(GSE28521_Eset_Proc)[,c("pCh_Age","pCh_Gender","pCh_PMI","pCh_Individual","pCh_Diagnosis")]
table(GSE28521_Included_Pheno$pCh_Diagnosis)

#Gender distribution

a_gen <- table(GSE28521_Included_Pheno$pCh_Gender,GSE28521_Included_Pheno$pCh_Diagnosis)
b_gen <- round(prop.table(table(GSE28521_Included_Pheno$pCh_Gender,GSE28521_Included_Pheno$pCh_Diagnosis),2),digits = 2)
df_gen <- data.frame(matrix(paste(a_gen," (",b_gen,"%)",sep=""),ncol = 2))
colnames(df_gen) <- colnames(a_gen)
rownames(df_gen) <- rownames(b_gen)
chisq.test(GSE28521_Included_Pheno$pCh_Gender,GSE28521_Included_Pheno$pCh_Diagnosis)

#Age distribution

Autism_Mean_Age <- mean(as.numeric(GSE28521_Included_Pheno$pCh_Age)[GSE28521_Included_Pheno$pCh_Diagnosis == "Autism"])
Control_Mean_Age <- mean(as.numeric(GSE28521_Included_Pheno$pCh_Age)[GSE28521_Included_Pheno$pCh_Diagnosis == "Control"])
Autism_SD_Age <- sd(as.numeric(GSE28521_Included_Pheno$pCh_Age)[GSE28521_Included_Pheno$pCh_Diagnosis == "Autism"])
Control_SD_Age <- sd(as.numeric(GSE28521_Included_Pheno$pCh_Age)[GSE28521_Included_Pheno$pCh_Diagnosis == "Control"])
t.test(as.numeric(GSE28521_Included_Pheno$pCh_Age)~GSE28521_Included_Pheno$pCh_Diagnosis)

#PMI distribution

PMI <- na.interpolation(as.numeric(GSE28521_Included_Pheno$pCh_PMI))
Autism_Mean_PMI <- mean(PMI[GSE28521_Included_Pheno$pCh_Diagnosis == "Autism"])
Control_Mean_PMI <- mean(PMI[GSE28521_Included_Pheno$pCh_Diagnosis == "Control"])

Autism_SD_PMI <- sd(PMI[GSE28521_Included_Pheno$pCh_Diagnosis == "Autism"])
Control_SD_PMI <- sd(PMI[GSE28521_Included_Pheno$pCh_Diagnosis == "Control"])
t.test(PMI~GSE28521_Included_Pheno$pCh_Diagnosis)


RNAseq_Included_Pheno <- pData(RNAseq_A_FCTX_New_Eset_Proc)[,c("pCh_Age","pCh_Gender","pCh_PMI","pCh_Individual_2","pCh_Diagnosis")]
table(RNAseq_Included_Pheno$pCh_Diagnosis)


#Gender distribution

a_gen <- table(RNAseq_Included_Pheno$pCh_Gender,RNAseq_Included_Pheno$pCh_Diagnosis)
b_gen <- round(prop.table(table(RNAseq_Included_Pheno$pCh_Gender,RNAseq_Included_Pheno$pCh_Diagnosis),2),digits = 2)
df_gen <- data.frame(matrix(paste(a_gen," (",b_gen,"%)",sep=""),ncol = 2))
colnames(df_gen) <- colnames(a_gen)
rownames(df_gen) <- rownames(b_gen)
chisq.test(RNAseq_Included_Pheno$pCh_Gender,RNAseq_Included_Pheno$pCh_Diagnosis)

#Age distribution

Autism_Mean_Age <- mean(as.numeric(RNAseq_Included_Pheno$pCh_Age)[RNAseq_Included_Pheno$pCh_Diagnosis == "Autism"])
Control_Mean_Age <- mean(as.numeric(RNAseq_Included_Pheno$pCh_Age)[RNAseq_Included_Pheno$pCh_Diagnosis == "Control"])
Autism_SD_Age <- sd(as.numeric(RNAseq_Included_Pheno$pCh_Age)[RNAseq_Included_Pheno$pCh_Diagnosis == "Autism"])
Control_SD_Age <- sd(as.numeric(RNAseq_Included_Pheno$pCh_Age)[RNAseq_Included_Pheno$pCh_Diagnosis == "Control"])
t.test(as.numeric(RNAseq_Included_Pheno$pCh_Age)~RNAseq_Included_Pheno$pCh_Diagnosis)

#PMI distribution

PMI <- na.interpolation(as.numeric(RNAseq_Included_Pheno$pCh_PMI))
Autism_Mean_PMI <- mean(PMI[RNAseq_Included_Pheno$pCh_Diagnosis == "Autism"])
Control_Mean_PMI <- mean(PMI[RNAseq_Included_Pheno$pCh_Diagnosis == "Control"])

Autism_SD_PMI <- sd(PMI[RNAseq_Included_Pheno$pCh_Diagnosis == "Autism"])
Control_SD_PMI <- sd(PMI[RNAseq_Included_Pheno$pCh_Diagnosis == "Control"])
t.test(PMI~RNAseq_Included_Pheno$pCh_Diagnosis)


GSE28475_Included_Pheno <- pData(GSE28475_Eset_Proc)[,c("pCh_Age","pCh_Gender","pCh_PMI","pCh_Individual","pCh_Diagnosis")]
table(GSE28475_Included_Pheno$pCh_Diagnosis)

#Gender distribution

a_gen <- table(GSE28475_Included_Pheno$pCh_Gender,GSE28475_Included_Pheno$pCh_Diagnosis)
b_gen <- round(prop.table(table(GSE28475_Included_Pheno$pCh_Gender,GSE28475_Included_Pheno$pCh_Diagnosis),2),digits = 2)
df_gen <- data.frame(matrix(paste(a_gen," (",b_gen,"%)",sep=""),ncol = 2))
colnames(df_gen) <- colnames(a_gen)
rownames(df_gen) <- rownames(b_gen)
chisq.test(GSE28475_Included_Pheno$pCh_Gender,GSE28475_Included_Pheno$pCh_Diagnosis)

#Age distribution

Autism_Mean_Age <- mean(as.numeric(GSE28475_Included_Pheno$pCh_Age)[GSE28475_Included_Pheno$pCh_Diagnosis == "Autism"])
Control_Mean_Age <- mean(as.numeric(GSE28475_Included_Pheno$pCh_Age)[GSE28475_Included_Pheno$pCh_Diagnosis == "Control"])
Autism_SD_Age <- sd(as.numeric(GSE28475_Included_Pheno$pCh_Age)[GSE28475_Included_Pheno$pCh_Diagnosis == "Autism"])
Control_SD_Age <- sd(as.numeric(GSE28475_Included_Pheno$pCh_Age)[GSE28475_Included_Pheno$pCh_Diagnosis == "Control"])
t.test(as.numeric(GSE28475_Included_Pheno$pCh_Age)~GSE28475_Included_Pheno$pCh_Diagnosis)

#PMI distribution

PMI <- na.interpolation(as.numeric(GSE28475_Included_Pheno$pCh_PMI))
Autism_Mean_PMI <- mean(PMI[GSE28475_Included_Pheno$pCh_Diagnosis == "Autism"])
Control_Mean_PMI <- mean(PMI[GSE28475_Included_Pheno$pCh_Diagnosis == "Control"])

Autism_SD_PMI <- sd(PMI[GSE28475_Included_Pheno$pCh_Diagnosis == "Autism"])
Control_SD_PMI <- sd(PMI[GSE28475_Included_Pheno$pCh_Diagnosis == "Control"])
t.test(PMI~GSE28475_Included_Pheno$pCh_Diagnosis)


#Concatenating all data

colnames(RNAseq_Included_Pheno)[4] <- "pCh_Individual"
GSE28521_Included_Pheno$Dataset <- rep("GSE28521/GSE36192",nrow(GSE28521_Included_Pheno))
RNAseq_Included_Pheno$Dataset <- rep("Gupta",nrow(RNAseq_Included_Pheno))
GSE28475_Included_Pheno$Dataset <- rep("GSE28475/GSE36192",nrow(GSE28475_Included_Pheno))


All_data <- rbind(GSE28521_Included_Pheno,RNAseq_Included_Pheno,GSE28475_Included_Pheno)
t.test(as.numeric(All_data$pCh_Age)~All_data$pCh_Diagnosis)
t.test(as.numeric(All_data$pCh_PMI)~All_data$pCh_Diagnosis)
chisq.test(All_data$pCh_Gender,All_data$pCh_Diagnosis)

Performing differential gene expression meta-analysis.

#Creating the non-redundant samples dataset.

bool_RNSeq <- colnames(ASD_RNAseq_FCTX_A[[1]]) %in% rownames(RNAseq_Included_Pheno)
ASD_RNAseq_FCTX_A[[1]] <- ASD_RNAseq_FCTX_A[[1]][,bool_RNSeq]
ASD_RNAseq_FCTX_A[[2]] <- ASD_RNAseq_FCTX_A[[2]][bool_RNSeq]

bool_GSE28475 <- colnames(GSE28475[[1]]) %in% rownames(GSE28475_Included_Pheno)
GSE28475[[1]] <- GSE28475[[1]][,bool_GSE28475]
GSE28475[[2]] <- GSE28475[[2]][bool_GSE28475]

#Selecting only those genes included in the three datasets.

intersected <- intersect(intersect(rownames(GSE28521[[1]]),rownames(ASD_RNAseq_FCTX_A[[1]])),rownames(GSE28475[[1]]))

GSE28521[[1]] <- GSE28521[[1]][intersected,]
ASD_RNAseq_FCTX_A[[1]] <- ASD_RNAseq_FCTX_A[[1]][intersected,]
GSE28475[[1]] <- GSE28475[[1]][intersected,]

list_meta <- list(GSE28521,ASD_RNAseq_FCTX_A,GSE28475)

Meta_analysis

ASD_Meta <- ind.cal.ES(list_meta,paired = rep(FALSE,3),nperm = 1000)
ASD_Meta_Res <- MetaDE.ES(ASD_Meta,meta.method="REM")
save(file = "/Dir/Data/ASD_Meta.Rmd",ASD_Meta_Res)

AI.5) Functions

Remove samples from each group iteratively untial a mean IAC correlation value higer than 0.9 is reached.

SR_IAC_based <- function(eset,threshold = 0.9){
 IAC=cor(exprs(eset),use="p")
 IACmean <- mean(IAC[upper.tri(IAC)])
 meanIAC=apply(IAC,2,mean)
 sdCorr=sd(meanIAC)
 while(IACmean < threshold & ncol(eset) > 3){
 meanIAC=apply(IAC,2,mean)
 sdCorr=sd(meanIAC)
 numbersd=(meanIAC-mean(meanIAC))/sdCorr
 eset=eset[,-which.min(numbersd)]
 IAC=cor(exprs(eset),use="p")
 IACmean <- mean(IAC[upper.tri(IAC)])
}
 print(IACmean)
 return(eset)
}


SR_IAC_Eset <- function(eset, groups = NULL,threshold = 0.9){
 if(!is.null(groups)){
 group_types <- unique(as.character(groups))
 list_ests <- list()
 selected_cols <- c()
 for(i in 1:length(group_types)){
 list_ests[[i]] <- eset[,groups == group_types[i]]
 }
 for(i in 1:length(list_ests)){
 print(sprintf("Processed grup %d", i ))
 temp <- list_ests[[i]]
 temp_eset <- temp
 temp_eset <- SR_IAC_based(temp_eset,threshold = 0.9)
 selected_cols <- c(selected_cols,colnames(temp_eset))
 }
 eset_filt <- eset[,which(colnames(eset) %in% selected_cols)]
 return(eset_filt)
 }
 else{
 temp_eset <- eset
 temp_eset <- SR_IAC_based(temp_eset,threshold = 0.9)
 selected_cols <- colnames(temp_eset)
 eset_filt <- eset[,selected_cols]
 return(eset_filt)
 }
}

Combine technical replicates and recode covariate data

Combine_Replicates <- function(eset,replicates_vec){
 unique_rep <- unique(replicates_vec)
 pData_rownames <- c()
 for(i in 1:length(unique_rep)){
 if(length(which(unique_rep[i] == replicates_vec)) > 1){
 df_temp <- pData(eset)[which(unique_rep[i] == replicates_vec),]
 exp_temp <- exprs(eset)[,which(unique_rep[i] == replicates_vec),drop = F]
 exp_temp <- rowMeans(exp_temp)
 pData_rownames <- c(pData_rownames,paste(rownames(df_temp),collapse="/"))
 sel <- merging_rows(pData(eset)[which(unique_rep[i] == replicates_vec),])
 row_temp <- c()
 for(j in 1:length(sel)){
 if(is.na(sel[j])){
 row_temp <- c(row_temp,NA)

 }else if(sel[j]){
 row_temp <- c(row_temp,df_temp[1,j])
 }else{
 row_temp <- c(row_temp,paste(df_temp[,j],collapse="/"))
 }
 }
 if(i == 1){
 df_out <- data.frame(t(row_temp))
 colnames(df_out) <- colnames(pData(eset))
 exp_out <- data.frame(exp_temp)
 rownames(exp_out) <- rownames(eset)
 }else{
 df_temp <- data.frame(t(row_temp))
 colnames(df_temp) <- colnames(pData(eset))
 df_out <- rbind(df_out,df_temp)
 exp_temp <- data.frame(exp_temp)
 rownames(exp_temp) <- rownames(eset)
 exp_out <- cbind(exp_out,exp_temp)
 }
 }else{
 pData_rownames <- c(pData_rownames,rownames(pData(eset)[which(unique_rep[i] == replicates_vec),,drop=FALSE]))
 row_temp <- as.character(pData(eset)[which(unique_rep[i] == replicates_vec),])
 exp_temp <- exprs(eset)[,which(unique_rep[i] == replicates_vec),drop = F]
 exp_temp <- rowMeans(exp_temp)
 if(i == 1){
 df_out <- data.frame(t(row_temp))
 colnames(df_out) <- colnames(pData(eset))
 exp_out <- data.frame(exp_temp)
 rownames(exp_out) <- rownames(eset)
 }else{
 df_temp <- data.frame(t(row_temp))
 colnames(df_temp) <- colnames(pData(eset))
 df_out <- rbind(df_out,df_temp)
 exp_temp <- data.frame(exp_temp)
 rownames(exp_temp) <- rownames(eset)
 exp_out <- cbind(exp_out,exp_temp)
 }
 }
 }
 rownames(df_out) <- pData_rownames
 colnames(exp_out) <- pData_rownames
 pheno <- as(df_out,"AnnotatedDataFrame")
 eset_out <- new('ExpressionSet',exprs=as.matrix(exp_out),phenoData=pheno)
 return(eset_out)
}

recode_pData_After_Merging <- function(eset){
 pheno <- pData(eset)
 cols <- colnames(pheno)
 for(i in 1:length(cols)){
 if(grepl("pNm",cols[i])){
 pheno[,i] <- as.numeric(pheno[,i])
 }else if(grepl("pFac",cols[i])){
 pheno[,i] <- as.factor(pheno[,i])
 }
 }
 pData(eset) <- pheno
 return(eset)
}

Collapse probes targeting to the same gene

collapse_rows <- function(entrez_probe_map,e_set,Method = "MaxMean"){
 if(!(paste("package:","WGCNA",sep="") %in% search())){
 library("WGCNA")
 }
 mapped_probes <- mappedkeys(x)
 xx <- as.list(x[mapped_probes])
 xx_filt <- xx[rownames(e_set)]
 if(class(e_set) == "matrix" | class(e_set) == "data.frame"){
 exprs_eset_collapsed <- collapseRows(e_set,as.character(xx_filt),rownames(e_set),method=Method)
 }else{
 exprs_eset_collapsed <- collapseRows(exprs(e_set),as.character(xx_filt),rownames(e_set),method=Method)
 }
 exprs_eset_collapsed <- exprs_eset_collapsed[[1]][!is.na(rownames(exprs_eset_collapsed[[1]])),]
 return(exprs_eset_collapsed)
}

Random control sampling functions

optimum_n_selector_2 <- function(pheno_Samp,cols,list_conv,number_of_rep){
 vec_prod <- pheno_sampler_prob_vector_generator(pheno_Samp,cols,list_conv)
 #print(vec_prod)
 list_dist_to_vars <- list()
 vec_n <- c()
 vec_rep <- c()
 vec_mean <- c()
 vec_porc <- c()
 vec_list <- list()
 count <- 1
 for(i in 1:length(vec_prod)){
 print(i)
 for(j in 1:number_of_rep){
 pheno_Samp_filt <- pheno_Samp[sample(rownames(pheno_Samp),prob = vec_prod,size = i),,drop=FALSE]
 pheno_Samp_filt <- pheno_Samp_filt[,cols,drop=FALSE]
 for(k in 1:ncol(pheno_Samp_filt)){
 if(class(pheno_Samp_filt[,k]) == "numeric"){
 diff <- list_conv[[k]] - mean(pheno_Samp_filt[,k])
 vec_n <- c(vec_n,i)
 vec_rep <- c(vec_rep,j)
 vec_mean <- c(vec_mean, mean(pheno_Samp_filt[,k]))
 vec_list[[count]] <- rownames(pheno_Samp_filt)
 count <- count + 1

 }else if(class(pheno_Samp_filt[,k]) == "character"){
 if(j == 1 & i ==1 ){
 vec_porc <- as.numeric(table(factor(pheno_Samp_filt[,k],levels = c("F","M")))/sum(table(factor(pheno_Samp_filt[,k],levels = c("F","M")))))
 }else{
 num <- as.numeric(table(factor(pheno_Samp_filt[,k],levels = c("F","M")))/sum(table(factor(pheno_Samp_filt[,k],levels = c("F","M")))))
 vec_porc <- rbind(vec_porc,num)
 colnames(vec_porc) <- levels(factor(pheno_Samp_filt[,k],levels = c("F","M")))
 }
 }
 }
 }
 }
 if(is.null(vec_porc)){
 vec_porc <- data.frame(matrix(0, length(vec_n),2))
 }
 df_sal <- data.frame(vec_n,vec_rep,vec_mean,vec_porc,row.names = paste("SAMPLE",1:nrow(vec_porc),sep="_"))

 list_sal <- list(df_sal,vec_list)
 return(list_sal)
}

Intersection analysis fuctions

Compute_pvalues <- function(list,reference,threshold = 0.05,threshold_ref = 0.05,muhatA = 0.53,muhatB = 0.53){
 list_out_genes <- list()
 list_out_pvals <- list()
 for(i in 1:length(list)){
 if(i != reference){
 name_out <- paste(names(list)[reference],names(list)[i],sep="_")
 list_out_genes[[name_out]] <- IntersectionUpUpDownDown(list[[reference]],list[[i]],threshold,threshold,muhatA,muhatB)
 list_out_pvals[[name_out]] <- IntersectionPvals(list[[reference]],list[[i]],threshold_ref,threshold,muhatA,muhatB)
 }
 }
 list_out <- list(list_out_genes,list_out_pvals)
 return(list_out)
}


IntersectionUpUpDownDown <- function(ResultA,ResultB,FDRA,FDRB,muhatA,muhatB){
 ResultAfilt <- ResultA[as.numeric(as.character(ResultA$FDR)) < FDRA & abs(as.numeric(as.character(ResultA$mu.hat))) > muhatA ,]
 ResultAUp <- ResultAfilt[as.numeric(as.character(ResultAfilt$zval)) > 0 ,]
 ResultADown <- ResultAfilt[as.numeric(as.character(ResultAfilt$zval)) < 0 ,]
 ResultBfilt <- ResultB[as.numeric(as.character(ResultB$FDR))< FDRB & abs(as.numeric(as.character(ResultB$mu.hat))) > muhatB,]
 ResultBUp <- ResultBfilt[as.numeric(as.character(ResultBfilt$zval)) > 0 ,]
 ResultBDown <- ResultBfilt[as.numeric(as.character(ResultBfilt$zval)) < 0 ,]
 UPUP <- intersect(as.character(ResultAUp$symb),as.character(ResultBUp$symb))
 DOWNDOWN <- intersect(as.character(ResultADown$symb),as.character(ResultBDown$symb))
 UPDOWN <- intersect(as.character(ResultAUp$symb),as.character(ResultBDown$symb))
 DOWNUP <- intersect(as.character(ResultADown$symb),as.character(ResultBUp$symb))
 listOut <- list(UPUP,DOWNDOWN,UPDOWN,DOWNUP)
 return(listOut)
}


IntersectionPvals <- function(ResultA,ResultB,FDRA,FDRB,muhatA,muhatB){
 ResultA <- ResultA[intersect(rownames(ResultA),rownames(ResultB)),]
 ResultB <- ResultB[intersect(rownames(ResultA),rownames(ResultB)),]
 Common <- length(intersect(rownames(ResultA),rownames(ResultB)))
 ResultAfilt <- ResultA[as.numeric(as.character(ResultA$FDR)) < FDRA & abs(as.numeric(as.character(ResultA$mu.hat))) > muhatA,]
 ResultAUp <- ResultAfilt[as.numeric(as.character(ResultAfilt$zval)) > 0 ,]
 ResultADown <- ResultAfilt[as.numeric(as.character(ResultAfilt$zval)) < 0 ,]
 ResultBfilt <- ResultB[as.numeric(as.character(ResultB$FDR))< FDRB & abs(as.numeric(as.character(ResultB$mu.hat))) > muhatB,]
 ResultBUp <- ResultBfilt[as.numeric(as.character(ResultBfilt$zval)) > 0 ,]
 ResultBDown <- ResultBfilt[as.numeric(as.character(ResultBfilt$zval)) < 0 ,]
 UPUP <- length(intersect(as.character(ResultAUp$symb),as.character(ResultBUp$symb)))
 counts = (matrix(data=c(UPUP,nrow(ResultAUp) - UPUP,nrow(ResultBUp)- UPUP,Common + UPUP - nrow(ResultAUp) - nrow(ResultBUp)),nrow=2))
 testUPUP <- fisher.test(counts,alternative="greater")$p.value
 DOWNDOWN <- length(intersect(as.character(ResultADown$symb),as.character(ResultBDown$symb)))
 counts = (matrix(data=c(DOWNDOWN,nrow(ResultADown) - DOWNDOWN,nrow(ResultBDown)-DOWNDOWN,Common + DOWNDOWN - nrow(ResultADown) - nrow(ResultBDown)),nrow=2))
 testDOWNDOWN <- fisher.test(counts,alternative="greater")$p.value
 UPDOWN <- length(intersect(as.character(ResultAUp$symb),as.character(ResultBDown$symb)))
 counts = (matrix(data=c(UPDOWN,nrow(ResultAUp) - UPDOWN,nrow(ResultBDown) - UPDOWN,Common + UPDOWN - nrow(ResultAUp) - nrow(ResultBDown)),nrow=2))
 testUPDOWN <- fisher.test(counts,alternative="greater")$p.value
 DOWNUP <- length(intersect(as.character(ResultADown$symb),as.character(ResultBUp$symb)))
 counts = (matrix(data=c(DOWNUP,nrow(ResultADown) - DOWNUP,nrow(ResultBUp) - DOWNUP,Common + DOWNUP - nrow(ResultADown) - nrow(ResultBUp)),nrow=2))
 testDOWNUP <- fisher.test(counts,alternative="greater")$p.value
 listOut <- list(testUPUP,testDOWNDOWN,testUPDOWN,testDOWNUP)
 return(listOut)
}


pvalsMatrix <- function(ListOfIntersecUpUpDownDown){
 salida <- c()
 for (i in 1:length(ListOfIntersecUpUpDownDown)){
 for (j in 1:length(ListOfIntersecUpUpDownDown[[i]])){
 salida <- c(salida,ListOfIntersecUpUpDownDown[[i]][[j]])
 }
 }
 salidaMat <- matrix(salida,ncol=4,byrow=TRUE)
 colnames(salidaMat) <- c("upup","downdown","updown","downup")
 rownames(salidaMat) <- names(ListOfIntersecUpUpDownDown)
 return(salidaMat)
}


CorrecDfPval <- function(pvalsdf){
 sal <- data.frame(matrix(p.adjust(pvalsdf,method = "fdr"),nrow = length(p.adjust(pvalsdf,method = "fdr"))/4,ncol = 4,byrow = FALSE))
 colnames(sal) <- colnames(pvalsdf)
 rownames(sal) <- rownames(pvalsdf)
 return(sal)
}
